# In Silico Exploration of Novel EGFR Kinase Mutant-Selective Inhibitors Using a Hybrid Computational Approach

**DOI:** 10.3390/ph17091107

**Published:** 2024-08-23

**Authors:** Md Ali Asif Noor, Md Mazedul Haq, Md Arifur Rahman Chowdhury, Hilal Tayara, HyunJoo Shim, Kil To Chong

**Affiliations:** 1 Department of Electronics and Information Engineering, Jeonbuk National University, Jeonju 54896, Republic of Korea; 202155519@jbnu.ac.kr; 2 Research Center of Bioactive Materials, Department of Bioactive Material Sciences, Division of Life Sciences (Molecular Biology Major), Jeonbuk National University, Jeonju 54896, Republic of Korea; haqmazed@jbnu.ac.kr (M.M.H.); 201855347@jbnu.ac.kr (M.A.R.C.); 3 School of International Engineering and Science, Jeonbuk National University, Jeonju 54896, Republic of Korea; hilaltayara@jbnu.ac.kr; 4 School of Pharmacy, Jeonbuk National University, Jeonju 54896, Republic of Korea

**Keywords:** NSCLC, JBJ-125, deep learning, pharmacophore, virtual screening, molecular docking, molecular dynamics, ADMET

## Abstract

Targeting epidermal growth factor receptor (EGFR) mutants is a promising strategy for treating non-small cell lung cancer (NSCLC). This study focused on the computational identification and characterization of potential EGFR mutant-selective inhibitors using pharmacophore design and validation by deep learning, virtual screening, ADMET (Absorption, distribution, metabolism, excretion and toxicity), and molecular docking-dynamics simulations. A pharmacophore model was generated using Pharmit based on the potent inhibitor JBJ-125, which targets the mutant EGFR (PDB 5D41) and is used for the virtual screening of the Zinc database. In total, 16 hits were retrieved from 13,127,550 molecules and 122,276,899 conformers. The pharmacophore model was validated via DeepCoy, generating 100 inactive decoy structures for each active molecule and ADMET tests were conducted using SWISS ADME and PROTOX 3.0. Filtered compounds underwent molecular docking studies using Glide, revealing promising interactions with the EGFR allosteric site along with better docking scores. Molecular dynamics (MD) simulations confirmed the stability of the docked conformations. These results bring out five novel compounds that can be evaluated as single agents or in combination with existing therapies, holding promise for treating the EGFR-mutant NSCLC.

## 1. Introduction

**Figure 1:**
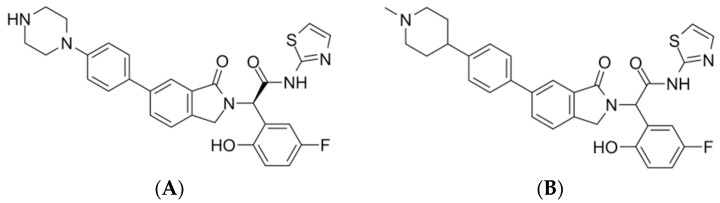
(**A**) JBJ-125. (**B**) JBJ-063.

Lung cancer is a major global health concern owing to its high mortality rates. Non-small cell lung cancer (NSCLC) is the most prevalent, accounting for over 80% of lung cancer cases [1]. However, the effectiveness of early-stage treatment with chemotherapeutic agents targeting wild-type epidermal growth factor receptor (EGFR) in NSCLC remains uncertain, as previous research has suggested limited benefits for patient survival [2]. EGFR is a transmembrane protein belonging to the ERBB (erythroblastic leukemia viral oncogene homologue) family of receptor tyrosine kinases (RTKs) [3]. The EGFR family comprises four members: ERBB1, ERBB2, ERBB3, and ERBB4 [4]. EGFR contains extracellular ligand-attachment domains and is divided into four subdomains (I, II, III, and IV). Subdomains I and III, also known as L1 and L2, are responsible for binding growth factors, whereas subdomains II and IV, or CR1 and CR2, facilitate protein dimerization [5]. First- and second-generation EGFR tyrosine kinase inhibitors (TKIs) were initially designed to target the ATP binding site but faced challenges due to resistance [6]. Although third-generation TKIs have been developed to address this issue, they face challenges, such as C797S mutation emergence [6]. Consequently, alternative and effective treatment strategies should be urgently explored. Allosteric site targeting is a promising approach in this regard [7]. It involves binding to regions other than the active site, thereby influencing protein conformation and downstream signaling pathways [8]. By targeting allosteric sites, EGFR activity and downstream signaling may be inhibited, thereby hindering cancer cell proliferation. In addition, adverse effects associated with the existing treatments for NSCLC may be overcome [9]. Previous studies have suggested that Leu747, Met766, Leu777, Leu788, Ile789, Met790, Phe856, and Asp855 constitute an EGFR allosteric site [8]. Another study by Singh et al. identified Lys745, Leu788, Thr854, Asp855, and Phe856 as the amino acids that interact with potential allosteric inhibitors [10]. So, targeting these residues can be a way to develop potential allosteric inhibitors. Recent studies have investigated compounds such as JBJ-125 (Figure 1A) and JBJ-063 (Figure 1B), which show promise for overcoming resistance mutations like L858R/T790M/C797S. Beyettet. al. reported the synergistic effect of JBJ-125 and osimertinib against TKI resistance [11].

Now, to investigate potential new compounds, in silico techniques like molecular docking are often applied, which helps in observing the interactions such as hydrogen bonds, hydrophobic bonds, pi-pi stacking, etc. [12]. This study aimed to identify distinguished derivatives, referencing JBJ-125. By targeting the allosteric sites, we aimed to contribute to the development of effective therapies for NSCLC, particularly for overcoming drug resistance.

## 2. Result

### 2.1. Pharmacophore Model Generation

**Figure 2:**
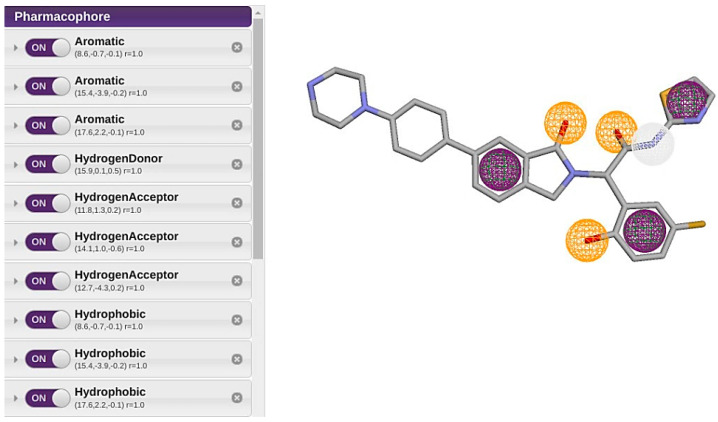
Pharmacophore model with its features.

The pharmacophore model was constructed based on the structural features of the reference ligand JBJ-125, a known potent EGFR mutant selective inhibitor (PDB 5D41), using the Pharmit tool. Ten pharmacophoric features (Table 1), including three aromatic rings, one hydrogen bond donor, three hydrogen bond acceptors, and three hydrophobic rings (Figure 2) were identified. By employing the default parameters within the Pharmit server, the Zinc database was screened using this pharmacophore model.

### 2.2. Virtual Screening

The Zinc database was virtually screened using the developed pharmacophore model and the default protocol embedded within Pharmit. This screening process yielded a collection of 16 hits that were retrieved from the database of 13,127,550 molecules and 122,276,899 conformers. In addition to these 16 hits identified as possible new compounds, JBJ-125 was included as a reference compound for further analysis (Table 2). Subsequently, all compounds were rigorously evaluated against Lipinski’s rule of five, ADMET analysis, and other pertinent in silico investigations.

### 2.3. Pharmacophore Validation

Pharmacophore validation was performed by deep decoy. The overall process assesses the hypothesis to discriminate among the active compounds and inactive decoys. The final dataset was prepared with 16 known active molecules, screened from 13,127,550 molecules and 122,276,899 conformers. From the assessment, we found an ROC (Receiver Operating Characteristic) value of 0.778, indicating better model quality and effectiveness. An Area Under the Receiver Operating Characteristic Curve (AUC-ROC) value of 0.5 suggests no discriminative power (equivalent to random guessing), while 1.0 indicates perfect classification [13]. Three types of metrics were used to evaluate the model. Performance metrics -which evaluates the ability of machine learning models to correctly classify compounds as actives or decoys. Higher values indicate better performance in distinguishing between actives and decoys [14]. Property matching metrics- which evaluates how well the properties of decoys match those of the actives, to ensure that decoys are chosen based on similar properties to actives. Thus, these metrics ensure that the decoys are appropriate controls by having similar properties to the actives. This is important for the validity of the screening process [15]. Finally, the structural similarity metrics, which evaluates the structural similarity between actives and decoys. These metrics ensure that decoys structurally resemble actives, which is important for the validity of structure-based screening methods [15].

### 2.4. ADMET Properties

**Figure 3:**
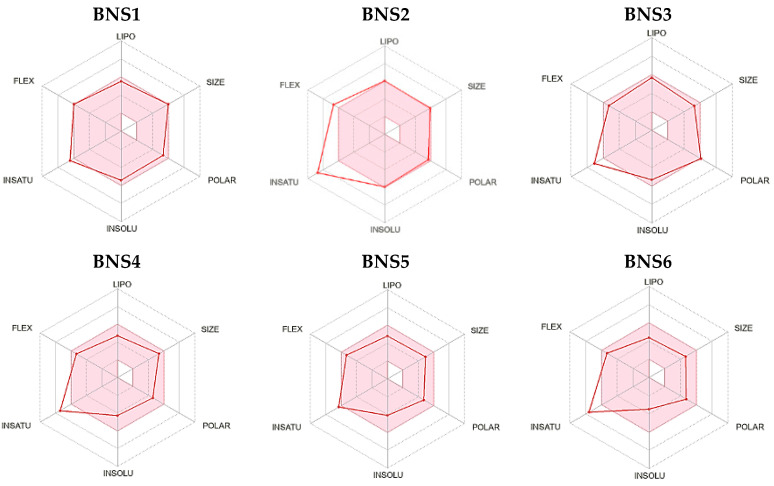

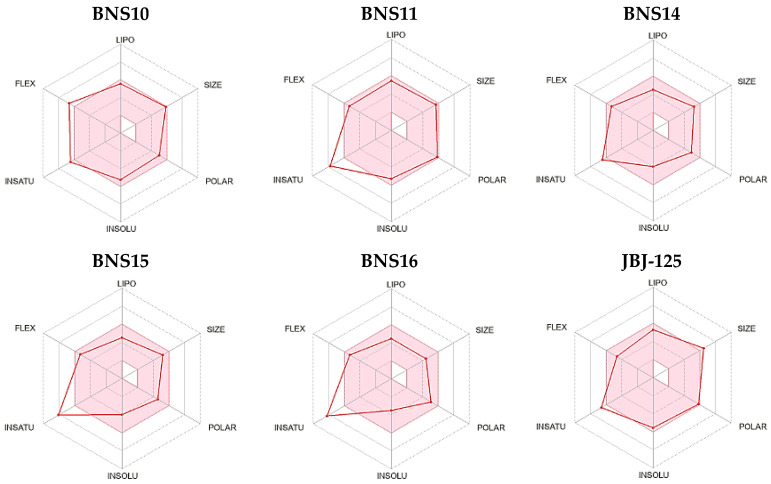
The radar of bioavailability prediction is displayed in the pink area. It shows the range of physicochemical properties optimum for oral bioavailability. Lipophilicity (−0.7 < XLOGP3 < +0.5), size (150 < MW < 500 g/mol), polarity (20A2 < TPSA < 130A2), insolubility (0 < Log S (ESOL) < 6), instauration (0.25 < Fraction Csp3 < 1), and flexibility (number of rotatable bonds < 9).

To evaluate the ADMET properties, all pharmacophore-derived compounds were assessed alongside the reference compound JBJ-125, which served as the standard. Various parameters including bioavailability radar and fundamental physicochemical properties such as molecular weight, lipophilicity, water solubility, metabolic characteristics, and drug likeliness were examined. The consistent bioavailability pattern of the pharmacophore-derived compounds BNS1–BNS6, BNS10–BNS11, and BNS14–BNS16 is comparable to that of the reference compounds. Figure 3 illustrates the distribution of these compounds with respect to their bioavailability.

Analysis of the basic physicochemical properties (Table S1) revealed that the molecular weights of all the compounds except BNS13 were below 500 g/mol. As indicated by the consensus Log P values (Table S2), the lipophilicity of the screened compounds was 2.22 to 4.32. In comparison, the Log P value of JBJ-125 was 3.42.

Assessment of water solubility patterns (Table S3) revealed that, based on the Log S(ESOL) class categorization, all compounds were soluble, with most being moderately soluble. However, according to the Ali solubility classification, most pharmacophore-derived compounds exhibited poor water solubility, a trend consistent with the SILICOS-IT class category, in which all compounds, including the reference, demonstrated poor water solubility. All compounds, along with the reference compound except BNS2, BNS3, BNS11, and BNS13 demonstrated high absorption in the gastrointestinal tract (GI, Table S4). Notably, none of these compounds permeated across the blood–brain barrier (BBB). Most compounds, including BNS1, BNS4, BNS6, BNS10, and BNS13–BNS16, along with the reference compounds, are potential permeability glycoprotein (P-gp) substrates. BNS8 inhibited only one and BNS14 inhibited two out of five cytochrome P450 (CYP) isoforms. Conversely, all other compounds inhibited a minimum of three out of the five isoforms, whereas reference compounds inhibited four isoforms. Moreover, all compounds, except for BNS13 and reference compound JBJ-125, adhered to Lipinski’s rule of five (Table 3). Additionally, all compounds exhibited a bioavailability score of 0.55. Among the compounds, BNS1-BNS6, BNS11, and BNS14–BNS16 showed no PAIN and BRENK alerts, whereas others displayed one or both alerts. The reference compound JBJ-125 presented one PAIN alert.

In the PROTOX study, hepatotoxicity, respiratory toxicity, carcinogenicity, immuno-toxicity, mutagenicity, etc., were analyzed (Table 4). PROTOX works on the similarity method, which is based on the fact that structurally similar molecules are likely to exhibit similar toxic profiles [16]. In toxicity classification, most compounds (BNS1, BNS5 to BNS7, BNS10, BNS11, and BNS13–BNS16) were categorized into level IV toxicity classes. As for BNS3, BNS8, BNS9, and BNS12, they were classified as level V toxicity classes, BNS4 as level III, BNS2 as level VI, and JBJ-125 had toxicity level IV.

Therefore, from the absorption, distribution, metabolism, and excretion (ADME) and PAIN (Pan Assay Interference) and BRENK alert analysis, we filtered BNS1-BNS6, BNS11, and BNS14–BNS16 as they were likely to possess a structurally promising moiety by not eliciting false-positive responses (PAIN alert). Usually, PAIN alert holding molecules contain substructures, which are likely to produce false positive biological results regardless of the target protein [17], thereby, reducing the likelihood of putative toxicity or metabolic instability (BRENK alert). The bioavailability ranges of these compounds were similar compared to that of the reference compound. Upon comparing the 10 compounds obtained after filtering through the PAIN and BRENK alert analysis with the 11 compounds identified from the bioavailability radar, we found overlapping compounds, except BNS10. Further scrutiny revealed that BNS10 possessed a BRENK alert, leading to its exclusion. Consequently, we selected 10 compounds that matched both the bioavailability radar and drug likeliness criteria and from the Protox toxicity class classification, which were in a considerable range; thus, they were considered for further evaluation, specifically molecular docking. 

### 2.5. Molecular Docking Validation

**Figure 4:**
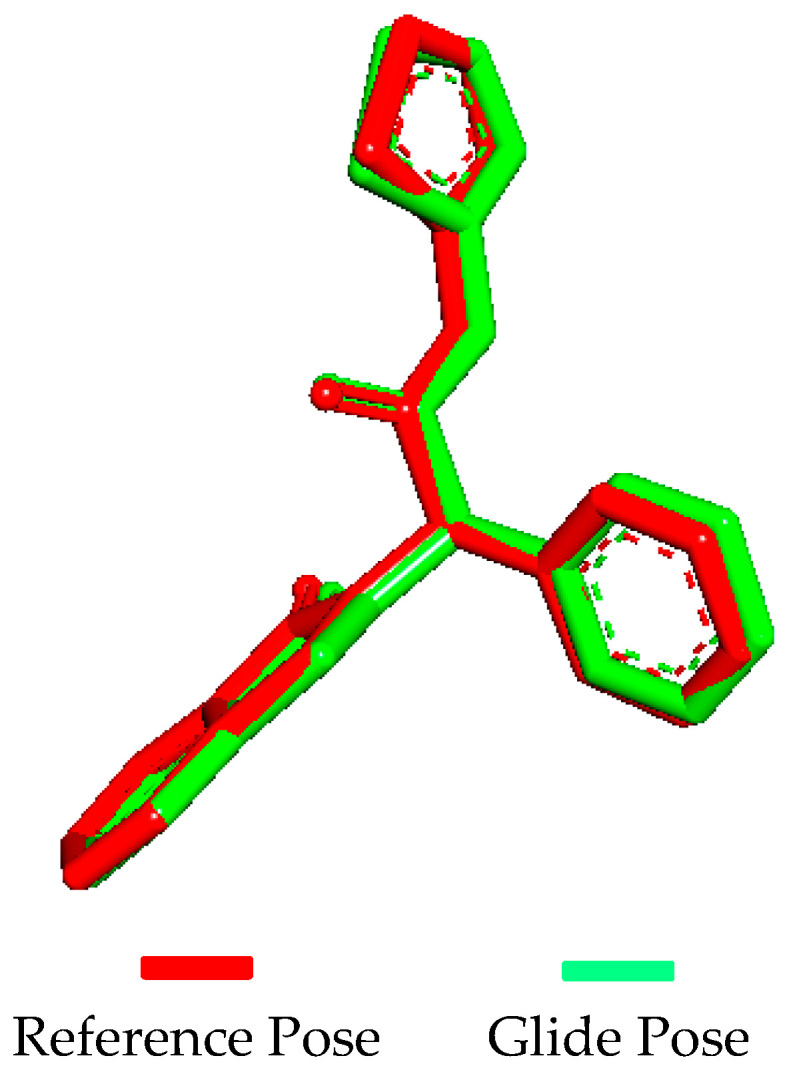
Superimposing of the Glide pose over the reference pose.

Molecular docking validation was conducted using Glide by docking the extracted native ligand (57N) to the EGFR protein (PDB ID: 5D41) and superimposing the docked ligand. Superimposition revealed that the docked ligand conformation was nearly identical to that of the native co-crystallized ligand (Figure 4), with a 0.998 root mean square deviation (RMSD) value.

### 2.6. Molecular Docking

The molecular docking results of the 10 compounds identified from the ADMET tests are presented in Table 5.

From the docking score, pharmacophore-derived compounds ranged from −9.692 to −11.625 (Table 5) and for the reference compound JBJ-125 it was −11.119. So, compared to the reference, BNS1 (−11.625) and BNS16 (−11.237) showed better docking scores. Also, considering the total amino acid interactions, JBJ-125 had 26 interactions, whereas BNS1 and BNS2 both had a maximum of 29 amino acid interactions. Apart from this, BNS3, BNS4, and BNS11 showed 27 amino acid interactions, which are more than reference JBJ-125 (Table 5). So, based on the docking score and number of amino acid interactions, BNS1–BNS4, BNS11, and BNS16 were selected for molecular dynamics simulation.

**Figure 5:**
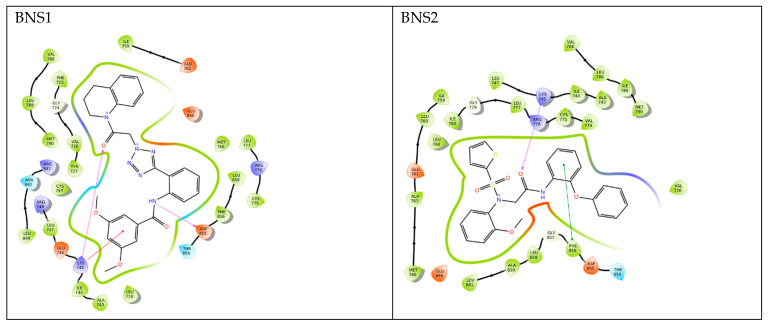

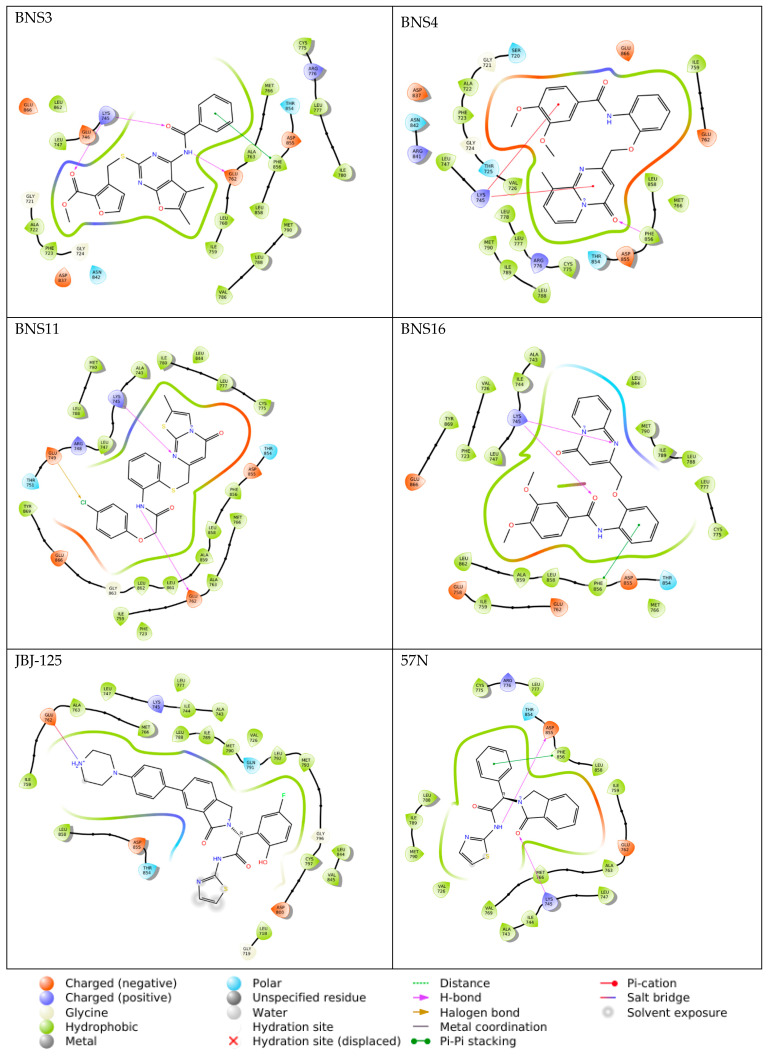
Molecular docking interactions. Specific types of interactions shown with specific marks.

From the interaction category (Figure 5), it was observed that all compounds including the reference have shown hydrophobic interaction with “LEU747, ILE759, MET766, LEU777, LEU788, MET790, PHE856, and LEU858”. Regarding hydrogen bond interactions we found that all pharmacophore-derived compounds (except BNS4) showed a hydrogen bond interaction with LYS745. Polar interaction was observed with THR854 among all the pharmacophore-derived and reference compounds. In negative charge interactions, JBJ-125 showed interaction with ASP800, ASP855 and GLU762 but all the other pharmacophore-derived compounds formed interaction with ASP855, GLU762, and GLU866. Therefore, from the molecular docking experiment, we found that our compounds interacting with amino acids mostly matched with the earlier discussed amino acids from the previous findings [8,10]. Additionally, it was observed that JBJ-125 formed a salt bridge interaction with GLU762. Pi cation interactions were observed among BNS1 and BNS4 with LYS745 and pi-pi stacking was observed with PHE856 among BNS2, BNS3, and BNS16.

Upon further analysis, it was observed that the pharmacophore-derived compounds BNS1, BNS3, BNS4, and BNS16 had the presence of a benzamide group in common and interesting interactions were formed with the benzamide group. For example, the benzene ring of the benzamide group was found to form a pi-cation interaction with LYS745 in BNS1 and BNS4 and pi-pi stacking interactions with BNS3 (Table S5). Considering the carbonyl group present in benzamide, BNS3 and BNS16 had a hydrogen bond interaction with LYS745 (Table S5). Again, from the amide group, hydrogen bond interactions were observed with ASP855 in BNS1 and with GLU762 in BNS3. On the other hand, BNS2 and BNS11 had an acetamide group in common. Here, with the acetamide group, LYS745 had a hydrogen bond interaction with the carbonyl group of BNS2 and BNS11.

### 2.7. Induced Fit Docking

By using Induced Fit Docking (IFD), it is possible to create multiple poses for the ligand–protein complex. Here, multiple conformational alterations matching the receptor –ligand position are conducted, followed by ranking the poses based on the IFD score for the identification of the ideal structure of the docked complex. In this study, using IFD, we compared the IFD scores of pharmacophore-derived compounds with the reference compound JBJ-125 to investigate the ideal ligand posture. All scores are listed in Table 5. From Table 5, we can find that the IFD score of JBJ-125 was −659.97 whereas, from the compounds obtained from pharmacophore, the IFD score range varied from −663.75 to −673.11, indicating the better performance of IFD of the pharmacophore-derived compounds. The highest IFD score was observed in BNS16 (−673.11) and the lowest in BNS2 (−663.75). With induced-fit docking, it is possible to generate multiple ligand-receptor structures along with certain conformational changes made by the receptor to receive a ligand. Therefore, this comprehensive technique helps in identifying promising ligand-receptor combinations for additional studies.

### 2.8. Molecular Dynamics Simulation

Molecular dynamics (MD) simulations were performed for 100 nanoseconds (ns) to evaluate the stability of ligand binding with proteins along with complex flexibility [18]. The tested compounds’ binding stability and protein–ligand complex flexibility were observed utilizing the root mean square deviation (RMSD) and root mean square fluctuation (RMSF), respectively. In addition, the radius of gyration (rGyr) and molecular surface area (MolSA) were also analyzed to observe the nature of ligand extendedness and molecular surface calculation respectively. 

**Figure 6:**
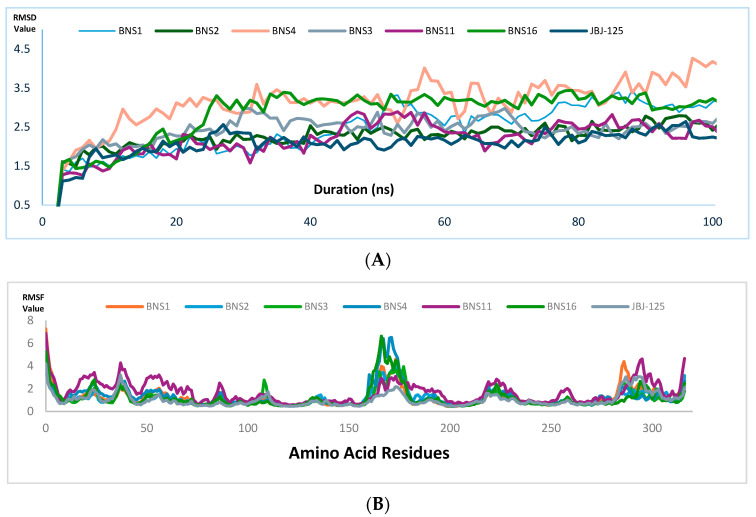
(**A**) Protein backbone RMSD graph. (**B**) Protein RMSF graph.

**Figure 7:**
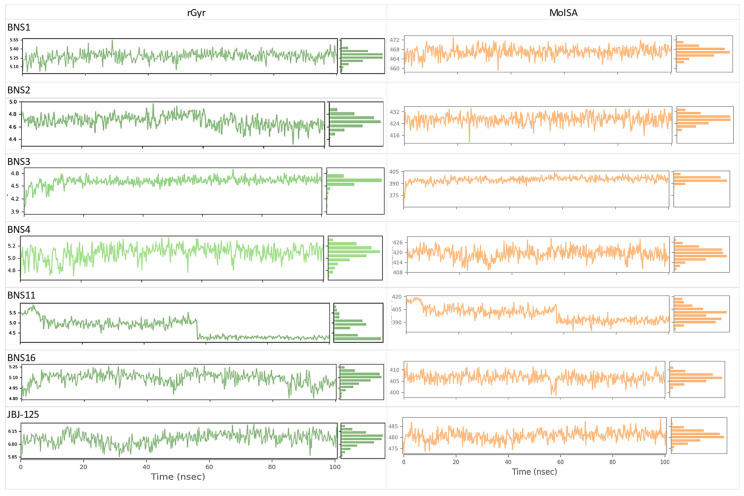
rGyr and MolSA of the pharmacophore-derived compounds and reference compound.

From the molecular dynamics simulation, for JBJ-125, the average RMSD of the protein backbone atom was 2.204 Å (Figure 6A), with a maximum of 2.92 Å at 75.40 ns. The backbone RMSD was almost stable and slight fluctuation was observed within the 68 to 76 ns range. The average RMSD of ligand fit to protein was 2.421 Å with a maximum value of 3.44 Å at 18.80; other than this, the RMSD was almost stable. Overall, the average protein–ligand complex RMSD was below 3.00 Å, indicating a good stability pattern. The protein RMSF value average was 1.032 Å, the average rGyr value was 6.063, and MolSA was 480.72 Å^2^ (Figure 7).

For BNS1, the average RMSD of the protein backbone was 3.144 Å (Figure 6A) and the average RMSD of ligand fit to protein was 2.97 Å. For the protein backbone, it took around 20 ns to reach a stable point and after that, it was almost stable through the 100 ns run; an almost similar pattern was also observed in ligand RMSD. The overall ligand protein RMSD was close to 3.00 Å. The protein RMSF value average was 1.210 Å (Figure 6B). The rGyr value average was 5.27 Å, which was better than JBJ-125, and the average MolSA value was 466.93 Å^2^ (Figure 7).

For BNS2, the protein backbone RMSD value average was 2.821 Å (Figure 6A). The RMSD distribution pattern of the BNS2 protein backbone was almost similar to BNS1. The ligand RMSD average was 2.10 Å with minor fluctuations. This indicates the overall considerable RMSD of the protein–ligand complex. The average protein RMSF (Figure 6B) value was observed at 1.171 Å. The rGyr was observed at 4.688 Å, which was better than the reference compound. The average MolSA value was 426.87 Å^2^ (Figure 7).

In BNS3, the observed protein backbone RMSD average was 2.805 Å (Figure 6A), and ligand RMSD had an average of 4.155 Å. For protein backbone RMSD, initial fluctuation was seen within 20 ns; after that, similar distribution was observed. In ligand RMSD, after 40 ns, stable distribution was seen. Here, the ligand RMSD value observed was slightly higher than the JBJ-125. The protein RMSF value average was 1.09 Å (Figure 6B). Also, the average rGyr was 4.605 Å, which was better than JBJ-125, and the MolSA average was 394.94 Å^2^ (Figure 7).

In BNS4, the average protein backbone RMSD value was 4.014 Å (Figure 6A) and the ligand RMSD was 4.713 Å. The average protein RMSF value was 1.309 Å (Figure 6B) but the highest fluctuation crossed 5 Å. The rGyr value observed was 5.08 Å, whereas MolSA was 419.194 Å^2^ (Figure 7)

In BNS11, the protein backbone RMSD average was observed at 2.95 Å (Figure 6A) but in the protein backbone RMSD, among the overall distribution, fluctuation was frequently raised above 3.0 Å. The ligand RMSD average was 4.706 Å but after 10 ns to the rest, the overall distribution was above 4.0 Å, indicating less ligand protein binding compared to JBJ-125. The average protein backbone RMSF was 1.68 Å (Figure 6B). The rGyr was 4.71 Å and the^,^ MolSA average was 399.08 Å^2^ (Figure 7).

For BNS16, the protein backbone RMSD average was 2.67 Å (Figure 6A); after 30 ns to the rest, the average distribution was below 3.0 Å. The ligand RMSD average was 2.280 Å; after initial fluctuation, the RMSD graph declined to 2.50 Å till the first 50 ns. During the last 50 ns, distribution was observed below 2.5 Å. The protein RMSF average was 1.180 Å^2^ (Figure 6B). The average rGyr was 5.08 Å and the average MolSA was 406.49 Å^2^ (Figure 7).

For all the compounds obtained from the pharmacophore, the rGyr value score was lower than the reference compound, indicating that the pharmacophore-derived compounds will undergo less conformational change within the active site than the reference one [19]. The MolSA value of the compounds indicates the polarity of the compounds, which is competitive toward the reference [19]. The post-MD simulation interaction is presented in the Supplementary Figure S1 and the data are presented in Tables S6–S10.

Compared to the reference compound, BNS2 and BNS16 had a similar protein–ligand RMSD value average, within the range below 3.0 Å, indicating a stable complex [20]. For BNS1, the ligand RMSD average was 2.97 Å and the protein RMSD value average was 3.144 Å, which is not significantly higher than the acceptable range. In BNS3 and BNS11, the average ligand RMSD value was slightly higher than 3.0 Å (i.e., 4.155 Å and 4.706 Å) but not significantly different; thus, BNS1, BNS3, and BNS11 can also be considered for further evaluation. But, in BNS4, both the protein and ligand RMSD value average was over 4.00 Å; thus, excluding this, we have BNS2, BNS16, BNS1, BNS3, and BNS11 (Figure S2) as potential candidates for further evaluation.

## 3. Discussion

After pharmacophore modeling and virtual screening, we validated the pharmacophore model using deep learning techniques and found considerable results. The physicochemical attributes of pharmacophore-derived compounds were comprehensibly analyzed utilizing SWISS ADME. Lipophilicity, a fundamental determinant of drug absorption, was meticulously evaluated from the Log P_o/w_ (ranging from 2.22–4.32 across the compound set). These positive values signify favorable lipophilic characteristics, indicating potential gastrointestinal absorption [21,22]. Furthermore, the assessment of solubility, a pivotal parameter governing drug bioavailability, revealed a collective trend toward poor water solubility among the pharmacophore-derived compounds and reference standards. Despite this, the observed lipophilicity suggests the prospect of substantial oral absorption, facilitating systemic distribution and eventual therapeutic action [23,24]. This intricate interplay between lipophilicity and solubility underscores the nuanced pharmacokinetic profile of the identified compounds, warranting further exploration to elucidate their therapeutic potential with precision and depth.

The assessment of Absorption, Distribution, Metabolism, and Excretion (ADME) properties constitutes a pivotal aspect in delineating the pharmacological behavior of potential drug candidates [25]. Particularly, for orally administered drugs, efficient absorption within the gastrointestinal tract (GIT) is of paramount importance for optimizing pharmacokinetic parameters. Conversely, the blood–brain barrier (BBB) serves as a pivotal physiological barrier that selectively regulates the entry of substances into the central nervous system (CNS) [26]. A comprehensive analysis of ADME properties revealed compelling insights into the pharmacokinetic profile of the identified compounds. Notably, except for BNS2, BNS3, BNS11, and BNS13, the evaluated compounds exhibited high gastrointestinal absorption rates, indicating favorable oral bioavailability. Furthermore, the absence of blood–brain barrier permeation among the investigated compounds suggests a reduced likelihood of adverse effects within the CNS, thus augmenting their safety profile for potential therapeutic applications. These findings underscore the potential utility of the identified compounds as orally administered agents with favorable pharmacokinetic attributes and minimal CNS-related side effects.

P-glycoprotein (P-gp) serves as a pivotal efflux transporter, facilitating substrate translocation from intracellular to extracellular compartments, thereby mitigating the potential toxic effects of compounds [27,28]. In our in silico investigation, we tested the P-gp substrate affinity of the identified compounds to elucidate their potential pharmacokinetic interactions. Notably, JBJ-125 exhibited P-gp substrate positivity, indicating their propensity to interact with this efflux transporter. Similarly, BNS1, BNS4, BNS6, BNS10, and BNS13-BNS16 also demonstrated P-gp substrate positivity. These observations shed light on the potential pharmacokinetic behavior of the identified compounds, particularly their interaction with P-gp and subsequent implications for drug disposition and efficacy. These insights will be instrumental in guiding further pharmacological evaluation and therapeutic applications of the identified compounds.

Understanding the intricate interactions between compounds and the cytochrome P450 (CYP) system is crucial to elucidating the pharmacokinetic profiles of potential drugs. These interactions play a pivotal role in mediating the biotransformation and elimination of drugs from the systemic circulation [17]. In this study, we examined the inhibitory potential of the identified compounds against various CYP isoforms to elucidate their pharmacokinetic implications. Our findings revealed that BNS8 and BNS14 inhibited only one and two CYP isoforms, respectively. Interestingly, the remaining compounds inhibited a minimum of three CYP isoforms. Comparative analysis of the reference compound JBJ-125 demonstrated a striking similarity in the inhibitory patterns, underscoring the consistency in pharmacokinetic behavior across the compounds. These observations underscore the importance of assessing CYP-mediated drug interactions in predicting the pharmacokinetic profile and potential drug–drug interactions of novel compounds. Such insights are invaluable for guiding further pharmacological investigations and optimizing therapeutic strategies.

The identification of structurally promising moieties and assessment of potential toxicity are critical steps in the preclinical evaluation of novel compounds. In the study, we used PAIN and BRENK to identify the structural motifs associated with false-positive responses in silico and putative toxicity, chemical reactivity, and metabolic instability [29,30]. Our analysis revealed that BNS1- BNS6, BNS11, and BNS14–BNS16 exhibited no PAIN or BRENK alerts. Conversely, JBJ-125 exhibited one PAIN alert. Furthermore, the PROTOX study indicated that all other pharmacophore-derived compounds, except BNS4, demonstrated toxicity levels below III, suggesting their potential safety for further evaluation [31]. Based on the bioavailability radar and drug-likeness properties, we selected 10 compounds (BNS1-BNS6, BNS11, and BNS14–BNS16) for subsequent molecular docking studies.

From the docking studies, we found that compounds BNS1 to BNS4 and BNS11 showed more amino acid interactions and BNS1 and BNS16 had docking scores higher than reference JBJ-125. From the induced fit docking; we found all the pharmacophore-derived compounds had higher IFD scores. Compared with JBJ-125, we can observe that, like JBJ-125, BNS1, BN2, BNS3, BNS4, BNS11, and BNS16 had common positive charge interactions with LYS745. Here, we do not see any additional interaction between JBJ-125 and LYS745 but in BNS1 there is one hydrogen bond interaction and one pi cation interaction with LYS745, one hydrogen bond interaction with BNS2 and BNS3, two pi cation interactions with BNS4, BNS11, and BNS16 present, which shows the stronger bond formation of these compounds compared to JBJ-125. JBJ-125 had negative charge interactions with GLU762, so was seen among the other compounds also. Notably, JBJ-125 had a salt bridge interaction with GLU762. Additionally, BNS3 and BNS11 had formed one hydrogen bond interaction with GLU762, indicating their competitiveness with JBJ-125. Also, in the introduction part, THR854, Asp855, and Phe856 were discussed as key important amino acid residues for the allosteric site [8,10]. Here, JBJ-125, THR854, Asp855, and Phe856 formed polar, charge-negative, and hydrophobic interactions, respectively. A similar pattern was observed among all the other compounds also as well. Additionally, we observed that in BNS1, a hydrogen bond interaction was formed with ASP855 and a pi-pi stacking interaction was formed with PHE856 in BNS2, BNS11, and BNS16, stating stronger interaction of these compounds than JBJ-125. Previous studies have shown that afatinib and erlotinib showed docking scores of −7.69 and −7.37, respectively, against EGFR [32,33], whereas, our pharmacophore-derived compounds showed better docking scores than them indicating their better binding affinity and selectivity.

Compound BNS1 bears a 1,2,3,4-tetrahydroquinoline scaffold. In previous studies, quinazoline derivatives containing the 1,2,3,4-tetrahydroquinoline moiety demonstrated significant inhibitory activity against EGFR kinase, comparable to the positive control, afatinib [34]. This suggests that BNS1 could potentially exhibit strong EGFR inhibitory effects, making it a promising candidate for further experiments.

Also, Compound BNS3 features a thiazolo[3,2-a]pyrimidine scaffold. Another study reported that a novel series of naphtho[2′,3′:4,5]thiazolo[3,2-a]pyrimidine hybrids were synthesized and evaluated for their topo IIα/EGFR inhibitory activities [35]. Compounds 6i, 6a, and 6c from this series showed superior cytotoxic activity compared to doxorubicin and erlotinib against tested cancer cell lines. Molecular docking studies revealed that compound 6a forms the same hydrogen bond interaction with LYS 745 as observed with BNS3 in our study. This structural similarity and interaction suggest that BNS3 may also exhibit potent EGFR inhibition and could offer enhanced efficacy in treating cancers with EGFR involvement. Both BNS1 and BNS3 show potential for strong EGFR inhibitory activity due to their structural resemblance to compounds that have demonstrated efficacy in preclinical studies. This enhances their prospects as effective EGFR inhibitors.

Along with these tests, the considerable MD simulation pattern increases the acceptance of our compounds. Moreover, all these compounds showed interactions with the key important amino acid residues regarded as potential allosteric sites as mentioned earlier. Thus, our final compounds can be considered for further experiments as a better therapeutic choice compared to JBJ-125.

## 4. Material and Methods

Virtual experimentation was started with pharmacophore design and virtual screening using Pharmit [36], followed by the ADMET test using SWISS ADME and PROTOX 3.0 [17,31]. For docking, the glide function was used to perform a systematic search for the conformational, orientation, and positional space of the ligand in the binding pocket [37]. A molecular dynamics study was performed using Desmond in the Schrodinger molecular modeling suite [18].

### 4.1. Pharmacophore Designing/Modeling

A pharmacophore is an exposure of the drug-likeness of a molecule to its steric and electronic features, which are required to ensure optimal intermolecular interactions with a specific biological target, that is, a protein or enzyme, and inhibit or block its activity [38]. The pharmacophore technique can be used to facilitate drug development while searching large libraries or databases. In this study, the structure-based pharmacophore for the allosteric site of PDB ID:5D41 was generated using JBJ-125, the active inhibitor. The pharmacophore was generated using the free online server Pharmit, an open tool available at (http://pharmit.csb.pitt.edu, accessed on 13 June 2024). The value specification we used here are aromatic ring 1 (X: 8.6, Y: −0.7, Z: −0.1), aromatic ring 2 (X: 15.4, Y: −3.9, Z: −0.2), aromatic ring 3 (X: 17.6, Y: 2.2, Z: −0.1), hydrogen bond donor (X: 15.9, Y: 0.1, Z: 0.5), hydrogen bond acceptor 1 (X: 11.8, Y: 1.3, Z: 0.2) hydrogen bond acceptor 2 (X: 14.1, Y: 1.0, Z: −0.6), hydrogen bond acceptor 3 (X: 12.7, Y: −4.3, Z: 0.2), hydrophobic bond 1 (X: 8.6, Y: −0.7, Z: −0.1), hydrophobic bond 2 (X: 15.4, Y: −3.9, Z: −0.2), and hydrophobic bond 3 (X: 17.6,Y: 2.2, Z: −0.1).

### 4.2. Pharmacophore-Based Virtual Screening

In computational drug development and discovery processes, pharmacophore-based virtual screening is one of the most important steps for searching large libraries to identify LEADS against specific targets. Several tools and servers are available for pharmacophore-based virtual screening. Here, we used Pharmit, a free online server with an algorithm that can screen compound libraries based on the pharmacophore model or molecular shape and rank the results by energy minimization [36]. Using Pharmit, large databases of compounds can be screened based on their pharmacophoric features or molecular shapes. In this study, we screened the zinc database (https://zinc20.docking.org/, accessed on 13 June 2024) [39] based on JBJ-125 using Pharmit, and the top hits generated from the model are given in Table 6.

### 4.3. Pharmacophore Validation

To determine the model accuracy of our pharmacophore model in predicting active chemicals, pharmacophore validation was performed. Here, we used the Deep decoy dataset (https://github.com/oxpig/DeepCoy, accessed on 18 June 2024), which generates property—matching decoy molecules, using a deep learning strategy called deep coy [15]. Here, we took the active molecules SMILE and generated 100 inactive decoy structures for each active molecule.

### 4.4. ADME Profile

The absorption, distribution, metabolism, and excretion (ADME) profile of the selected compounds was determined using SwissADME. The freely accessible SwissADME web tool (http://www.swissadme.ch/, accessed on 19 June 2024) is the most relevant computational method for providing a global appraisal of the pharmacokinetic profiles of small molecules. These methods were selected by web tool designers for robustness and ease of interpretation to enable efficient translation into medicinal chemistry [17]. Additionally, hepatotoxicity, neurotoxicity, carcinogenicity, immune-toxicity, mutagenicity, cytotoxicity, and toxicity were predicted using PROTOX 3.0 (ProTox-3.0-Prediction of Toxicity of chemicals) available in (https://tox.charite.de/protox3/, accessed on 19 June 2024) [31].

### 4.5. Ligand Preparation

For ligand preparation, we designed a structure-based pharmacophore, targeting the allosteric site of PDB ID-5D41 focusing on JBJ-125, its active inhibitor. The pharmacophore was generated using the free online server Pharmit. Using the pharmacophore, we screened 16 compounds having structural similarity with JBJ-125 from the zinc database. The selected compounds were processed for energy minimization via the LigPrep module of Schrodinger using the OPLS3e force field [40]. The ZINC and PubChem ID of the pharmacophore-derived and reference compounds, respectively, are presented in Table 1.

### 4.6. Protein Preparation

We selected a mutant-selective EGFR protein structure targeting T790M and C797S mutations (PDB ID-5D41). The PDB structure was downloaded from RCSB PDB [41]. After the protein structure was retired from RCSB PDB, it underwent protein preparation processes available in Schrodinger [42]. During the protein preparation, water molecules were removed, missing side chains were added using Prime, and all co-crystallized ligands except 57N were deleted because they represent an allosteric inhibitor. 57N was used later for generating the receptor grid. The protein energy minimization was performed using the OPLS3e force field. The Van der Waals radius scaling factor was kept at 1.0 with a partial cutoff value of 0.25. For receptor grid generation, the centroid of the workspace ligand (57N) was selected and the grid box was generated accordingly (X: −23.71, Y: 31.37, Z: 12.3).

### 4.7. Docking Simulation Validation

Docking simulation was validated by re-docking the native ligand to the receptor binding site, to validate docking analysis, reproducibility, and reliability.

### 4.8. Molecular Docking

The GLIDE operational ligand docking tool in Maestro was used to generate molecular docking [43]. In GLIDE, compounds having atom numbers more than 500 and rotatable bonds more than 100 were set to reject [44]. As the number of designed analogs and generated tautomer was less, they were screened using the standard precision (SP) method, which uses descriptors and explicit water technology. The SP method eliminates false positives and employs a protocol with a refined growth strategy [37] and for ligand sampling, the flexible option was chosen along with nitrogen inversion and ring conformation in consideration. The application of sample bias was performed to all torsions presented with attached functional groups. Also, the Epik tool was enabled to enhance the docking score [45]. Minimization of post-docking was also performed, where the number of ten poses per ligand was evaluated to report the most effective conformation.

### 4.9. Induced Fit Docking

For induced fit docking using Schrodinger, the induced fit docking module was utilized [19,28]. Here, we used the previously used receptor grid box. For conformational sampling, sample ring conformation was kept with an energy window of 2.5 kcal/mol; additionally, receptor van der Waals scaling was kept at 0.50 along with ligand van der Waals scaling at 0.50. Residue refinement was kept within 5.0 Å of the ligand poses. Glide redocking of structures was kept within 30.0 kcal/mol of the best structure with standard precision mode. Table 5 presents the outcome of the induced fit docking score.

### 4.10. Molecular Dynamics

Target–ligand complex flexibility was studied via molecular dynamics (MD) to mimic biological systems. MD simulations were performed using the Desmond tool of the Schrödinger Drug Design Suite. Based on the docking score, the ligands were subjected to MD simulations for 100 ns to study their stability. The three steps performed for the MD simulation were building the system, minimization, and MD simulation. The docked ligand–protein complex was selected and the system was modeled by a predefined solvent system—TIP3P under orthorhombic boundary conditions. System neutralization was conducted by adding counter ions and salt was added as a concentration of 0.15 M Na^+^ and Cl^−^ ions for reaching physiological circumstances and the system building was performed using OPLS3e force field. In a 100 ns run of molecular dynamics, trajectory data were taken every 50 picoseconds, energy data were captured at 1.2 picoseconds intervals, and the approximate number of frames was 500. NPT ensemble class was selected and 300 K temperature followed by 1.01325 pressures (bar) was carried out for MD simulation. Later, utilization of the simulation interaction diagram function was used for generating figures and plots to present the results. Any negative charges on the model were neutralized with sodium ions and the model was subjected to energy minimization until 25 kcal/mol/Å gradient thresholds were achieved at 300 K and 1 bar pressure via the NPT ensemble class. When conducting the MD simulation, the trajectory was recorded at 50 ps with approximately 500 frames. The complex stability was evaluated by protein and ligand RMSD (Root-Mean-Square Deviation) fluctuations, protein–ligand interactions, and contacts with various amino acids using the Simulation Event Analysis tool of Desmond [18].

## 5. Conclusions

In our study, the aim was to identify potential allosteric inhibitors to overcome the mutations that happen in EGFR NSCLC. To do it, we considered compound JBJ−125 as a reference and developed a pharmacophore-based on the features of JBJ-125 and performed a deep learning-based method to validate the pharmacophore model, followed by virtual screening. After that, we evaluated their toxicity via Swiss ADME and Protox. The screened compounds from ADMET tests undergo molecular docking, induced fit docking, and molecular dynamics studies. We found that BNS1, BNS2, BNS3, BNS11, and BNS16 have better interactions and docking scores than JBJ-125, and interactions with previously reported amino acid residues as allosteric sites were also observed among them. Recent studies indicate the capacity of JBJ-125 as a promising one to overcome resistance as a single agent or in combination with Osimertinib; hence, we believe we have potential outcomes and in vitro studies need to be performed to fully discover their therapeutic potential.

## Figures and Tables

**Table 1:** Pharmacophore model features along with X, Y, Z coordination and radius.

Feature	X	Y	Z	Radious
Aromatic Ring 1	8.6	−0.7	−0.1	1
Aromatic Ring 2	15.4	−3.9	−0.2	1
Aromatic Ring 3	17.6	2.2	−0.1	1
Hydrogen Bond Donor	15.9	0.1	0.5	1
Hydrogen Bond Acceptor 1	11.8	1.3	0.2	1
Hydrogen Bond Acceptor 2	14.1	1	−0.6	1
Hydrogen Bond Acceptor 3	12.7	−4.3	0.2	1
Hydrophobic Bond 1	8.6	−0.7	−0.1	1
Hydrophobic Bond 2	15.4	−3.9	−0.2	1
Hydrophobic Bond 3	17.6	2.2	−0.1	1

**Table 2:** List of pharmacophore-derived compounds and reference compound with code numbers.

Scheme	Compound ID	Code Number	SMILE
1	ZINC000012638703	BNS1	COc1cc(OC)cc(C(=O)Nc2ccccc2-c2nnn(CC(=O)N3CCCc4ccccc43)n2)c1
2	ZINC000016694801	BNS2	COc1ccccc1N(CC(=O)Nc1ccccc1Oc1ccccc1)S(=O)(=O)c1cccs1
3	ZINC000012777271	BNS3	COC(=O)c1occc1CSc1nc(NC(=O)c2ccccc2)c2c(C)c(C)oc2n1
4	ZINC000033067859	BNS4	COc1ccc(C(=O)Nc2ccccc2OCc2cc(=O)n3cccc(C)c3n2)cc1OC
5	ZINC000020617126	BNS5	COc1ccc(C)cc1NC(=O)c1cn(C)nc1C(=O)Nc1cc(C)ccc1OC
6	ZINC000020617150	BNS6	COc1ccccc1NC(=O)c1cn(C)nc1C(=O)Nc1ccccc1OC
7	ZINC000059488018	BNS7	Oc1ccc(Br)cc1/C=N/N=C1/c2ccccc2-c2nc3ccccc3nc21
8	ZINC000059488022	BNS8	O=[N+]([O-])c1ccc(O)c(/C=N/N=C2/c3ccccc3-c3nc4ccccc4nc32)c1
9	ZINC000059488016	BNS9	Oc1cc(Cl)ccc1/C=N/N=C1/c2ccccc2-c2nc3ccccc3nc21
10	ZINC000013577005	BNS10	COc1ccc(NC(=O)C[C@H]2C(=O)N(c3ccccc3)C(=S)N2CCc2ccccc2OC)cc1
11	ZINC000021535964	BNS11	Cc1cn2c(=O)cc(CSc3ccccc3NC(=O)COc3ccc(Cl)cc3)nc2s1
12	ZINC000059488021	BNS12	COc1ccc(O)c(/C=N/N=C2/c3ccccc3-c3nc4ccccc4nc32)c1
13	ZINC000229934991	BNS13	O=C(Nc1ccc(Cl)cc1)[C@@H]1[C@H](c2cccc([N+](=O)[O-])c2)C2(C(=O) c3ccccc3C2=O)[C@H]2c3ccccc3C=NN12
14	ZINC000041077159	BNS14	COc1cccc(C(=O)Nc2ccccc2OCc2cc(=O)n3c(ncn3C(C)C)n2)c1
15	ZINC000000831474	BNS15	COc1ccccc1NC(=O)c1nc[nH]c1C(=O)Nc1ccccc1OC
16	ZINC000033067751	BNS16	COc1ccc(C(=O)Nc2ccccc2OCc2cc(=O)n3ccccc3n2)cc1OC
17	Pubchem CID 124173751	JBJ-125	C1CN(CCN1)C2=CC=C(C=C2)C3=CC4=C(CN(C4=O)C(C5=C(C=CC(=C5)F)O)C(=O)NC6=NC=CS6)C=C3

**Table 3:** Predicted drug likeliness properties of compounds.

Compounds.	Lipinski #Violations	Ghose #Violations	Veber #Violations	Egan #ViolAtions	Muegge #Violations	Bioavailability Score	PAINS #Alert	Brenk #Alert	Lead Likeness #Violations	Synthetic Accessibility
BNS1	Yes, 0 violations	No#2	yes	yes	yes	0.55	0	0	No#3	3.75
BNS2	Yes, 0 violations	No#3	yes	No	No	0.55	0	0	No#3	3.93
BNS3	Yes, 0 Violation	Yes	Yes	No	Yes	0.55	0	0	No#3	3.73
BNS4	Yes, 0 Violations	Yes	Yes	Yes	Yes	0.55	0	0	No#2	3.32
BNS5	Yes, 0 Violations	Yes	Yes	Yes	Yes	0.55	0	0	No#2	3.04
BNS6	Yes, 0 Violations	Yes	Yes	Yes	Yes	0.55	0	0	No#2	2.81
BNS7	Yes, 0 Violations	Yes	Yes	Yes	Yes	0.55	1	1	No#2	3.3
BNS8	Yes, 0 Violations	Yes	Yes	Yes	Yes	0.55	1	3	No#2	3.37
BNS9	Yes, 0 violations	Yes	Yes	Yes	Yes	0.55	1	1	No#2	3.25
BNS10	Yes, 0 Violations	No#2	Yes	Yes	Yes	0.55	0	1	No#3	3.88
BNS11	Yes, 0 Violations	Yes	Yes	Yes	Yes	0.55	0	0	No#3	3.5
BNS12	Yes, 0 Violations	Yes	Yes	Yes	Yes	0.55	1	1	No#2	3.37
BNS13	Yes, 1 Violation	No#2	Yes	Yes	No#1	0.55	1	3	No#2	5.32
BNS14	Yes,0 Violations	Yes	Yes	Yes	Yes	0.55	0	0	No#2	3.34
BNS15	Yes, 0 Violations	Yes	Yes	Yes	Yes	0.55	0	0	No#2	2.66
BNS16	Yes, 0 Violations	Yes	Yes	Yes	Yes	0.55	0	0	No#2	3.17
JBJ-125	Yes, 1 Violations	No#2	No#2	Yes	Yes	0.55	1	0	No#2	4.26

**Table 4:** Toxicity prediction by PROTOX.

Compounds	Hepato-Toxicity	Neuro Toxicity	Respiratory Toxicity	Carcino Genicity	Immuno Toxicity	Muta Genicity	Cyto Toxicity	Toxicity Class
BNS1	Inactive	Active	Active	inactive	inactive	Moderately active	Moderately active	IV
BNS2	Inactive	Inactive	Active	Moderately inactive	inactive	inactive	inactive	VI
BNS3	Moderately active	Inactive	Moderately active	Moderately active	Moderately active	Moderately inactive	Inactive	V
BNS4	Moderately inactive	Moderately active	Active	Moderately inactive	Moderately active	Moderately active	Moderately inactive	III
BNS5	Moderately active	Moderately active	Moderately inactive	Moderately inactive	Inactive	Moderately inactive	Inactive	IV
BNS6	Moderately active	Moderately active	Moderately inactive	Moderately inactive	Inactive	Moderately inactive	Inactive	IV
BNS7	Moderately active	Moderately active	Moderately inactive	Moderately inactive	Moderately active	Moderately inactive	Moderately inactive	IV
BNS8	Moderately active	Moderately inactive	Moderately inactive	Active	Moderately inactive	Active	inactive	V
BNS9	Moderately active	Moderately active	Moderately inactive	Moderately inactive	inactive	Moderately inactive	inactive	V
BNS10	Moderately inactive	Active	Active	Moderately inactive	inactive	inactive	inactive	IV
BNS11	Moderately active	Moderately active	Active	Moderately inactive	Moderately inactive	Moderately inactive	Moderately inactive	IV
BNS12	Moderately active	Moderately active	Moderately inactive	Moderately active	Active	Moderately active	inactive	V
BNS13	Moderately active	Moderately inactive	Moderately inactive	Moderately active	inactive	Active	inactive	IV
BNS14	Moderately inactive	Moderately active	Moderately active	Moderately inactive	Active	Moderately active	inactive	IV
BNS15	Moderately inactive	Moderately inactive	Moderately inactive	Moderately active	inactive	Moderately inactive	inactive	IV
BNS16	Moderately inactive	Moderately active	Active	Moderately inactive	Moderately active	Moderately active	Moderately inactive	IV
JBJ-125	Moderately inactive	Active	Active	Moderately inactive	Active	Moderately inactive	Moderately inactive	IV

**Table 5:** Molecular docking (Glide score, IFD score, and total amino acid interactions) result.

Compound	Glide Score (Kcal/mol)	IFD Score	Total Amino Acid Interaction
BNS1	−11.625	−667.73	29
BNS2	−10.313	−663.75	29
BNS3	−9.874	−666.74	27
BNS4	−10.408	−671.39	27
BNS5	−10.217	−664.96	25
BNS6	−9.853	−664.44	23
BNS11	−10.193	−665.93	27
BNS14	−10.442	−671.61	25
BNS15	−9.692	−663.93	21
BNS16	−11.237	−673.11	24
57N	−10.388	−662.43	20
JBJ-125	−11.119	−659.97	26

**Table 6:** Top hits generated from the Pharmit model.

Compound ID	RMSD	Mass	RBnds
ZINC000012777271	0.617	437	8
ZINC000013577005	0.642	490	10
ZINC000229934991	0.687	577	5
ZINC000033067751	0.699	431	8
ZINC000012638703	0.701	499	9
ZINC000041077159	0.714	433	8
ZINC000020617150	0.750	380	8
ZINC000020617126	0.751	408	8
ZINC000033067859	0.754	445	8
ZINC000000831474	0.762	366	8
ZINC000059488016	0.797	385	2
ZINC000059488018	0.798	429	2
ZINC000059488021	0.798	380	3
ZINC000059488022	0.798	395	3
ZINC000016694801	0.807	495	10
ZINC000021535964	0.821	472	8

## Data Availability

Data are contained within the article and Supplementary Materials.

## Supplementary Materials

The following supporting information can be downloaded at https://www.mdpi.com/article/10.3390/ph17091107/s1. The supporting information of physicochemical properties (Table S1), lipophilicity (Table S2), water solubility (Table S3), metabolism properties (Table S4), key interactions (Table S5), protein backbone RMSD (Table S6), ligand fit to protein RMSD (Table S7), protein RMSF (Table S8), rGyr value (Table S9), and MolSA (Table S10) and Figures post MD simulation interactions (Figure S1) and final compounds (Figure S2) are provided.
