# Supplementary material for: In Silico Exploration of Novel EGFR Kinase Mutant-Selective Inhibitors Using a Hybrid Computational Approach
Source: Pharmaceuticals (Basel). 2024 Aug 23;17(9):1107. doi: 10.3390/ph17091107 (PMC11434943; doi:10.3390/ph17091107)
Supplement: Supplementary file 1 [file pharmaceuticals-17-01107-s001.zip › pharmaceuticals-3104293-supplementary.pdf]

Table S1. Basic physicochemical properties of the experimental compounds

Compounds	Formula	MW g/mol	Num. heavy atoms	Num. arom. Heavy atoms	Fraction Csp3	Num. rotatable bonds	Num. H-bond acceptors	Num. H-bond donors	Molar Refractivity
CMP1	C27H26N6O4	498.53	37	23	0.22	9	7	1	141.04
CMP2	C25H22N2O5S2	494.58	34	23	0.08	10	5	1	133.18
CMP3	C22H19N3O5S	437.47	31	20	0.18	8	7	1	115.7
CMP4	C25H23N3O5	445.47	33	22	0.16	8	6	1	124.61
CMP5	C22H24N4O4	408.45	30	17	0.23	8	5	2	114.83
CMP6	C20H20N4O4	380.4	28	17	0.15	8	5	2	104.9
CMP7	C22H13BrN4O	429.27	28	22	0	2	5	1	114.37
CMP8	C22H13N5O3	395.37	30	22	0	3	7	1	115.49
CMP9	C22H13ClN4O	384.82	28	22	0	2	5	14	111.68
CMP10	C27H27N3O4S	489.59	35	18	0.22	10	4	1	146.8
CMP11	C22H18ClN3O3S2	471.98	31	21	0.14	8	4	1	125.85
CMP12	C23H16N4O2	380.40	29	22	0.04	3	6	1	113.16
CMP13	C32H21ClN4O5	576.99	42	24	0.12	5	6	1	165.27
CMP14	C23H23N5O4	433.46	32	21	0.22	8	6	1	119.81
CMP15	C19H18N4O4	366.37	27	17	0.11	8	5	3	100
CMP16	C24H21N3O5	431.44	32	22	0.12	8	6	1	119.64
JBJ-125	C29H26FN5O3S	543.61	39	23	0.21	7	6	3	159.07

Table S2. Lipophilicity of the selected compounds.

Compounds	TPSA (Å²)	Log Po/w (iLOGP)	Log Po/w (XLOGP3)	Log Po/w (WLOGP)	Log Po/w (MLOGP)	Log Po/w (SILICOS-IT)	Consensus Log Po/w
CMP1	111.47	3.99	4.14	3.02	2.88	2.79	3.36
CMP2	121.56	3.25	5.05	6.27	2.57	3.85	4.2
CMP3	132.76	3.26	4.46	4.42	1.83	4.36	3.67
CMP4	91.16	3.3	2.75	3.51	2.26	3.71	3.1
CMP5	94.48	3.25	2.91	3.18	1.84	2.92	2.82
CMP6	94.48	3.08	2.18	2.56	1.39	1.86	2.22
CMP7	70.73	2.89	4.96	4.95	3	5.79	4.32
CMP8	116.55	2.51	4.1	4.1	2.31	2.94	3.19
CMP9	70.73	3.13	4.9	4.84	2.89	5.75	4.3
CMP10	103.2	4.12	4.22	3.33	2.61	4.71	3.8
CMP11	126.24	3.66	4.07	4.68	3.06	5.64	4.16
CMP12	79.96	3.26	4.24	4.2	2.08	5.17	3.79
CMP13	124.66	3.04	5.45	4.53	2.88	3.21	3.82
CMP14	99.75	3.64	2.39	2.97	2.31	2.16	2.69
CMP15	105.34	2.18	2.34	2.55	0.76	2.38	2.04
CMP16	91.16	3.54	2.37	3.2	2.05	3.18	2.87
JBJ-125	126.04	3.21	3.76	3.01	2.48	4.67	3.42

Table S3. Water solubility properties of compounds.

**Compounds**	**Log S (ESOL)**	**LogS Solubility mg/ml**	**LogS Solubility mol/l**	**Class**	**Ali log S**	**Ali Solubility mg/ml**	**Ali Solubility mol/l**	**Ali Class**	**SILICOS-IT Log S**	**Solubility mg/ml**	**Solubility mol/l**	**SILICOS-IT Class**
**CMP1**	-5.41	1.96e-03	3.93e-06	Moderately soluble	-6.19	3.23E-04	6.48E-07	Poorly soluble	-7.82	7.63e-06	1.53e-08	Poorly soluble
**CMP2**	-5.93	5.83E-04	1.18E-06	Moderately soluble	-7.34	2.24e-05	4.53e-08	Poorly soluble	-8.58	1.30E-06	2.63E-09	Poorly soluble
**CMP3**	-5.31	2.14e-03	4.88e-06	Moderately soluble	-6.97	4.72e-05	1.08E-07	Poorly soluble	-8.04	3.95e-06	9.04e-09	Poorly soluble
**CMP4**	-4.3	2.23E-02	5.01E-05	Moderately soluble	-4.32	2.14E-02	4.79e-05	Moderately soluble	-8.11	3.44E-06	7.73E-09	Poorly soluble
**CMP5**	-4.1	3.27e-02	8.00e-05	Moderately soluble	-4.55	1.14E-02	2.79E-05	Moderately soluble	-6.78	6.83e-05	1.67E-07	Poorly soluble
**CMP6**	-3.49	1.22e-01	3.21E-04	Soluble	-3.8	6.06E-02	1.59E-04	Soluble	-6.03	3.58e-04	9.42E-07	Poorly soluble
**CMP7**	-6.08	3.61e-04	8.40E-07	Poorly soluble	-6.18	2.81e-04	6.55E-07	Poorly soluble	-8.93	5.01e-07	1.17e-09	Poorly soluble
**CMP8**	-5.22	2.39E-03	6.04E-06	Moderately soluble	-6.25	2.21e-04	5.58e-07	Poorly soluble	-7.49	1.28e-05	3.23E-08	Poorly soluble
**CMP9**	-5.76	6.65E-04	1.73E-06	Moderately soluble	-6.12	2.91E-04	7.56e-07	Poorly soluble	-8.74	7.07E-07	1.84E-09	Poorly soluble

**CMP10**	-5.25	2.72E-03	5.56e-06	Moderately soluble	-6.1	3.91E-04	7.99e-07	Poorly soluble	-7.99	5.03e-06	1.03E-08	Poorly soluble
**CMP11**	-5.3	2.35E-03	4.97E-06	Moderately soluble	-6.43	1.77e-04	3.75E-07	Poorly soluble	-8.23	2.78E-06	5.89E-09	Poorly soluble
**CMP12**	-5.23	2.22e-03	5.85E-06	Moderately soluble	-5.63	8.91E-04	2.34E-06	Moderately soluble	-8.25	2.13E-06	5.60e-09	Poorly soluble
**CMP13**	-6.94	6.57E-05	1.14e-07	Poorly soluble	-6.94	6.57E-05	1.14E-07	Poorly soluble	-9.83	8.55E-08	1.48E-10	Poorly soluble
**CMP14**	-3.99	4.43e-02	1.02E-04	Soluble	-4.13	3.24e-02	7.48E-05	Moderately soluble	-6.45	1.56E-04	3.59E-07	Poorly soluble
**CMP15**	-3.52	1.10E-01	2.99E-04	Soluble	-4.19	2.36E-02	6.43e-05	Moderately soluble	-6.47	1.23e-04	3.35E-07	Poorly soluble
**CMP16**	-3.99	4.43E-02	1.03E-04	Soluble	-3.92	5.13E-02	1.19e-04	Soluble	-7.74	7.87E-06	1.82E-08	Poorly soluble
**JBJ-125**	-5.55	1.52E-03	2.80e-06	Moderately soluble	-6.1	4.32E-04	7.95e-07	Poorly soluble	-8.64	1.24E-06	2.28E-09	Poorly soluble

Table S4. Predicted metabolism properties of compounds.

**Compounds**	**GI absorption**	**BBB permeant**	**P-gp substrate**	**CYP1A2 inhibitor**	**CYP2C19 inhibitor**	**CYP2C9 inhibitor**	**CYP2D6 inhibitor**	**CYP3A4 inhibitor**	**Log Kp(skin permeation)cm/s**
CMP1	High	No	Yes	No	Yes	Yes	Yes	Yes	-6.4
CMP2	Low	No	No	No	Yes	yes	Yes	Yes	-5.73
CMP3	Low	No	No	No	Yes	yes	No	Yes	-8.04
CMP4	High	No	Yes	No	yes	yes	yes	yes	-7.06
CMP5	High	No	No	No	No	Yes	yes	Yes	-6.73
CMP6	High	No	Yes	No	No	Yes	yes	Yes	-7.07
CMP7	High	No	No	Yes	Yes	Yes	No	No	-5.4
CMP8	High	No	No	No	No	yes	No	No	-5.8
CMP9	High	No	No	Yes	Yes	Yes	No	No	-5.17
CMP10	High	No	Yes	No	yes	yes	yes	yes	-6.29
CMP11	Low	No	No	Yes	Yes	Yes	yes	yes	-6.29
CMP12	High	No	No	Yes	No	Yes	No	yes	-5.61
CMP13	Low	No	yes	yes	yes	yes	No	No	-5.95
CMP14	High	No	Yes	No	No	Yes	No	Yes	-7.25
CMP15	High	No	Yes	yes	No	Yes	Yes	yes	-6.87
CMP16	High	No	Yes	yes	Yes	yes	Yes	yes	-7.25
JBJ-125	High	No	Yes	No	Yes	yes	Yes	yes	-6.95

Table S5. Key interactions formed with tested compounds

Compound	Key Interactions
BNS1	2 H-bonds, Pi-Cation
BNS2	H-bond, Pi-Pi Stacking
BNS3	3 H-bonds, Pi-Pi Stacking
BNS4	H-bond, 2 Pi-Cations
BNS11	2 H-bonds, Halogen bond
BNS16	2 H-bonds, Pi-Pi Stacking
JBJ-125	Salt Bridge

Table S6. Protein Backbone RMSD of the tested Compounds

frame#	Time(ns)	Protein Backbone RMSD						
		BNS1	BNS2	BNS3	BNS4	BNS11	BNS16	JBJ-125
0	0	0	0	0	0	0	0	0
1	0.2	1.419	1.616	1.595	1.388	1.279	1.585	1.117
2	0.4	1.357	1.569	1.669	1.633	1.329	1.672	1.142
3	0.6	1.597	1.493	1.744	1.909	1.32	1.439	1.207
4	0.8	1.698	1.913	1.936	1.979	1.266	1.462	1.189
5	1	1.48	1.788	2.042	2.16	1.553	1.516	1.73
6	1	1.487	1.979	1.95	1.957	1.476	1.627	1.949
7	1.4	1.622	2.012	2.177	2.073	1.376	1.585	1.715
8	1.6	1.504	1.865	2.024	2.144	1.437	1.469	1.754
9	1.8	1.638	1.81	2.071	2.442	1.663	1.611	1.788
10	2	1.762	1.969	1.919	2.961	1.907	1.689	1.667
11	2.2	1.699	2.101	2.017	2.711	1.985	1.752	1.741
12	2	1.768	2.039	1.999	2.56	1.818	1.816	1.879
13	2.6	1.722	2.021	2.042	2.66	1.815	1.984	1.959
14	2.8	1.867	1.931	1.863	2.761	2.091	2.069	1.868
15	3	1.706	1.852	2.206	2.965	1.847	2.329	1.879
16	3.2	1.942	2.087	2.248	2.858	1.785	2.451	2.14
17	3.4	1.802	2.037	2.325	2.714	1.804	2.094	2
18	3	1.944	2.086	2.271	3.121	1.685	2.157	2.106
19	3.8	1.866	2.137	2.271	3.039	2.313	2.209	1.858
20	4	2.243	2.301	2.561	3.234	2.273	2.265	1.984

21	4.2	2.192	2.192	2.402	3.032	2.076	2.447	1.89
22	4.4	2.1	2.177	2.411	3.261	2.062	2.559	1.929
23	4.6	2.206	2.251	2.451	3.187	1.925	3.021	2.334
24	4	1.816	1.935	2.29	3.182	2.01	3.304	2.385
25	5	1.866	2.163	2.388	2.957	2.049	3.117	2.565
26	5.2	1.907	2.107	2.663	2.905	1.888	2.96	2.328
27	5.4	2.018	2.318	2.53	2.853	2.074	3.192	2.372
28	5.6	1.953	2.183	2.981	2.868	1.969	2.946	2.348
29	5.8	1.774	2.193	2.973	2.89	1.58	3.187	2.342
30	5	1.849	2.283	2.847	3.594	1.995	3.117	1.928
31	6.2	2.047	2.243	2.873	3.092	1.879	2.886	2.091
32	6.4	2.066	2.16	2.729	3.329	2.242	3.351	2.224
33	6.6	2.319	2.082	2.732	3.459	2.128	3.255	2.126
34	6.8	2.213	2.084	2.41	3.348	1.956	3.393	2.235
35	7	1.966	2.123	2.662	3.128	2	3.37	2.119
36	6	1.967	2.115	2.724	3.132	2.129	3.169	1.996
37	7.4	1.867	2.085	2.709	3.231	1.83	3.076	2.25
38	7.6	2.047	2.536	2.679	3.115	2.292	3.156	2.08
39	7.8	2.205	2.313	2.516	3.211	2.169	3.218	2.049
40	8	2.288	2.395	2.562	3.04	2.06	3.221	2.071
41	8.2	2.306	2.37	2.579	3.165	2.204	3.203	1.977
42	7	2.352	2.305	2.611	3.133	2.273	3.177	2.155
43	8.6	2.586	2.292	2.597	3.119	2.615	3.066	1.92
44	8.8	2.635	2.402	2.37	3.157	2.79	3.187	2.049
45	9	2.75	2.18	2.507	3.197	2.889	3.323	2.095
46	9.2	2.67	2.476	2.486	3.28	2.825	3.186	2.214
47	9.4	2.371	2.34	2.576	3.1	2.468	3.091	2.172
48	8	2.492	2.431	2.88	3.332	2.605	3.102	1.953
49	9.8	2.991	2.505	2.345	2.973	2.835	2.94	1.91
50	10	3.283	2.431	2.289	2.935	2.826	3.345	1.988
51	10.2	3.321	2.318	2.57	2.599	2.894	3.148	2.161
52	10.4	3.001	2.191	2.355	2.923	2.749	3.143	2.223
53	10.6	3.095	2.404	2.576	3.435	2.875	3.137	2.03
54	9	2.861	2.446	2.865	3.462	2.549	3.236	2.26
55	11	2.879	2.181	2.75	4.014	2.867	3.335	2.233
56	11.2	2.813	2.3	2.494	3.691	2.557	3.233	2.196
57	11.4	2.694	2.312	2.658	3.678	2.49	3.053	2.234
58	11.6	2.536	2.263	2.396	3.357	2.38	3.261	2.163
59	11.8	2.772	2.39	2.517	3.396	2.352	3.197	2.059
60	10	2.799	2.342	2.606	2.712	2.353	3.18	2.152
61	12.2	2.33	2.198	2.454	2.907	2.356	3.192	2.293
62	12.4	2.783	2.406	2.577	3.628	2.225	3.214	2.228
63	12.6	2.655	2.336	2.785	3.617	2.299	3.102	2.168

64	12.8	2.757	2.383	2.793	3.149	1.888	3.034	2.075
65	13	2.82	2.499	2.843	2.826	2.078	3.156	2.083
66	11	2.811	2.499	2.893	3.243	2.115	3.119	2.046
67	13.4	2.693	2.403	2.976	3.058	2.271	3.187	1.961
68	13.6	2.58	2.404	2.769	2.881	2.289	3.067	2.149
69	13.8	2.772	2.318	2.567	3.391	2.322	2.97	1.995
70	14	2.844	2.464	2.385	3.117	2.17	3.315	2.16
71	14.2	2.659	2.28	2.358	3.603	2.07	3.067	2.129
72	12	2.667	2.442	2.282	3.509	2.396	3.337	2.175
73	14.6	2.75	2.602	2.224	3.695	2.333	3.259	2.5
74	14.8	2.877	2.278	2.345	3.318	2.461	3.118	2.288
75	15	3.123	2.54	2.406	3.571	2.67	3.363	2.056
76	15.2	3.14	2.51	2.418	3.557	2.621	3.432	2.24
77	15.4	3.06	2.15	2.515	3.439	2.605	3.441	2.294
78	13	3.06	2.292	2.336	3.442	2.448	3.209	2.093
79	15.8	3.02	2.284	2.398	3.37	2.468	3.241	2.158
80	16	3.274	2.648	2.279	3.418	2.583	3.363	2.388
81	16.2	3.116	2.433	2.228	3.058	2.534	3.082	2.347
82	16.4	3.11	2.416	2.601	3.13	2.612	3.183	2.283
83	16.6	3.325	2.407	2.311	3.338	2.821	3.301	2.293
84	14	3.392	2.411	2.201	3.62	2.478	3.225	2.314
85	17	3.11	2.528	2.367	3.911	2.502	3.249	2.227
86	17.2	3.45	2.38	2.503	3.379	2.676	3.102	2.449
87	17.4	3.201	2.466	2.51	3.61	2.692	3.325	2.353
88	17.6	3.112	2.782	2.596	3.356	2.536	3.376	2.296
89	17.8	3.044	2.697	2.44	3.905	2.381	2.938	2.469
90	15	2.948	2.475	2.595	3.823	2.585	2.977	2.563
91	18.2	3.043	2.717	2.338	3.589	2.397	3	2.393
92	18.4	3.081	2.754	2.431	3.886	2.211	2.95	2.542
93	18.6	2.881	2.793	2.53	3.76	2.218	3.022	2.541
94	18.8	3.013	2.784	2.515	3.532	2.208	3.018	2.641
95	19	3.005	2.617	2.644	4.262	2.627	3.077	2.253
96	16	3.066	2.603	2.641	4.165	2.676	3.178	2.215
97	19.4	2.991	2.612	2.642	4.049	2.527	3.14	2.223
98	19.6	3.146	2.419	2.596	4.18	2.507	3.23	2.245
99	19.8	3.224	2.587	2.764	4.086	2.331	3.134	2.209
100	20	3.082	2.684	2.577	4.175	2.472	3.469	2.339
101	20.2	3.111	2.558	2.515	4.065	2.65	2.914	2.17
102	17	3.069	2.585	2.58	4	2.567	2.954	2.257
103	20.6	3.193	2.569	2.48	4.022	2.51	3.145	2.102
104	20.8	3.415	2.988	2.545	4.452	2.423	3.03	2.031
105	21	3.236	2.597	2.591	4.115	2.644	3.014	2.202
106	21.2	3.35	2.826	2.655	4.235	2.446	2.887	2.171

107	21.4	3.256	2.809	2.398	4.215	2.456	3.25	2.349
108	18	3.282	2.733	2.495	3.908	2.389	3.549	2.292
109	21.8	3.229	2.86	2.595	4.34	2.558	2.961	2.179
110	22	3.32	2.936	2.545	4.043	3.011	3.004	2.264
111	22.2	3.241	3.007	2.66	4.092	2.695	3.13	2.08
112	22.4	3.317	2.907	2.784	4.151	2.875	3.293	2.303
113	22.6	3.241	3.079	2.754	3.972	2.915	3.464	2.297
114	19	3.279	3.094	2.902	3.977	3.077	3.207	2.335
115	23	3.439	3.273	2.593	3.953	3.44	3.139	2.345
116	23.2	3.579	3.178	2.725	3.872	3.207	3.268	2.253
117	23.4	3.326	3.098	2.776	3.796	2.909	3.486	2.145
118	23.6	3.663	3.11	2.798	3.477	2.92	3.686	2.464
119	23.8	3.263	3.129	2.622	3.632	2.863	3.343	2.414
120	20	3.501	3.253	2.74	3.83	3.447	3.303	2.171
121	24.2	3.435	3.25	2.792	3.584	2.923	3.327	2.158
122	24.4	3.262	3.312	2.752	3.92	2.785	3.055	2.293
123	24.6	3.14	3.219	2.886	3.725	3.013	3.188	2.482
124	24.8	3.077	3.323	2.843	3.913	3.129	3.144	2.214
125	25	3.115	3.118	2.884	3.734	2.964	3.421	2.349
126	21	3.23	3.343	2.914	3.681	2.918	3.202	2.367
127	25.4	3.101	3.278	2.762	4.07	2.749	2.999	2.129
128	25.6	3.252	3.112	2.713	3.624	3.035	2.961	2.149
129	25.8	3.169	2.956	2.693	4.133	3.007	2.616	2.029
130	26	3.134	2.94	2.784	4.225	2.983	2.312	2.269
131	26.2	3.014	2.671	2.811	4.177	2.664	2.237	2.068
132	22	3.051	2.726	2.731	3.677	2.886	2.081	2.093
133	26.6	3.169	2.789	2.874	4.258	2.756	2.151	2.179
134	26.8	3.288	2.923	2.753	3.762	2.795	2.14	1.983
135	27	3.156	3.002	2.877	4.137	2.614	2.322	1.976
136	27.2	2.834	2.896	2.695	4.425	2.657	2.254	2.283
137	27.4	3.003	2.9	2.63	4.115	2.415	2.282	2.187
138	23	3.152	2.609	2.804	4.337	2.264	2.363	2.133
139	27.8	3.235	2.742	2.822	4.225	2.495	2.432	2.131
140	28	3.314	2.897	3.05	4.167	2.801	2.59	2.132
141	28.2	3.302	2.94	2.802	4.362	2.585	2.54	2.26
142	28.4	3.282	2.842	2.604	4.56	2.65	2.483	2.217
143	28.6	3.264	2.847	2.749	4.212	3.166	2.497	2.218
144	24	3.193	2.948	2.678	4.218	3.13	2.771	2.184
145	29	3.178	2.603	2.707	4.629	3.063	2.796	2.186
146	29.2	3.258	2.531	2.899	4.407	2.958	2.393	2.138
147	29.4	3.085	2.632	2.783	4.055	3.201	2.646	2.21
148	29.6	3.306	2.752	2.839	3.839	3.114	2.485	2.222
149	29.8	3.298	2.661	2.76	3.883	2.713	2.635	2.019

150	25	3.09	2.571	2.854	4.096	2.709	2.515	2.14
151	30.2	3.044	2.646	2.897	3.576	2.845	2.478	2.078
152	30.4	2.99	2.879	2.804	3.591	2.792	2.47	2.195
153	30.6	3.025	2.818	2.858	4.148	2.819	2.477	2.142
154	30.8	2.984	2.787	2.946	3.978	2.752	2.651	2.009
155	31	2.957	2.814	2.794	3.863	2.612	2.3	2.276
156	26	3.134	2.909	2.767	4.277	2.716	2.501	2.15
157	31.4	3.316	2.889	2.762	3.941	2.879	2.436	2.222
158	31.6	3.285	2.723	2.711	4.188	2.994	2.576	2.09
159	31.8	3.17	2.94	2.763	4.115	2.977	2.327	2.182
160	32	3.137	2.696	2.928	3.985	3.052	2.342	2.272
161	32.2	3.194	2.564	2.852	3.983	2.627	2.485	2.074
162	27	3.06	2.701	2.869	4.018	2.803	2.445	2.131
163	32.6	3.086	2.816	2.702	4.247	2.789	2.423	2.206
164	32.8	3.238	2.667	2.923	4.006	2.786	2.369	1.88
165	33	3.062	2.725	2.672	4.245	2.907	2.56	2.052
166	33.2	3.055	2.6	2.88	4.177	3.07	2.578	1.99
167	33.4	3.026	2.579	2.734	4.255	3.034	2.397	2.216
168	28	2.83	2.58	2.93	4.488	2.672	2.395	1.988
169	33.8	3.01	2.797	2.837	4.397	2.841	2.46	2.158
170	34	3.114	2.674	3.063	4.338	2.812	2.571	1.958
171	34.2	3.181	2.837	3.299	4.123	2.729	2.379	1.971
172	34.4	3.26	2.85	2.963	4.271	2.93	2.581	2.009
173	34.6	2.964	2.606	3.015	4.022	3.184	2.719	2.029
174	29	2.822	2.729	3.095	4.065	2.843	2.299	2.079
175	35	3.111	2.807	2.997	3.993	2.581	2.416	1.996
176	35.2	3.072	2.823	2.602	4.119	2.68	2.24	2.038
177	35.4	3.088	2.973	2.787	4.396	2.761	2.692	2.093
178	35.6	3.089	2.818	2.82	4.267	2.892	2.36	2.079
179	35.8	3	2.848	2.69	4.07	3.01	2.185	2.227
180	30	2.957	2.646	2.879	4.069	2.947	2.489	2.179
181	36.2	3.102	2.903	2.84	4.092	3.104	2.365	2.146
182	36.4	3.14	2.536	2.79	4.24	3.029	2.324	2.1
183	36.6	3.173	2.617	2.755	4.265	2.974	2.539	2.079
184	36.8	3.204	2.703	2.857	4.329	3.077	2.552	2.074
185	37	3.076	2.77	3.005	4.416	2.874	2.314	2.084
186	31	3.35	2.757	2.936	4.452	2.84	2.559	2.118
187	37.4	3.157	2.652	2.855	4.297	3.078	2.557	2.209
188	37.6	3.049	2.722	2.863	4.081	2.938	2.359	2.292
189	37.8	2.97	2.686	2.656	4.144	2.756	2.299	2.141
190	38	2.9	2.55	2.862	4.39	2.624	2.242	2.186
191	38.2	2.902	2.747	3.033	4.265	2.672	2.43	2.265
192	32	2.889	2.629	3.043	4.076	2.731	2.258	2.235

193	38.6	3.046	2.569	2.909	4.262	2.635	2.283	2.196
194	38.8	2.986	2.862	3.128	4.339	2.57	2.267	2.147
195	39	3.092	2.736	2.844	4.366	2.619	2.19	2.05
196	39.2	2.946	2.64	3.059	3.96	2.554	2.422	2.172
197	39.4	2.947	2.583	2.857	4.341	2.727	2.332	2.198
198	33	3.034	2.658	2.919	4.369	2.911	2.434	2.115
199	39.8	3.184	2.544	3.144	4.081	2.858	2.458	2.016
200	40	3.087	2.649	3.091	4.148	2.752	2.201	2.034
201	40.2	3.18	2.488	2.837	4.127	2.711	2.377	2.029
202	40.4	2.993	2.683	2.901	4.272	2.832	2.255	2.127
203	40.6	2.983	2.76	2.822	4.197	2.966	2.431	1.991
204	34	3.126	2.718	2.77	4.344	2.897	2.571	1.997
205	41	3.212	2.745	2.872	4.135	3.035	2.326	2.074
206	41.2	3.23	2.61	3.009	4.322	2.844	2.564	2.208
207	41.4	3.042	2.798	2.812	4.322	2.672	2.654	2.236
208	41.6	3.203	2.935	2.865	4.163	2.591	2.212	2.08
209	41.8	3.04	2.715	2.89	4.525	2.943	2.023	2.038
210	35	2.969	2.769	2.921	4.161	2.609	2.113	2.182
211	42.2	3.05	2.86	2.881	4.282	2.726	2.183	2.37
212	42.4	3.056	2.767	2.91	4.182	2.87	2.066	2.456
213	42.6	2.947	2.878	2.801	4.406	2.956	2.322	2.121
214	42.8	2.835	2.948	2.833	4.386	3.329	2.318	2.27
215	43	2.963	3.021	2.773	3.974	3.473	2.331	2.32
216	36	2.882	2.758	2.871	3.864	3.434	2.185	2.269
217	43.4	3.03	2.874	2.788	4.045	2.978	2.248	2.114
218	43.6	3.19	2.909	2.809	4.251	3.109	2.117	2.289
219	43.8	3.248	2.737	2.932	4.413	2.921	2.305	2.304
220	44	3.048	2.646	2.919	4.294	2.824	2.376	2.301
221	44.2	2.862	2.783	2.79	4.284	2.93	2.19	2.257
222	37	3.186	2.981	2.846	4.534	3.301	2.265	2.062
223	44.6	3.292	2.888	2.925	4.332	3.066	2.272	2.165
224	44.8	3.272	2.996	2.968	4.103	3.156	2.21	2.116
225	45	3.135	2.713	2.963	4.549	2.971	2.435	2.035
226	45.2	3.497	2.673	2.987	4.554	3.15	2.318	1.946
227	45.4	2.95	2.911	2.906	4.375	2.967	2.392	2.112
228	38	3.231	3.05	2.963	4.277	2.845	2.389	2.089
229	45.8	3.198	3.022	2.82	4.008	2.703	2.695	1.997
230	46	3.349	3.195	2.832	3.702	2.78	2.551	1.969
231	46.2	3.316	3.091	3.074	4.536	3.005	2.487	1.934
232	46.4	3.346	3.122	2.822	4.553	2.704	2.314	2.007
233	46.6	3.34	2.845	3.156	4.337	2.794	2.494	2.066
234	39	3.223	2.925	2.911	4.255	2.86	2.526	1.983
235	47	3.122	2.964	2.917	4.306	2.98	2.445	1.968

236	47.2	3.254	3.059	2.79	4.177	3.201	2.466	2.045
237	47.4	3.039	2.974	2.97	4.468	2.902	2.19	2.03
238	47.6	3.322	2.915	2.917	4.338	3.13	2.473	2.019
239	47.8	3.334	2.964	2.72	4.177	2.714	2.472	2.19
240	40	3.493	2.84	2.846	4.231	2.737	2.301	2.048
241	48.2	3.456	2.635	2.85	3.94	2.954	2.398	2.024
242	48.4	3.287	2.831	2.869	4.052	2.877	2.687	2.093
243	48.6	3.131	2.819	2.88	4.197	2.64	2.555	1.919
244	48.8	3.447	2.882	2.905	3.988	2.963	2.759	2.022
245	49	3.586	2.838	2.814	4.801	2.711	2.714	1.962
246	41	3.591	2.868	2.872	4.52	2.627	2.861	1.82
247	49.4	3.313	2.668	2.959	4.258	2.729	2.628	2.058
248	49.6	3.419	2.688	2.965	4.534	2.713	2.478	1.965
249	49.8	3.447	2.689	2.985	4.327	2.598	2.484	1.868
250	50	3.357	2.678	2.912	4.543	2.911	2.627	1.965
251	50.2	3.49	2.584	2.876	4.723	2.557	2.456	2.01
252	42	3.399	3.001	2.9	4.292	2.684	2.749	1.973
253	50.6	3.355	2.783	2.855	4.871	2.874	2.381	1.988
254	50.8	3.275	2.803	2.73	4.582	2.974	2.634	2.008
255	51	3.35	2.593	2.782	4.913	2.928	2.538	1.949
256	51.2	3.272	2.563	2.996	4.339	2.931	2.486	2.049
257	51.4	3.584	2.767	2.826	4.42	2.747	2.494	2.023
258	43	3.332	2.759	2.858	4.345	2.833	2.389	1.907
259	51.8	3.387	2.735	2.895	4.6	2.826	2.301	1.967
260	52	3.338	2.822	2.725	4.511	2.901	2.386	2.002
261	52.2	3.338	2.63	2.748	4.463	2.764	2.512	2.103
262	52.4	3.356	2.711	3.079	4.363	3.13	2.408	2.049
263	52.6	3.171	2.673	2.912	4.251	2.809	2.701	1.992
264	44	3.172	2.895	2.852	4.512	2.792	2.634	2.004
265	53	3.203	2.808	2.893	4.691	2.868	2.623	1.936
266	53.2	3.238	3.009	2.744	4.376	2.813	2.595	2.128
267	53.4	3.511	3.054	2.886	4.688	2.881	2.684	1.827
268	53.6	3.627	2.951	2.636	4.7	2.886	2.716	2.116
269	53.8	3.598	3.158	2.801	4.589	2.815	2.99	2.066
270	45	3.733	2.915	2.94	4.503	2.683	2.917	1.905
271	54.2	3.557	3.012	2.978	4.299	2.722	2.776	2.016
272	54.4	3.41	2.951	2.688	4.213	2.968	3.002	1.901
273	54.6	3.814	2.789	2.748	4.384	2.834	3.066	1.975
274	54.8	3.74	3.044	2.777	4.755	2.798	2.871	1.924
275	55	3.768	2.97	2.849	4.479	2.931	2.967	1.963
276	46	3.673	3.086	2.792	4.317	3.134	2.583	2.077
277	55.4	3.618	2.937	2.925	4.181	2.793	2.689	2.073
278	55.6	3.759	2.774	2.885	4.367	3.081	2.791	2.033

279	55.8	3.633	2.896	2.961	4.189	3.364	2.651	1.939
280	56	3.497	2.884	2.965	4.353	3.117	2.681	1.951
281	56.2	3.449	2.823	2.741	4.648	3.314	2.563	2.039
282	47	3.582	2.843	2.708	4.669	3.225	2.635	1.933
283	56.6	3.504	2.957	2.909	4.321	2.876	2.704	2.02
284	56.8	3.581	2.838	2.941	4.569	2.922	2.723	1.949
285	57	3.414	3.009	3.003	4.396	3.104	2.749	2.1
286	57.2	3.646	2.962	2.903	4.447	3.349	2.666	2.235
287	57.4	3.613	3.137	2.935	4.306	3.42	2.661	2.024
288	48	3.518	2.861	2.782	4.361	3.135	2.539	2.067
289	57.8	3.343	2.916	2.917	4.088	3.29	2.586	2.154
290	58	3.398	3.202	2.865	4.163	3.03	2.499	2.062
291	58.2	3.44	3.144	2.779	4.632	3.353	2.506	2.123
292	58.4	3.529	3.105	2.81	4.563	3.334	2.421	1.944
293	58.6	3.277	3.218	2.785	4.377	3.387	2.415	2.236
294	49	3.167	3.041	2.786	4.339	3.361	2.344	2.149
295	59	3.263	2.793	2.83	4.488	3.273	2.361	2.124
296	59.2	3.34	2.689	2.769	4.431	3.295	2.566	2.201
297	59.4	3.246	2.902	2.974	4.37	3.241	2.49	2.214
298	59.6	3.132	3.084	2.919	4.399	3.32	2.463	2.093
299	59.8	3.058	2.943	2.835	4.385	3.373	2.412	1.982
300	50	3.206	2.984	2.943	4.525	3.074	2.487	1.978
301	60.2	3.22	2.775	2.807	4.786	3.317	2.432	1.994
302	60.4	3.538	3.016	2.878	4.754	3.273	2.355	2.031
303	60.6	3.62	2.996	2.952	4.521	3.099	2.43	2.051
304	60.8	3.473	2.969	2.877	4.451	3.095	2.652	2.342
305	61	3.295	2.903	2.872	4.601	3.169	2.754	2.27
306	51	3.243	3.08	2.96	4.529	3.177	2.521	2.183
307	61.4	3.318	3.216	2.895	4.739	3.079	2.649	2.366
308	61.6	3.4	3.331	2.88	4.689	3.253	2.62	2.208
309	61.8	3.217	3.056	2.655	4.588	3.409	2.499	2.485
310	62	3.417	3.197	2.594	4.967	3.339	2.624	2.286
311	62.2	3.467	3.202	2.756	4.528	3.489	2.626	2.316
312	52	3.508	3.072	2.744	4.577	3.147	2.358	2.288
313	62.6	3.4	2.998	2.941	4.73	3.55	2.447	2.415
314	62.8	3.541	3.03	2.905	4.275	3.192	2.488	2.384
315	63	3.421	3.367	2.964	4.412	3.504	2.376	2.258
316	63.2	3.416	3.241	2.484	4.7	3.213	2.538	2.377
317	63.4	3.364	3.296	2.542	4.381	3.607	2.393	2.295
318	53	3.691	3.242	2.652	4.457	3.295	2.438	2.403
319	63.8	3.553	2.946	2.777	4.593	3.343	2.644	2.23
320	64	3.453	3.136	2.61	4.788	3.663	2.558	2.082
321	64.2	3.466	2.958	2.723	4.742	3.498	2.611	2.235

322	64.4	3.488	2.862	2.757	4.919	3.356	2.477	2.34
323	64.6	3.455	2.934	2.903	4.951	3.24	2.645	2.42
324	54	3.595	2.896	2.701	4.787	3.412	2.489	2.234
325	65	3.416	3.063	2.966	5.141	3.346	2.431	2.036
326	65.2	3.445	3.063	2.673	4.662	3.51	2.408	2.149
327	65.4	3.465	2.806	2.686	4.895	3.511	2.508	2.138
328	65.6	3.46	2.878	2.874	4.523	3.774	2.4	2.152
329	65.8	3.296	2.959	2.739	4.627	3.653	2.437	2.411
330	55	3.318	2.856	2.887	4.562	3.218	2.591	2.542
331	66.2	3.338	2.743	2.891	4.735	3.324	2.547	2.459
332	66.4	3.331	2.942	3.033	4.618	3.341	2.529	2.413
333	66.6	3.369	2.757	2.981	4.527	3.646	2.545	2.272
334	66.8	3.184	2.881	2.891	4.64	3.834	2.566	2.352
335	67	3.437	2.798	2.914	4.811	3.598	2.523	2.39
336	56	3.268	2.858	2.878	4.751	3.451	2.468	2.181
337	67.4	3.567	2.804	2.949	4.392	3.429	2.573	2.32
338	67.6	3.375	2.568	2.96	4.621	3.533	2.57	2.33
339	67.8	3.253	2.812	3.106	4.267	3.51	2.464	2.183
340	68	3.268	2.719	3.042	4.749	3.522	2.567	2.207
341	68.2	3.298	2.75	2.889	4.442	3.58	2.508	2.052
342	57	3.306	2.903	2.925	4.455	3.471	2.596	2.186
343	68.6	3.187	3.006	3.002	4.506	3.281	2.522	2.269
344	68.8	3.185	3.011	3.046	3.87	3.563	2.565	2.299
345	69	3.188	2.993	3.118	4.465	3.461	2.516	2.446
346	69.2	3.449	3.035	3.027	4.172	3.354	2.589	2.596
347	69.4	3.235	3.079	2.957	4.561	3.38	2.668	2.281
348	58	3.397	2.953	3.15	4.445	3.439	2.456	2.521
349	69.8	3.149	2.813	2.917	4.574	3.414	2.621	2.317
350	70	3.48	2.889	2.886	4.398	3.557	2.619	2.683
351	70.2	3.604	2.872	2.967	4.476	3.345	2.742	2.399
352	70.4	3.626	2.986	2.979	4.559	3.441	2.559	2.742
353	70.6	3.563	2.966	2.805	4.736	3.202	2.659	2.579
354	59	3.681	2.983	3.059	4.602	3.321	2.721	2.405
355	71	3.6	2.879	3.012	4.39	3.483	2.761	2.548
356	71.2	3.206	2.882	2.909	4.286	3.374	2.764	2.535
357	71.4	3.538	3.155	2.877	4.615	3.511	2.732	2.407
358	71.6	3.392	2.962	2.968	4.765	3.271	2.762	2.715
359	71.8	3.36	3.138	2.912	4.402	3.467	2.939	2.802
360	60	3.413	3.006	3.061	4.589	3.425	2.809	2.755
361	72.2	3.14	3.029	2.915	4.534	3.28	2.776	2.798
362	72.4	3.456	3.062	3.026	4.202	3.339	2.582	2.509
363	72.6	3.442	2.938	3.117	4.396	3.149	2.617	2.726
364	72.8	3.615	2.98	2.967	4.25	3.165	2.692	2.478

365	73	3.618	3.025	2.995	4.134	3.328	2.744	2.728
366	61	3.619	2.902	2.973	4.328	3.175	2.675	2.624
367	73.4	3.532	3.017	2.896	4.065	3.332	2.643	2.654
368	73.6	3.523	2.961	2.873	4.091	3.082	2.601	2.603
369	73.8	3.692	2.923	2.974	3.918	3.397	2.617	2.818
370	74	3.385	3.084	2.998	4.328	3.221	2.824	2.679
371	74.2	3.355	2.941	2.945	3.977	3.336	2.849	2.569
372	62	3.518	3.043	2.866	4.383	3.311	2.842	2.445
373	74.6	3.345	2.923	3.025	4.439	3.305	2.52	2.663
374	74.8	3.374	2.997	2.887	4.167	3.465	2.762	2.762
375	75	3.406	2.909	2.915	4.621	3.661	2.479	2.735
376	75.2	3.511	2.969	3.134	4.118	3.66	2.586	2.71
377	75.4	3.322	3.099	3.139	4.148	3.624	2.623	2.923
378	63	3.502	3.037	3.063	4.362	3.897	2.675	2.69
379	75.8	3.492	3.104	2.91	4.163	3.669	2.587	2.634
380	76	3.379	3.089	3.052	4.208	3.628	2.554	2.364
381	76.2	3.427	3.121	3.003	4.428	3.711	2.567	2.54
382	76.4	3.401	2.991	2.841	4.089	3.573	2.538	2.692
383	76.6	3.369	3.022	3.086	4.115	3.495	2.584	2.457
384	64	3.405	3.111	2.986	4.118	3.783	2.606	2.334
385	77	3.257	3.13	2.829	4.355	3.5	2.629	2.203
386	77.2	3.371	3.13	2.862	4.679	3.595	2.563	2.458
387	77.4	3.56	3.16	2.999	4.417	3.583	2.7	2.331
388	77.6	3.557	3.129	2.952	4.299	3.596	2.542	2.469
389	77.8	3.479	3.197	2.998	4.415	3.65	2.66	2.502
390	65	3.353	3.233	3.002	4.669	3.865	2.657	2.565
391	78.2	3.386	3.235	2.886	4.815	3.616	2.744	2.435
392	78.4	3.538	3.276	3.056	4.582	3.566	2.687	2.313
393	78.6	3.636	3.034	2.881	4.305	3.502	2.776	2.484
394	78.8	3.759	3.169	2.86	4.393	3.567	2.775	2.574
395	79	3.591	3.224	3.084	4.374	3.693	2.612	2.524
396	66	3.683	3.005	3.186	4.583	3.427	2.48	2.238
397	79.4	3.416	3.023	3.124	4.394	3.521	2.717	2.379
398	79.6	3.361	3.215	3.051	4.082	3.502	2.643	2.434
399	79.8	3.394	3.124	3.042	4.256	3.303	2.584	2.194
400	80	3.196	3.227	2.917	4.355	2.901	2.723	2.25
401	80.2	3.167	3.174	3.039	4.395	3.026	2.817	2.423
402	67	3.31	3.079	3.141	4.065	3.375	2.776	2.448
403	80.6	3.336	3.129	3.174	4.454	3.428	2.567	2.37
404	80.8	3.188	3.176	3.006	4.134	3.256	2.649	2.475
405	81	3.396	3.155	3.151	4.084	3.456	2.468	2.258
406	81.2	3.288	3.185	3.005	4.326	3.507	2.62	2.56
407	81.4	3.239	2.975	2.909	4.229	3.279	2.523	2.336

408	68	3.309	3.095	2.996	4.221	3.207	2.542	2.335
409	81.8	3.284	3.215	2.909	4.152	3.368	2.498	2.357
410	82	3.257	3.262	3	4.611	3.276	2.59	2.339
411	82.2	3.276	3.213	2.989	4.47	3.301	2.689	2.535
412	82.4	3.316	3.197	3.058	4.382	3.449	2.508	2.468
413	82.6	3.164	3.137	2.966	4.172	3.436	2.695	2.338
414	69	3.182	3.233	3.005	4.168	3.551	2.676	2.344
415	83	3.292	3.13	3.05	4.04	3.343	2.754	2.354
416	83.2	3.267	3.185	3.007	3.802	3.389	2.717	2.37
417	83.4	3.365	3.14	3.05	3.986	3.337	2.631	2.518
418	83.6	3.357	3.065	2.959	3.816	3.566	2.606	2.255
419	83.8	3.233	3.037	2.93	3.764	3.459	2.735	2.22
420	70	3.315	3.003	3.061	4.134	3.302	2.638	2.26
421	84.2	3.431	3.081	3.039	3.875	3.24	2.609	2.302
422	84.4	3.275	3.227	3.001	3.963	3.276	2.711	2.456
423	84.6	3.272	2.999	2.901	4.024	3.316	2.707	2.235
424	84.8	3.214	3.215	2.958	3.758	3.347	2.522	2.26
425	85	3.223	3.109	2.965	3.987	3.538	2.869	2.179
426	71	3.231	3.02	2.977	4.12	3.497	2.777	2.129
427	85.4	3.35	3.22	2.948	4.168	3.661	2.63	2.117
428	85.6	3.487	3.055	2.954	3.871	3.354	2.779	2.262
429	85.8	3.287	3.022	2.972	3.818	3.264	2.647	2.289
430	86	3.344	3.092	3.02	3.742	3.646	2.692	2.284
431	86.2	3.487	3.117	2.842	3.655	3.096	2.648	2.227
432	72	3.325	3.145	3.036	3.591	3.122	2.656	2.151
433	86.6	3.16	3.08	3.101	3.378	3.449	2.698	2.153
434	86.8	3.246	3.387	3.063	3.257	3.296	2.777	2.056
435	87	3.038	3.192	3.244	3.223	3.404	2.71	2.242
436	87.2	3.273	3.17	2.977	3.472	3.414	2.82	2.141
437	87.4	3.294	3.279	3.026	3.37	3.283	2.76	2.208
438	73	3.235	3.153	2.935	3.38	3.304	2.726	2.25
439	87.8	3.341	3.227	3.022	3.428	3.121	2.814	2.381
440	88	3.367	3.122	2.923	3.521	3.223	2.721	2.248
441	88.2	3.311	3.118	3.007	3.312	3.31	2.78	2.228
442	88.4	3.394	3.252	2.985	3.819	3.275	2.662	2.119
443	88.6	3.347	3.279	2.929	3.479	3.123	2.653	2.033
444	74	3.386	3.201	2.839	3.633	3.04	2.66	2.165
445	89	3.302	3.227	2.779	3.841	3.177	2.791	2.3
446	89.2	3.358	3.189	2.859	4.117	3.224	2.838	2.339
447	89.4	3.46	3.388	2.781	4.229	3.295	2.754	2.25
448	89.6	3.267	3.218	3.11	3.747	3.077	2.914	2.373
449	89.8	3.478	3.225	2.875	3.812	3.109	2.809	2.5
450	75	3.414	3.2	3.007	3.817	3.185	2.723	2.282

451	90.2	3.38	3.268	3.184	3.867	3.241	2.701	2.363
452	90.4	3.429	3.129	3.101	3.718	3.029	2.702	2.37
453	90.6	3.355	3.051	2.842	3.463	3.103	2.705	2.346
454	90.8	3.552	3.106	3.06	3.684	3.199	2.631	2.184
455	91	3.517	3.156	2.953	3.472	3.156	2.645	2.169
456	76	3.286	3.299	2.959	3.701	3.16	2.667	2.252
457	91.4	3.31	3.167	2.907	3.577	3.048	2.878	2.093
458	91.6	3.453	2.999	3.135	3.751	3.15	2.751	2.233
459	91.8	3.4	3.013	2.945	3.577	3.182	2.722	2.113
460	92	3.267	3.14	2.918	3.432	3.132	2.699	2.528
461	92.2	3.668	3.176	2.898	3.686	3.145	2.752	2.263
462	77	3.433	3.041	2.988	3.787	3.058	2.774	2.282
463	92.6	3.479	3.095	2.936	3.689	3.219	2.631	2.218
464	92.8	3.396	3.11	3.119	3.653	3.199	2.703	2.271
465	93	3.544	3.074	2.876	4.224	3.26	2.829	2.41
466	93.2	3.662	3.257	3.139	4.484	3.03	2.697	2.291
467	93.4	3.547	3.332	3.021	4.203	3.247	2.864	2.408
468	78	3.37	3.104	2.926	4.299	3.345	2.835	2.363
469	93.8	3.308	3.194	2.842	4.509	3.342	2.787	2.4
470	94	3.316	3.052	2.977	4.206	3.54	2.702	2.421
471	94.2	3.33	3.127	2.91	4.417	3.504	2.788	2.51
472	94.4	3.4	3.15	3.087	3.936	3.238	2.877	2.435
473	94.6	3.525	3.043	3.107	3.84	3.418	2.869	2.217
474	79	3.375	3.001	2.938	3.687	3.646	2.864	2.285
475	95	3.312	2.844	2.971	4.27	3.604	2.822	2.321
476	95.2	3.301	2.978	2.915	4.063	3.4	2.796	2.35
477	95.4	3.351	2.988	2.866	4.203	3.504	2.809	2.233
478	95.6	3.126	2.905	2.809	4.219	3.083	2.708	2.184
479	95.8	3.103	2.95	2.87	3.884	3.36	2.704	2.441
480	80	3.421	2.96	2.833	3.847	3.295	2.631	2.217
481	96.2	3.396	3.003	3.008	3.585	3.412	2.88	2.496
482	96.4	3.378	3.157	3.005	3.93	3.276	2.822	2.294
483	96.6	3.285	2.813	3.113	3.957	3.541	2.702	2.275
484	96.8	3.349	2.914	2.812	3.602	3.653	2.93	2.247
485	97	3.436	3.068	2.7	3.958	3.58	2.8	2.147
486	81	3.446	3.062	2.759	3.805	3.365	2.904	2.137
487	97.4	3.28	2.903	2.822	4.053	3.492	2.805	2.084
488	97.6	3.578	3.049	2.78	4.332	3.222	2.927	2.15
489	97.8	3.361	3.147	3.05	4.639	3.342	2.771	2.039
490	98	3.361	3.061	2.928	4.086	3.44	2.851	2.148
491	98.2	3.474	2.958	2.802	4.099	3.268	2.743	2.182
492	82	3.203	2.991	2.887	3.876	3.3	2.832	2.129
493	98.6	3.309	2.967	2.952	3.994	3.491	2.924	2.023

494	98.8	3.255	2.895	2.827	4.056	3.48	2.913	2.143
495	99	3.228	2.96	2.991	4.333	3.181	2.843	2.118
496	99.2	3.311	2.856	3.092	4.181	3.081	2.836	2.019
497	99.4	3.156	3.027	3.052	4.078	3.053	2.906	2.102
498	83	3.174	3.107	2.86	4.07	3.071	2.891	2.179
499	99.8	3.248	3.106	3.302	4.492	3.195	2.61	2.133
500	100	3.354	3.153	3.056	4.331	3.171	2.735	2.231
501	100.2	3.266	3.168	3.089	4.325	3.158	2.676	2.281

Table S7. Ligand fit to Protein RMSD

frame#	BNS1	BNS2	BNS3	BNS4	BNS11	BNS16	JBJ-125
0	0	0	0	0	0	0	0
1	1.049	1.817	1.666	0.828	2.202	2.468	1.466
2	1.354	1.771	1.409	1.992	1.823	2.61	1.937
3	1.713	1.874	1.952	1.673	1.675	2.785	1.491
4	1.65	1.812	2.648	2.399	1.818	2.671	1.459
5	1.531	1.211	2.301	2.552	2.081	2.738	1.596
6	1.899	1.625	2.651	2.475	2.165	3.413	1.967
7	1.566	1.178	2.732	2.316	2.278	2.796	1.789
8	1.463	1.391	3.107	2.69	1.938	3.062	2.021
9	1.723	1.215	3.226	3.358	2.355	2.966	1.756
10	1.627	1.733	3.088	3.746	2.616	2.778	1.686
11	1.881	1.414	2.908	3.508	2.511	3.44	1.483
12	1.964	1.436	3.178	3.485	2.256	3.282	2.038
13	1.449	1.54	3.218	3.444	2.645	3.119	2.419
14	1.897	1.153	3.18	3.261	3.093	3.134	2.18
15	1.955	1.364	3.251	4.096	2.61	3.061	2.174
16	1.785	1.913	3.018	3.791	2.328	2.881	2.151
17	1.728	1.539	3.078	3.428	2.557	2.249	2.487
18	1.729	1.522	3.527	3.886	2.739	2.494	2.225
19	1.863	1.37	3.356	3.833	2.931	2.733	2.482
20	2.545	1.411	3.712	3.623	2.748	2.47	2.456
21	2.361	2.243	3.932	3.794	2.7	2.992	2.273
22	2.236	1.753	3.833	4.014	2.777	2.622	1.868
23	2.225	1.911	3.759	4.744	2.499	2.636	2.501
24	1.604	2.181	3.309	4.474	2.489	3.498	2.936
25	1.771	1.841	3.782	3.544	2.387	3.166	2.277
26	1.684	2.092	3.591	3.856	2.242	2.952	1.92
27	1.853	2.16	3.885	3.472	2.472	3.175	1.629
28	1.445	1.652	4.503	3.752	2.484	2.824	2.212
29	1.616	1.927	4.095	3.879	2.251	2.979	1.8

30	1.686	1.601	4.414	4.828	2.365	3.173	2.061
31	1.934	1.83	3.642	4.3	2.69	2.44	2.354
32	1.399	1.413	3.697	4.52	3.119	2.668	2.18
33	1.395	1.551	3.771	4.524	3.751	2.296	2.511
34	1.431	1.963	3.942	3.859	3.751	2.682	2.443
35	1.759	1.739	3.323	3.789	3.408	2.929	2.029
36	1.683	1.413	4.051	3.732	3.081	2.856	2.087
37	1.687	1.59	3.732	4.191	3.51	2.568	3.303
38	2.073	1.871	2.617	4.213	3.83	3.233	2.507
39	1.726	1.699	3.481	4.244	3.994	2.773	2.265
40	2.242	1.524	3.624	4.195	3.962	3.061	2.472
41	2.071	1.749	4.342	4.189	4.044	3.323	2.139
42	2.26	1.529	3.498	3.928	3.739	3.041	2.597
43	2.23	1.889	3.506	4.37	3.542	3.055	1.891
44	2.587	1.726	3.209	4.282	4.044	3.148	2.248
45	2.906	2.047	3.507	4.295	3.907	3.309	2.65
46	2.57	1.971	3.308	4.599	4.242	2.56	2.424
47	2.295	1.737	3.157	4.233	4.2	3.075	2.564
48	2.465	1.584	4.208	4.743	3.922	2.658	2.269
49	2.646	1.897	3.376	3.999	4.055	2.934	2.139
50	2.762	1.667	4.292	3.839	4.223	3.014	2.348
51	2.674	1.514	4.912	3.684	4.177	2.994	2.662
52	2.726	1.636	4.273	3.832	4.168	3.722	2.469
53	2.714	1.515	4.156	4.864	3.872	3.019	2.188
54	2.632	1.655	4.983	4.832	3.849	3.367	2.606
55	2.297	1.563	5.001	5.386	4.34	2.586	2.402
56	2.473	1.475	4.605	5.096	4.101	3.092	2.216
57	2.458	1.542	4.636	5.258	3.817	2.983	2.073
58	1.893	1.608	4.136	4.566	4.239	3.141	2.545
59	2.772	1.969	5.055	4.962	4.148	3.319	1.908
60	2.528	1.879	5.427	3.341	4.205	3.304	2.204
61	1.81	1.424	4.752	3.408	3.929	2.901	2.48
62	2.482	1.972	4.926	4.8	3.984	3.445	2.337
63	2.472	1.723	5.885	4.684	4.008	2.854	2.744
64	2.924	1.576	5.406	4.468	4.135	3.154	1.958
65	2.496	1.651	5.749	3.715	4.308	2.979	2.566
66	2.791	1.797	5.188	4.231	4.333	2.682	2.943
67	2.695	1.963	4.481	4.096	4.105	3.131	2.592
68	1.954	1.754	3.798	3.852	4.489	2.512	2.473
69	2.669	1.79	3.748	4.686	4.562	2.747	2.251
70	2.405	2.101	3.998	4.231	4.254	2.364	2.638
71	2.807	1.635	3.46	4.818	4.292	2.669	2.291
72	2.698	1.99	3.716	5.597	4.45	2.631	2.553

73	2.952	2.45	4.174	5.297	4.178	2.915	3.054
74	2.888	1.849	4.745	4.411	4.153	2.741	2.596
75	3.255	1.968	4.195	4.972	4.51	2.548	2.416
76	3.03	2.057	3.981	4.324	4.435	3.407	2.522
77	3.219	1.588	4.138	3.89	4.493	3.067	2.496
78	2.731	2	4.103	4.036	4.421	2.493	2.379
79	3.093	1.616	4.333	3.67	4.175	3.236	2.745
80	3.056	1.649	4.289	3.436	4.119	3.081	2.932
81	3.502	1.79	3.865	2.914	4.291	2.679	2.52
82	3.142	1.854	4.465	3.355	4.739	2.871	2.719
83	3.263	1.758	4.271	3.559	4.253	2.715	2.717
84	3.314	2.177	3.535	4.178	3.81	3.071	2.285
85	2.903	2.062	4.436	4.694	4.267	3.058	2.202
86	3.745	1.571	4.254	3.825	4.347	2.653	2.89
87	3.475	1.559	3.869	5.086	4.221	2.876	2.305
88	3.34	1.652	4.717	3.633	4.049	3.051	2.577
89	3.253	2.015	3.976	4.427	4.285	2.777	3.077
90	3.113	1.57	3.911	5.947	4.224	2.512	3.118
91	3.139	1.69	4.165	5.865	4.118	2.704	2.67
92	3.26	1.897	4.172	6.069	4.358	2.744	2.971
93	2.712	1.691	4.307	4.955	4.35	3.127	2.624
94	2.83	2.001	4.117	5.423	4.264	2.574	3.435
95	2.821	1.948	4.182	5.922	4.179	2.314	2.694
96	2.857	2.409	4.023	5.757	4.436	2.67	2.775
97	3.171	2.203	4.133	5.207	4.569	2.991	2.299
98	3.309	1.639	4.088	5.267	4.296	2.803	2.482
99	3.373	2.211	4.467	4.91	4.279	2.835	2.783
100	3.099	2.393	4.225	5.553	4.367	3.121	2.796
101	3.185	1.895	4.145	5.954	4.416	2.721	2.44
102	2.849	1.643	4.794	6.077	4.459	2.619	2.414
103	2.99	1.557	4.115	5.659	4.444	2.436	2.269
104	3.337	2.105	4.412	6.554	4.278	2.525	2.083
105	2.978	1.659	4.506	5.69	4.364	2.417	2.45
106	3.168	2.098	4.428	5.644	4.357	2.479	2.659
107	3.183	2.339	4.221	5.98	3.849	2.697	2.965
108	3.083	2.002	3.761	5.451	3.874	3.057	2.837
109	2.917	2.144	4.447	5.627	4.448	2.962	2.135
110	3.038	2.241	4.126	5.763	3.948	2.857	2.474
111	3.096	2.493	3.982	6.285	4.247	2.987	2.451
112	3.224	2.147	4.36	6.09	4.338	2.93	2.359
113	2.973	2.24	4.135	5.483	4.42	3.238	2.509
114	3.288	2.582	4.856	5.877	4.861	2.34	2.15
115	3.113	2.622	4.2	5.358	4.953	3.031	2.29

116	3.588	2.329	4.287	4.139	5.07	2.445	2.56
117	3.43	2.153	5.026	4.427	4.547	2.392	2.231
118	3.537	2.015	4.427	5.29	4.147	2.382	2.076
119	3.246	1.724	4.428	5.223	4.511	2.568	2.748
120	3.437	2.311	4.044	4.664	5.111	2.493	2.192
121	3.31	1.894	4.681	4.606	5.235	2.409	2.084
122	3.33	2.371	4.502	5.056	4.606	2.698	2.398
123	3.318	1.922	4.734	4.383	4.848	2.494	2.74
124	3.059	2.187	4.326	4.835	4.85	2.597	2.474
125	3.1	2.027	4.387	4.855	4.914	2.739	2.076
126	3.309	2.451	4.839	4.287	5.009	2.117	2.761
127	3.023	2.548	4.547	5.11	5.028	2.427	2.132
128	3.221	2.219	4.489	3.492	5.34	2.473	2.781
129	3.141	2.238	4.251	4.524	4.787	2.551	2.335
130	2.972	2.137	4.259	4.606	5.136	2.653	2.374
131	3.151	2.311	3.995	5.575	4.882	2.069	2.157
132	3.05	1.971	4.317	4.742	4.9	2.915	1.906
133	3.018	2.289	3.879	5.345	4.957	2.465	1.974
134	3.566	2.116	4.239	4.054	4.995	2.8	2.256
135	3.28	2.56	4.628	4.79	4.907	2.882	2.236
136	3.061	2.283	4.511	4.7	4.923	2.75	2.785
137	3.088	2.183	4.597	4.924	4.462	2.287	2.299
138	3.172	1.739	4.559	6.115	4.412	2.538	2.278
139	3.234	1.627	4.562	5.09	4.569	2.461	2.251
140	3.406	2.141	4.575	4.539	4.97	2.666	2.158
141	3.299	2.281	4.504	4.928	4.793	2.656	2.292
142	3.052	1.883	4.264	5.197	4.472	2.19	2.367
143	3.352	2.092	4.245	5.048	4.906	2.723	2.441
144	3.535	2.18	4.81	4.618	4.862	2.417	2.461
145	3.248	1.755	4.64	5.224	4.5	2.717	2.178
146	3.359	1.732	5.105	4.227	4.931	2.492	2.524
147	3.138	2.093	4.401	4.145	5.108	2.875	2.415
148	3.574	1.726	4.751	5.458	5.26	2.63	2.181
149	3.155	1.846	4.252	5.057	4.885	2.628	1.969
150	3.317	1.928	4.591	5.446	4.923	3.14	2.026
151	3.123	2.076	4.542	5.886	5.042	2.482	2.257
152	2.893	2.289	4.883	5.581	5.017	2.5	2.102
153	3.072	1.939	4.474	6.04	5.035	3.036	2.346
154	3.131	2.213	4.853	5.836	4.792	2.771	1.894
155	2.887	2.308	4.378	5.238	4.639	3.038	2.225
156	2.86	2.188	4.355	6.175	4.868	1.953	2.185
157	3.302	2.048	4.479	5.557	4.534	2.234	2.484
158	2.745	2.146	4.43	6.616	4.487	2.5	2.268

159	3.243	2.221	4.157	6.341	4.654	2.124	2.255
160	3.268	2.017	4.52	6.214	4.641	2.217	2.101
161	3.327	2.283	4.225	5.157	4.8	1.998	2.153
162	3.153	1.967	5.164	5.453	4.759	2.079	2.122
163	3.201	2.26	4.776	5.467	4.777	2.088	2.209
164	3.647	2.203	5.187	5.827	4.449	2.27	1.974
165	2.807	2.426	4.435	5.631	4.966	2.209	2.077
166	3.144	2.123	4.69	5.91	5.003	2.193	1.99
167	3.107	2.155	4.604	6.4	4.904	2.141	2.512
168	2.729	2.07	4.526	5.995	4.769	2.256	2.157
169	3.027	2.114	4.634	5.032	4.728	2.171	2.204
170	2.794	2.017	4.54	5.304	5.061	2.352	1.894
171	3.39	1.769	5.055	5.383	5.018	2.414	2.34
172	3.549	2.184	4.486	5.37	5.163	2.218	2.412
173	3.12	1.878	4.587	4.828	5.104	2.597	2.137
174	3.186	1.789	5.663	4.91	5.088	2.557	2.235
175	3.427	2.021	4.597	4.213	5.088	2.538	2.517
176	2.826	2.353	4.799	4.632	5.132	2.725	2.645
177	3.089	2.162	4.591	5.124	4.929	2.707	2.821
178	2.679	2.392	4.468	5.2	5.001	2.455	2.677
179	2.792	1.902	4.524	4.717	4.861	2.445	2.525
180	3.023	1.732	5.123	4.82	4.844	2.619	2.967
181	3.013	2.156	4.33	4.891	4.951	2.348	2.774
182	3.391	2.022	4.776	5.964	4.681	3.047	2.586
183	2.957	2.242	4.101	5.973	4.892	3.211	2.155
184	2.917	1.91	4.542	4.643	4.759	2.349	2.176
185	3.153	2.022	4.423	5.108	4.823	2.983	2.601
186	3.643	2.115	4.705	4.645	4.91	2.683	2.468
187	3.22	1.953	5.323	4.589	4.913	2.334	2.642
188	3.378	2.333	5.491	4.629	4.856	2.207	2.553
189	3.018	2.058	4.091	4.348	4.801	2.42	2.421
190	2.961	2.099	4.265	4.821	4.898	2.615	2.369
191	3.02	2.088	4.755	4.921	4.758	2.526	2.604
192	2.991	2.115	4.992	4.324	4.745	2.705	2.403
193	3.099	2.064	5.175	4.739	4.934	2.516	2.563
194	3.014	2.219	4.688	4.419	5.016	2.65	2.598
195	3.069	2.068	4.435	4.698	4.842	2.337	2.38
196	3.065	2.26	4.356	4.115	4.786	2.777	3.05
197	3.002	2.154	4.208	4.34	5.064	2.435	2.564
198	3.185	1.915	5.53	4.373	5.195	2.632	2.673
199	3.586	2.56	4.314	4.298	4.929	2.85	2.508
200	3.007	2.208	4.436	4.24	4.798	2.269	2.544
201	2.934	1.791	4.116	3.471	5.073	2.478	2.306

202	2.954	2.391	4.119	4.416	4.841	2.423	2.71
203	2.859	2.506	3.948	4.127	4.727	2.271	2.076
204	3.121	2.214	3.894	4.258	5.189	2.747	2.197
205	3.338	1.802	3.96	4.007	5.069	2.444	2.441
206	3.458	2.095	4.848	4.008	4.789	2.34	2.536
207	3.09	2.977	3.883	4.017	4.68	2.157	2.628
208	3.261	2.51	4.123	3.739	4.893	2.161	2.293
209	3.039	1.856	4.105	5.012	4.986	1.942	2.392
210	2.866	2.218	3.634	4.274	4.472	1.998	2.495
211	2.822	2.06	4.152	4.537	4.748	2.171	2.783
212	3.175	1.944	4.167	4.497	4.686	2.216	3
213	3	1.976	3.744	5.339	4.793	2.139	2.528
214	2.754	1.806	4.369	4.446	4.75	2.115	2.684
215	3.152	2.209	4.128	3.991	4.917	1.685	2.568
216	3.095	2.573	4.815	4.303	5.016	2.373	2.707
217	2.882	1.749	3.741	4.349	5.205	2.111	2.492
218	3.162	2.55	4.489	4.79	4.984	2.337	2.924
219	3.733	1.899	4.567	4.889	5.218	1.981	2.671
220	2.642	1.813	4.25	4.686	4.905	2.277	2.475
221	2.814	2.203	4.141	4.496	4.669	2.427	2.557
222	2.939	1.636	4.032	4.723	4.783	2.103	2.351
223	3.121	2.088	4.288	4.555	4.893	2.234	2.662
224	3.248	2.093	4.164	4.532	4.849	2.064	2.73
225	3.195	2.125	4.019	4.908	4.259	2.4	2.359
226	3.637	1.897	4.386	4.909	4.402	1.996	2.152
227	3.054	1.827	3.886	4.531	4.079	2.614	2.837
228	3.034	2.407	4.227	4.439	4.182	2.634	2.396
229	3.175	2.161	3.771	4.118	4.174	2.916	2.747
230	3.208	2.576	4.084	3.778	4.587	3.012	2.527
231	3.351	2.549	4.56	4.527	4.844	2.494	2.731
232	3.216	2.789	3.881	4.579	4.886	2.149	2.297
233	3.284	2.323	4.39	4.928	4.492	2.267	2.947
234	3.025	2.848	3.763	4.322	4.203	2.328	2.553
235	2.839	2.624	4.044	4.188	4.272	2.735	2.478
236	2.941	2.486	3.859	4.16	4.649	2.322	2.503
237	2.97	2.713	4.105	4.512	4.238	1.91	2.499
238	3.061	2.327	4.505	3.916	4.163	2.316	2.743
239	3.283	2.314	3.937	4.476	4.356	1.864	2.827
240	3.399	2.15	3.985	4.483	4.76	2.3	2.354
241	3.478	2.498	4.028	4.172	4.551	1.945	2.216
242	3.049	2.552	4.052	4.552	4.515	2.518	2.786
243	3.135	2.113	4.19	5.072	4.677	2.771	2.031
244	3.181	2.675	3.837	4.747	4.747	2.511	2.502

245	3.498	2.075	4.019	6.692	4.76	2.548	2.411
246	3.499	2.584	4.301	6.141	4.4	2.672	2.37
247	3.083	2.277	4.195	5.479	4.781	2.218	2.349
248	3.005	2.42	4.117	4.776	4.43	2.37	2.511
249	3.413	2.303	4.347	4.843	4.427	2.435	2.277
250	3.254	2.051	3.943	5.452	4.662	3.095	2.478
251	3.423	2.244	3.513	4.787	4.557	2.356	2.373
252	3.457	2.413	4.32	4.19	4.628	3.079	2.349
253	3.283	2.209	4.205	5.69	4.493	2.499	2.103
254	3.375	2.573	4.17	5.166	4.688	3.084	2.222
255	3.121	2.182	4.334	5.608	4.822	2.548	2.208
256	3.05	2.463	4.164	5.389	4.778	2.532	2.33
257	3.884	2.471	4.093	5.349	4.445	1.846	2.325
258	3.171	2.353	4.114	4.855	4.678	1.958	2.388
259	3.003	2.486	4.457	5.839	4.727	2.273	2.399
260	3.298	2.101	4.077	5.276	4.426	1.931	2.309
261	3.39	2.013	3.817	5.928	4.576	2.08	2.573
262	3.39	2.445	4.877	4.732	4.609	2.461	2.858
263	3.138	2.029	4.256	5.165	4.457	2.382	2.874
264	3.393	2.101	3.878	5.871	4.551	2.283	2.437
265	3.095	2.063	4.381	5.271	4.417	2.393	1.971
266	3.286	2.37	4.395	5.687	4.587	2.154	2.538
267	3.362	2.32	3.957	5.919	4.428	2.049	2.215
268	3.685	2.405	3.666	5.865	4.567	1.932	2.291
269	3.462	2.402	4.178	5.291	4.265	2.524	2.387
270	3.676	2.228	4.616	4.637	4.378	2.512	2.59
271	3.601	2.06	4.163	4.29	4.184	2.32	2.462
272	3.274	2.56	3.791	4.288	4.846	2.246	2.103
273	3.311	2.348	4.23	4.571	4.734	2.329	2.255
274	3.452	2.291	4.02	4.709	4.438	2.202	2.296
275	3.74	2.551	4.115	3.988	4.924	1.934	2.294
276	3.525	2.218	4.052	3.771	5.487	1.944	2.157
277	3.289	2.336	4.76	4.009	5.318	2.038	2.323
278	3.56	2.125	3.954	4.409	5.856	2.118	2.049
279	3.501	1.918	3.614	4.625	6.037	2.186	2.366
280	3.571	2.163	4.174	4.676	5.42	2.12	2.149
281	3.675	1.821	3.647	4.47	5.646	2.048	2.482
282	3.724	2.089	4.091	5.161	5.672	2.36	2.436
283	3.522	2.327	4.195	4.313	4.948	2.093	2.196
284	3.473	2.154	3.962	4.589	4.885	2.01	2.026
285	3.214	2.547	4.142	4.144	4.735	2.153	2.446
286	3.156	2.233	4.539	4.893	5.226	1.999	2.581
287	3.346	2.251	4.387	4.485	5.096	2.239	2.231

288	3.235	2.302	4.26	4.876	5.097	2.209	2.504
289	3.294	2.679	4.538	3.887	5.161	2.242	2.714
290	3.159	2.767	4.226	4.244	4.816	2.3	2.449
291	3.439	2.294	3.929	4.932	5.016	2.231	2.66
292	3.355	2.313	4.093	5.63	5.062	1.986	2.624
293	3.058	2.895	4.396	4.88	5.199	2.028	2.443
294	3.044	2.505	3.928	4.57	4.586	2.32	2.781
295	2.932	2.666	4.234	4.793	4.902	1.777	2.329
296	3.029	2.127	3.859	4.791	4.865	1.948	2.636
297	3.08	2.597	4.28	4.345	4.844	2.014	2.405
298	3.025	2.452	4.118	3.944	5.091	2.074	2.463
299	2.93	2.44	3.947	4.562	5.252	1.868	2.275
300	2.946	1.902	4.406	4.431	4.794	1.97	2.426
301	2.811	2.433	4.187	5.161	4.803	2.026	2.416
302	3.111	2.823	4.014	5.597	4.697	1.94	2.711
303	3.434	2.491	4.272	4.96	4.974	1.778	2.657
304	3.382	2.197	3.869	4.77	5.24	2.207	2.945
305	3.022	2.577	4.33	5.082	5.145	2.033	2.364
306	3.207	2.414	4.284	4.683	5.022	1.568	2.178
307	2.875	2.235	4.092	4.882	4.89	1.968	2.701
308	3.26	3.016	3.669	4.896	5.151	2.015	2.45
309	2.926	2.662	4.259	5.546	5.009	1.702	2.583
310	3.123	2.387	3.896	5.905	4.731	1.94	2.369
311	3.036	3.174	4.083	4.849	4.941	1.774	2.557
312	3.051	2.724	4.408	4.59	5.174	1.618	2.475
313	2.821	2.446	4.475	5.113	5.413	1.783	2.798
314	3.245	2.456	4.464	4.673	4.858	1.965	2.627
315	2.945	2.449	4.86	4.984	4.841	1.563	2.739
316	3.122	2.553	3.978	4.742	5.01	1.732	2.381
317	3.009	2.532	3.862	4.821	5.18	1.721	2.777
318	3.974	2.25	4.05	4.622	5.084	1.921	2.495
319	3.57	2.371	4.15	5.27	4.97	1.694	2.28
320	3.086	2.423	4.447	5.136	4.932	1.981	2.387
321	3.447	2.367	4.362	5.447	5.193	1.967	2.125
322	3.283	2.157	4.43	5.631	4.828	2.099	2.043
323	3.411	2.346	4.432	5.252	4.755	2.022	2.411
324	3.391	2.109	3.994	4.842	5.06	1.811	2.417
325	3.213	2.491	4.366	5.257	4.945	1.902	2.25
326	3.431	2.451	4.412	4.623	5.113	1.737	2.158
327	3.367	2.359	4.161	5.456	4.902	1.681	2.158
328	3.603	2.429	4.206	4.695	4.924	1.596	2.483
329	2.92	2.769	4.041	4.928	5.182	1.49	2.579
330	3.378	2.284	4.269	4.941	4.944	1.818	2.644

331	3.27	1.627	4.173	5.008	4.947	1.746	2.587
332	3.291	2.382	4.252	5.714	5.061	1.747	2.244
333	3.338	2.029	4.09	5.202	4.795	1.636	2.361
334	3.413	2.094	4.13	4.27	5.181	1.592	2.458
335	3.363	2.164	3.935	4.702	5.074	1.773	2.611
336	3.193	2.373	3.85	4.919	4.858	1.687	2.327
337	3.551	2.078	3.921	4.059	5.249	1.817	2.466
338	3.322	1.495	4.308	4.248	5.186	1.767	2.75
339	3.072	1.816	3.846	4.822	5.224	1.671	2.488
340	3.265	1.762	3.755	5.333	5.437	1.833	2.328
341	3.289	1.762	3.749	4.665	5.213	1.893	2.226
342	3.184	1.945	3.723	4.757	5.223	1.977	2.442
343	3.207	1.782	3.964	4.92	5.178	1.753	2.484
344	3.015	2.498	4.225	4.64	5.353	1.782	2.805
345	3.099	2.19	4.116	5.659	5.131	1.661	2.721
346	3.232	2.548	3.63	4.679	5.291	1.898	2.577
347	3.095	2.395	4.046	6.368	5.25	1.97	2.431
348	3.195	1.661	4.305	5.332	5.018	1.941	2.83
349	3.019	1.692	3.878	4.274	5.041	1.731	2.868
350	3.399	1.937	4.083	5.04	4.88	1.863	2.609
351	3.295	2.387	4.269	5.049	4.815	1.556	2.551
352	3.008	2.402	4.198	4.566	4.933	1.565	2.77
353	3.155	2.237	4.015	5.193	5.015	1.852	2.594
354	3.38	2.052	4.135	4.996	5.039	1.944	2.318
355	3.383	2.03	4.258	4.603	4.938	1.689	2.933
356	3.025	2.082	4.005	4.988	5.209	1.952	2.582
357	3.679	1.824	3.911	5.786	5.093	1.72	2.56
358	3.304	1.814	3.857	6.09	5.101	1.891	2.983
359	3.003	2.127	4.361	5.252	5.278	2.012	2.778
360	3.384	1.619	4.141	5.818	5.305	1.928	2.835
361	2.61	1.774	4.252	5.838	5.349	1.698	2.659
362	2.926	2.141	4.217	5.299	5.298	1.864	2.567
363	3.087	1.717	4.426	5.608	5.393	1.824	2.543
364	3.461	2.042	4.122	5.119	4.888	1.735	2.368
365	3.195	1.749	4.182	5.468	5.038	1.953	2.394
366	3.256	2.051	4.159	5.325	5.177	2.073	2.65
367	3.29	1.594	3.779	5.372	5.287	1.847	2.709
368	3.139	1.871	4.102	5.249	5.195	2.112	2.495
369	3.069	1.748	4.193	5.235	5.024	1.924	2.621
370	3.089	2.11	4.185	5.247	5.252	2.118	2.573
371	2.964	1.744	4.639	5.034	5.291	2.428	2.668
372	3.415	2.284	4.154	5.462	5.231	2.039	3.046
373	3.073	2.281	4.643	5.759	5.068	2.106	2.423

374	3.187	1.867	3.986	5.014	5.321	2.079	2.307
375	3.059	1.85	4.119	5.158	5.322	1.999	2.727
376	3.195	1.819	4.141	5.578	5.151	2.25	2.7
377	2.934	1.715	4.388	5.434	5.118	2.224	2.588
378	3.294	1.875	3.993	5.874	5.174	1.826	2.545
379	3.451	2.14	4.118	5.111	5.35	1.958	2.376
380	3.076	1.748	4.128	6.538	5.147	2.076	2.454
381	3.185	1.883	4.35	5.623	5.613	2.032	2.491
382	3.236	2.302	4.181	5.696	4.908	1.964	2.587
383	3.101	2.198	4.262	5.492	5.369	1.671	2.373
384	3.097	2.069	4.445	5.044	5.34	1.835	2.599
385	3.022	2.088	4.111	5.326	5.517	2	2.358
386	3.253	1.829	4.254	5.702	5.4	2.009	2.435
387	3.255	2.122	4.195	5.313	5.247	2.047	2.283
388	3.414	1.778	4	5.615	5.331	2.203	2.655
389	3.183	1.852	4.186	5.529	5.505	2.333	2.677
390	2.886	2.309	4.134	6.379	5.527	2.075	2.416
391	3.037	2.224	4.056	6.227	5.312	2.074	2.777
392	3.382	2.398	4.259	5.723	5.275	1.945	2.889
393	3.464	2.066	3.819	4.986	5.153	2.126	2.579
394	3.628	2.592	4.072	5.416	5.209	1.883	2.635
395	3.405	2.739	4.391	5.343	5.167	1.797	2.469
396	3.194	2.719	4.532	5.534	5.245	1.77	2.156
397	3.179	2.566	3.996	5.487	5.253	1.712	2.655
398	3.106	2.148	4.43	4.566	5.274	1.7	2.497
399	3.08	2.28	4.107	4.971	5.176	1.878	2.394
400	2.92	2.396	4.226	4.714	4.782	1.797	2.48
401	2.669	2.421	4.66	5.649	4.9	2.043	2.328
402	2.734	2.269	4.589	4.955	5.355	1.726	2.336
403	3.103	2.459	4.275	5.399	5.416	1.735	2.162
404	3.036	2.509	4.405	4.823	5.278	1.737	2.178
405	2.828	2.132	4.256	4.074	5.262	1.669	2.325
406	2.767	2.349	4.043	4.741	5.479	1.584	2.507
407	2.616	2.13	3.97	5.279	5.407	1.67	2.46
408	2.883	2.241	4.329	4.605	5.427	1.797	2.662
409	2.729	2.391	3.858	5.273	5.576	1.735	2.514
410	2.929	2.462	4.284	5.783	5.4	1.688	2.482
411	2.819	2.258	4.044	5.019	5.581	1.867	2.675
412	2.716	2.726	4.441	5.423	5.228	1.823	2.501
413	2.851	2.082	4.363	5.598	5.715	1.522	2.232
414	2.286	2.307	4.542	5.727	5.396	1.912	2.114
415	2.844	2.386	4.009	4.831	5.265	1.817	2.315
416	2.774	2.574	3.863	4.078	5.495	1.909	2.491

417	2.77	2.313	3.919	4.158	5.259	1.766	2.584
418	2.739	2.058	3.841	4.114	5.267	1.791	2.163
419	2.646	2.224	3.765	4.98	5.167	1.696	2.095
420	2.775	2.471	4.191	4.793	5.212	1.481	2.161
421	2.906	2.334	4.131	4.256	5.485	1.573	2.301
422	2.796	2.696	4.071	3.942	5.282	1.963	2.718
423	2.778	2.106	4.271	4.478	5.483	1.803	2.486
424	2.939	2.721	3.729	4.287	5.008	1.796	2.617
425	2.874	2.282	3.597	4.689	5.597	1.655	2.183
426	3.101	2.38	4.145	3.989	4.923	1.908	2.53
427	2.997	2.097	3.736	4.399	5.012	1.652	2.384
428	3.118	2.212	3.86	4.08	5.513	2.14	2.421
429	2.778	2.217	3.801	3.585	5.13	1.697	2.714
430	3.161	2.285	3.685	3.983	5.556	1.52	2.522
431	2.899	2.395	3.728	4.444	5.413	1.538	2.407
432	2.851	2.655	4.028	3.641	5.282	1.848	2.71
433	2.788	2.405	3.926	3.469	5.083	1.804	2.295
434	3.062	2.415	4.21	2.82	5.178	1.572	2.317
435	2.809	2.242	4.245	3.153	5.531	1.699	2.241
436	2.976	2.111	3.763	3.23	5.121	1.78	2.337
437	2.719	2.157	3.929	3.551	5.104	1.896	2.553
438	2.905	2.254	3.93	3.493	5.102	1.851	2.484
439	3.236	2.737	4.349	3.459	5.034	1.557	2.549
440	2.847	2.388	3.99	2.864	5.166	1.712	2.929
441	2.536	2.523	3.944	3.295	5.342	1.905	2.663
442	2.907	2.614	3.643	4.147	5.345	1.743	2.112
443	2.856	2.481	3.844	3.8	5.226	1.85	2.201
444	3.12	2.168	3.695	3.739	5.327	1.551	2.485
445	2.768	2.75	4.048	3.855	5.272	2.046	2.458
446	2.851	2.562	3.718	4.051	5.029	1.674	2.666
447	3.255	2.757	3.623	4.722	5.146	1.877	2.185
448	2.697	2.212	3.996	3.779	5.233	2.142	2.275
449	2.986	2.581	3.774	4.414	5.027	1.769	2.719
450	2.956	2.341	3.718	4.856	5.354	1.755	2.257
451	3.101	2.575	4.054	4.551	5.494	1.634	2.272
452	3.388	1.845	4.079	4.257	5.408	1.754	2.349
453	2.888	1.987	3.655	3.72	5.117	1.887	2.304
454	3.16	2.132	4.061	4.185	5.188	1.583	2.473
455	3.346	2.113	3.805	4.009	5.515	1.692	2.286
456	2.839	2.421	3.781	4.004	5.177	1.778	2.387
457	2.753	2.256	3.904	3.86	4.951	1.611	2.294
458	3.245	2.04	4.063	4.304	5.095	1.948	2.412
459	3.009	2.262	3.904	4.065	5.23	1.867	2.334

460	2.742	2.306	4.262	3.697	5.352	1.932	2.662
461	3.498	2.527	4.201	3.793	5.007	1.648	2.588
462	3.345	2.385	4.091	3.652	5.14	1.666	2.438
463	2.937	2.095	3.957	3.155	5.049	1.387	2.219
464	3.19	2.113	4.291	4.231	5.202	1.6	2.123
465	3.176	2.474	4.061	4.839	5.309	2.354	2.462
466	2.878	2.408	4.619	5.449	5.127	1.856	2.304
467	3.239	2.404	4.455	4.355	5.283	1.796	2.194
468	2.644	1.621	3.898	4.647	5.654	1.875	2.245
469	2.686	1.725	3.896	5.194	5.162	1.751	2.698
470	2.475	1.849	3.923	5.045	5.499	2.042	2.665
471	2.588	1.916	4.294	4.943	5.385	2.155	2.575
472	2.59	1.972	4.51	4.178	5.331	2.083	2.397
473	3.097	1.827	4.169	3.986	5.267	2.255	2.233
474	2.593	1.821	4.099	4.149	5.125	2.058	2.682
475	2.747	2.026	4.129	4.503	5.395	2.177	2.568
476	2.823	2.504	4.158	4.069	5.264	1.98	2.577
477	2.801	1.934	4.135	4.326	5.116	1.748	2.355
478	2.375	1.881	3.922	4.594	4.981	1.796	2.287
479	2.659	1.713	3.894	4.107	5.346	1.835	2.649
480	3.148	1.955	4.107	4.539	5.328	1.534	2.524
481	3.105	1.976	4.062	3.961	5.461	1.912	2.791
482	3.108	2.268	4.043	4.009	5.259	1.605	2.607
483	2.518	1.844	4.467	4.76	5.152	1.748	2.087
484	2.594	1.515	4.052	3.911	5.028	1.893	2.359
485	2.496	1.89	3.957	4.297	5.241	1.755	2.075
486	2.683	1.779	3.658	4.247	5.163	2.332	2.295
487	2.326	1.933	3.74	4.974	5.507	1.815	1.823
488	3.209	1.941	3.881	4.892	5.067	1.735	2.12
489	2.649	1.908	3.842	5.005	5.172	1.832	1.985
490	3.045	1.802	3.997	5.134	5.304	1.919	2.082
491	3.105	1.901	3.919	4.165	5.105	1.911	2.321
492	2.878	1.926	4	4.435	5.126	2.072	2.231
493	3.157	1.961	3.971	4.365	5.225	2.325	2.89
494	2.799	1.74	4.963	4.831	5.156	2.087	2.322
495	2.61	1.739	4.395	5.396	5.179	2.061	2.424
496	3.067	1.673	4.326	4.642	5.273	1.783	1.8
497	2.513	1.715	5.334	4.285	5.081	1.901	1.925
498	2.832	2.009	4.085	4.633	5.048	1.585	2.562
499	2.752	2.05	4.781	5.828	5.076	1.755	2.124
500	2.877	1.787	4.15	5.26	5.111	1.841	2.29
501	2.924	1.731	4.269	5.459	5.055	1.821	2.294

Table S8. Protein RMSF of the Tested Compounds

Protein RMSF

Residue#	BNS1	BNS2	BNS3	BNS4	BNS11	BNS16	JBJ-125
0	7.261	5.182	4.87	4.989	6.868	5.278	4.206
1	5.253	3.68	3.221	3.668	4.949	4.058	2.526
2	3.832	2.371	2.443	2.524	3.509	2.655	2.041
3	3.405	2.237	2.198	2.349	3.274	2.335	1.825
4	2.114	1.889	1.762	1.881	2.938	2.331	1.448
5	1.634	1.442	1.467	1.409	2.266	1.856	1.243
6	1.204	1.137	1.05	1.106	1.564	1.34	0.89
7	0.946	0.884	0.794	0.953	1.023	1.371	0.684
8	0.939	0.962	0.786	1.044	1.071	1.114	0.74
9	0.904	0.888	0.704	1.034	1.001	0.913	0.672
10	1.02	1.029	0.799	1.256	1.35	0.969	0.849
11	1.176	1.131	0.939	1.497	1.721	1.097	1.083
12	1.165	1.249	0.959	1.626	1.899	1.115	1.073
13	0.993	1.181	0.812	1.379	1.731	0.988	0.976
14	0.961	1.069	0.787	1.413	1.752	0.937	1.326
15	1.029	1.105	0.836	1.518	1.954	0.965	1.078
16	1.207	1.254	0.983	1.728	2.299	1.041	1.094
17	1.328	1.28	1.13	1.785	2.823	1.251	1.27
18	1.324	1.125	1.097	1.708	2.88	1.157	1.17
19	1.484	1.134	1.181	1.814	3.118	1.256	1.195
20	1.557	1.163	1.273	1.822	3.032	1.445	1.154
21	1.554	1.102	1.297	1.872	2.945	1.756	1.221
22	1.582	1.338	1.649	2.201	3.211	2.092	1.695
23	1.481	1.518	1.848	2.327	3.211	2.565	1.912
24	1.523	2.052	2.017	2.437	3.434	2.782	1.885
25	1.375	1.872	1.684	2.16	2.671	1.911	1.64
26	1.345	1.015	1.246	1.843	2.613	1.428	1.461
27	1.233	0.842	0.96	1.616	2.439	1.087	0.981
28	1.166	0.805	0.932	1.536	2.369	0.97	0.885
29	0.998	0.818	0.828	1.375	2.139	0.854	0.82
30	1.049	0.901	0.85	1.388	2.286	0.886	0.947
31	0.975	0.902	0.787	1.283	2.117	0.871	0.946
32	0.954	1.026	0.77	1.288	2.03	0.897	1.138
33	0.936	0.994	0.768	1.262	1.988	0.903	1.118
34	1.114	1.26	1.069	1.573	2.172	1.19	1.574
35	1.317	1.474	1.404	1.815	2.445	1.512	1.935
36	1.752	1.877	2.083	2.227	3.17	1.961	2.496
37	2.188	2.185	3.177	2.785	4.268	2.362	3.193
38	1.866	2.013	2.312	2.513	3.669	1.93	2.434

39	1.863	2.222	2.236	2.657	3.704	2.074	2.385
40	1.574	1.756	1.716	2.155	3.12	1.665	1.937
41	1.34	1.516	1.309	1.855	2.758	1.342	1.625
42	1.02	1.052	0.864	1.344	2.245	0.993	1.163
43	0.951	0.952	0.821	1.242	2.125	0.922	1.005
44	0.804	0.754	0.692	1.081	1.852	0.725	0.713
45	0.748	0.681	0.621	1.028	1.512	0.641	0.619
46	0.786	0.677	0.65	1.196	1.623	0.759	0.667
47	0.845	0.667	0.653	1.231	1.595	0.771	0.576
48	1.085	0.853	0.796	1.501	2.111	0.92	0.715
49	1.296	1.004	1.058	1.59	2.402	1.095	0.79
50	1.588	1.406	1.544	1.849	2.916	1.379	0.955
51	1.644	1.273	1.389	1.752	2.988	1.301	0.908
52	1.677	1.266	1.546	1.865	3.138	1.313	1.067
53	1.698	1.091	1.327	1.726	2.915	1.255	1.113
54	1.829	1.174	1.415	1.861	3.104	1.356	1.312
55	1.935	1.331	1.45	1.772	2.994	1.376	1.356
56	2.049	1.507	1.456	1.939	3.217	1.414	1.485
57	1.783	1.261	1.196	1.689	2.905	1.24	1.168
58	1.557	1.108	1.022	1.256	2.33	1.048	0.872
59	1.639	1.274	1.09	1.355	2.496	1.111	0.952
60	1.555	1.32	1.145	1.542	2.635	1.173	0.997
61	1.27	1.041	0.872	1.226	2.096	0.969	0.786
62	1.196	1.021	0.791	1.134	1.926	0.883	0.733
63	1.314	1.194	0.958	1.474	2.411	1.083	0.948
64	1.214	1.144	0.988	1.271	2.15	1.128	0.915
65	0.97	0.98	0.879	0.929	1.595	0.996	0.665
66	1.039	1.036	0.869	1.008	1.931	0.894	0.676
67	1.236	1.054	0.961	0.989	2.083	0.965	0.684
68	1.145	0.803	0.882	0.798	1.529	0.805	0.575
69	1.105	0.887	0.855	0.829	1.706	0.83	0.649
70	1.249	0.952	0.908	0.92	2.19	0.867	0.641
71	0.928	0.868	0.791	0.892	1.934	0.87	0.676
72	1.066	1.075	0.926	0.716	1.878	1.099	0.921
73	0.614	0.726	0.597	0.632	1.014	0.721	0.621
74	0.586	0.683	0.534	0.617	0.851	0.635	0.554
75	0.513	0.578	0.489	0.569	0.6	0.57	0.5
76	0.54	0.632	0.523	0.612	0.686	0.584	0.511
77	0.555	0.636	0.573	0.673	0.854	0.541	0.519
78	0.584	0.579	0.603	0.621	0.988	0.533	0.53
79	0.574	0.572	0.571	0.626	1.041	0.654	0.555
80	0.609	0.606	0.619	0.681	1.052	0.608	0.532
81	0.625	0.579	0.514	0.703	0.63	0.617	0.483

82	0.716	0.614	0.508	0.807	0.657	0.706	0.5
83	0.827	0.695	0.605	1.009	1.004	0.809	0.546
84	1.055	0.94	0.995	1.124	1.363	1.006	0.685
85	1.076	1.12	1.423	1.39	1.758	1.034	0.708
86	1.335	1.256	1.661	1.57	2.505	1.276	0.84
87	1.032	1.022	1.033	1.666	2.186	1.019	0.719
88	0.986	0.855	0.756	1.31	1.759	0.889	0.599
89	0.801	0.634	0.605	1.035	1.185	0.749	0.507
90	0.689	0.549	0.533	0.969	0.988	0.679	0.481
91	0.561	0.516	0.47	0.783	0.694	0.6	0.485
92	0.603	0.576	0.521	0.761	1.022	0.562	0.547
93	0.658	0.618	0.605	0.806	1.103	0.602	0.608
94	0.795	0.69	0.69	0.826	1.314	0.68	0.707
95	0.814	0.857	0.669	0.875	0.957	0.707	0.659
96	0.98	0.94	0.807	0.996	1.158	0.921	0.77
97	0.76	0.872	0.748	1.005	1.036	0.767	0.72
98	0.655	0.708	0.673	0.77	0.915	0.653	0.636
99	0.616	0.643	0.618	0.673	0.872	0.593	0.627
100	0.657	0.592	0.567	0.59	0.823	0.543	0.666
101	0.847	0.692	0.686	0.644	1.003	0.651	0.771
102	0.735	0.744	0.714	0.713	1.163	0.685	0.839
103	0.672	0.689	0.712	0.669	1.092	0.634	0.865
104	0.735	0.692	0.871	0.661	1.07	0.658	0.941
105	0.788	0.841	1.16	0.781	1.37	0.788	1.043
106	0.959	0.973	1.323	0.881	1.54	0.852	1.196
107	1.015	0.866	1.414	0.88	1.415	0.761	1.326
108	1.126	0.82	2.768	0.889	1.499	0.777	1.234
109	1.257	0.907	2.268	0.959	1.487	0.851	1.473
110	1.254	1.004	1.234	1.08	1.51	0.885	1.734
111	1.119	0.986	0.896	0.778	1.324	0.682	1.078
112	1.042	1.122	0.755	0.676	1.198	0.639	0.821
113	1.029	0.93	0.597	0.583	0.965	0.56	0.689
114	0.787	0.753	0.549	0.576	0.913	0.545	0.611
115	0.565	0.676	0.589	0.565	0.877	0.513	0.609
116	0.569	0.623	0.547	0.566	0.739	0.5	0.56
117	0.542	0.505	0.511	0.536	0.644	0.483	0.491
118	0.503	0.489	0.534	0.56	0.688	0.491	0.488
119	0.487	0.48	0.517	0.551	0.659	0.477	0.466
120	0.485	0.47	0.499	0.521	0.589	0.469	0.459
121	0.469	0.476	0.466	0.509	0.56	0.472	0.464
122	0.487	0.494	0.479	0.525	0.606	0.509	0.459
123	0.491	0.506	0.485	0.529	0.592	0.518	0.458
124	0.497	0.525	0.476	0.535	0.585	0.53	0.472

125	0.546	0.594	0.504	0.562	0.675	0.568	0.505
126	0.569	0.62	0.512	0.571	0.679	0.587	0.52
127	0.555	0.662	0.533	0.585	0.659	0.6	0.578
128	0.608	0.703	0.576	0.643	0.791	0.646	0.639
129	0.648	0.741	0.597	0.65	0.863	0.726	0.618
130	0.708	0.868	0.637	0.691	0.801	0.849	0.696
131	0.862	0.974	0.777	0.791	0.884	1.101	0.888
132	0.978	1.033	0.905	0.901	1.089	1.227	0.907
133	0.919	1.138	0.856	0.903	1.068	1.163	0.883
134	1.032	1.311	0.918	0.943	1.058	1.231	0.971
135	1.128	1.393	0.823	0.884	1.077	1.121	0.825
136	1.109	1.459	0.75	0.817	0.981	0.888	0.671
137	0.713	1.079	0.791	0.709	0.808	0.73	0.612
138	0.641	1.196	0.95	0.813	0.978	0.902	0.887
139	0.576	0.846	0.746	0.72	0.911	0.828	0.97
140	0.579	0.737	0.661	0.699	0.939	0.686	0.734
141	0.569	0.629	0.658	0.642	0.912	0.65	0.56
142	0.63	0.598	0.609	0.6	0.919	0.676	0.544
143	0.654	0.649	0.606	0.661	0.965	0.647	0.644
144	0.526	0.567	0.558	0.618	0.797	0.562	0.609
145	0.55	0.557	0.526	0.593	0.745	0.506	0.515
146	0.537	0.559	0.533	0.603	0.752	0.52	0.511
147	0.523	0.589	0.529	0.621	0.816	0.534	0.522
148	0.602	0.68	0.579	0.706	0.931	0.593	0.576
149	0.62	0.604	0.606	0.639	0.885	0.577	0.6
150	0.671	0.642	0.674	0.652	1.102	0.652	0.687
151	0.697	0.647	0.705	0.654	1.075	0.641	0.631
152	0.564	0.56	0.521	0.624	0.816	0.548	0.527
153	0.515	0.536	0.506	0.587	0.641	0.499	0.487
154	0.494	0.555	0.507	0.584	0.635	0.528	0.482
155	0.476	0.561	0.508	0.589	0.644	0.532	0.46
156	0.505	0.618	0.527	0.64	0.699	0.572	0.506
157	0.661	0.901	0.577	0.709	0.772	0.642	0.613
158	0.798	1.091	0.631	1.013	0.814	0.878	0.679
159	1.225	1.373	0.834	1.265	0.877	1.332	0.749
160	1.301	1.376	1.049	3.233	1.097	2.074	0.971
161	1.831	1.754	1.138	2.82	1.086	2.205	0.863
162	2.023	2.07	1.387	2.696	1.339	3.497	1.168
163	2.167	2.089	2.023	2.321	1.439	3.679	1.351
164	2.401	2.281	3.299	3.936	1.41	4.326	1.37
165	3.117	2.77	3.485	4.388	2.365	5.292	1.383
166	3.991	3.358	2.738	6.119	2.924	6.632	1.469
167	3.875	3.435	2.444	6.408	2.634	6.026	1.389

168	3.102	2.788	2.099	4.929	1.866	4.38	1.503
169	3.011	2.862	2.712	4.81	2.029	4.224	1.762
170	3.341	3.375	3.314	6.457	2.836	4.813	1.871
171	3.351	3.566	3.543	6.523	2.967	4.344	1.867
172	3.477	3.876	3.399	5.515	2.758	3.685	1.914
173	4.589	4.06	3.626	5.046	3.086	4.464	2.175
174	4.133	3.193	3.385	4.701	2.595	4.463	2.139
175	3.134	3.118	3.012	3.536	2.427	3.364	1.969
176	2.168	3.583	3.05	3.101	2.44	2.607	1.742
177	2.139	3.092	3.948	2.751	2.676	2.626	1.505
178	1.658	2.568	2.955	2.248	2.285	2.332	1.257
179	1.275	1.884	1.27	1.593	2.551	1.433	0.979
180	0.997	1.435	0.977	1.365	2.097	1.295	0.798
181	0.686	0.979	0.774	1.065	1.953	0.883	0.713
182	0.607	1.023	0.691	0.841	2.383	0.678	0.669
183	0.727	1.386	0.784	0.763	2.18	0.745	0.617
184	0.708	1.464	0.825	0.749	2.188	0.778	0.638
185	0.795	1.309	0.951	0.964	2.095	0.813	0.763
186	0.75	1.076	0.844	0.95	2.056	0.782	0.768
187	0.829	1.366	0.902	0.939	2.024	0.862	0.751
188	1.002	1.565	1.093	1.068	1.721	1	0.849
189	1.089	1.51	1.201	1.189	1.867	1.106	0.988
190	1.07	1.36	1.101	1.091	1.77	1.122	1.013
191	1.017	1.45	1.04	1.068	1.992	1.075	0.997
192	0.834	1.589	0.879	1.425	1.709	1	0.878
193	0.833	1.325	0.905	0.817	1.76	1.031	0.861
194	0.692	0.963	0.777	0.835	1.812	1.216	0.83
195	0.696	0.903	0.753	0.716	1.737	0.843	0.668
196	0.659	0.861	0.694	0.666	1.4	0.806	0.617
197	0.552	0.783	0.656	0.628	1.277	0.707	0.597
198	0.468	0.706	0.56	0.552	0.975	0.496	0.518
199	0.442	0.599	0.52	0.524	0.845	0.462	0.49
200	0.458	0.612	0.537	0.542	0.885	0.466	0.505
201	0.458	0.574	0.553	0.528	0.853	0.441	0.476
202	0.449	0.527	0.528	0.494	0.684	0.433	0.445
203	0.444	0.551	0.489	0.515	0.716	0.476	0.488
204	0.496	0.569	0.591	0.546	0.826	0.522	0.481
205	0.523	0.553	0.668	0.541	0.777	0.504	0.498
206	0.491	0.533	0.538	0.515	0.668	0.469	0.516
207	0.546	0.554	0.55	0.56	0.718	0.494	0.524
208	0.594	0.599	0.647	0.598	0.818	0.527	0.59
209	0.582	0.596	0.621	0.546	0.756	0.528	0.625
210	0.631	0.617	0.625	0.589	0.745	0.541	0.659

211	0.637	0.64	0.693	0.678	0.832	0.594	0.73
212	0.676	0.687	0.776	0.7	0.914	0.621	0.801
213	0.754	0.847	0.911	0.792	1.026	0.732	0.92
214	0.723	0.817	0.894	0.81	1.034	0.737	0.832
215	0.807	0.883	0.93	0.898	1.206	0.806	0.854
216	0.789	0.897	0.906	0.918	1.268	0.838	0.812
217	0.994	1.18	1.109	1.176	1.714	1.084	0.955
218	1.386	1.398	1.443	1.595	2.187	1.414	1.6
219	1.863	1.813	1.82	2.239	2.618	1.818	1.598
220	1.45	1.479	1.507	1.714	2.309	1.557	1.339
221	1.444	1.533	1.569	1.85	2.421	1.741	1.466
222	1.517	1.494	1.699	2.014	2.461	1.939	1.452
223	1.69	1.676	1.895	2.374	2.844	2.367	1.503
224	1.504	1.553	1.668	2.063	2.703	2.092	1.357
225	1.189	1.341	1.385	1.622	2.275	1.519	1.062
226	1.255	1.532	1.497	1.772	2.503	1.537	1.091
227	1.29	1.589	1.376	1.794	2.293	1.525	1.152
228	1.048	1.324	1.058	1.469	1.712	1.249	1.003
229	0.969	1.382	1.137	1.46	1.758	1.08	0.938
230	1.176	1.633	1.33	1.696	1.988	1.229	1.11
231	1.183	1.52	1.22	1.651	1.654	1.269	1.147
232	1.097	1.33	1.106	1.441	1.523	1.207	1.053
233	0.877	1.157	0.889	1.202	1.326	1.001	0.854
234	0.794	0.847	0.758	0.88	0.977	0.813	0.736
235	0.722	0.728	0.691	0.762	0.902	0.684	0.669
236	0.751	0.756	0.727	0.765	0.908	0.685	0.7
237	0.751	0.775	0.702	0.717	0.857	0.657	0.701
238	0.793	0.859	0.735	0.735	0.887	0.682	0.749
239	0.979	1.018	0.888	0.88	1.006	0.785	0.954
240	0.967	0.885	0.753	0.771	0.934	0.671	0.879
241	0.889	0.842	0.705	0.754	0.876	0.665	0.766
242	0.761	0.795	0.664	0.763	0.815	0.647	0.748
243	0.706	0.784	0.644	0.799	0.825	0.67	0.779
244	0.694	0.79	0.674	0.801	0.844	0.682	0.756
245	0.632	0.68	0.593	0.663	0.759	0.609	0.652
246	0.646	0.692	0.596	0.664	0.77	0.629	0.664
247	0.614	0.705	0.625	0.713	0.836	0.653	0.681
248	0.552	0.685	0.593	0.641	0.785	0.607	0.615
249	0.551	0.661	0.551	0.605	0.774	0.591	0.578
250	0.615	0.738	0.58	0.688	0.916	0.657	0.622
251	0.591	0.724	0.591	0.668	0.969	0.643	0.608
252	0.567	0.691	0.58	0.633	0.942	0.611	0.567
253	0.653	0.788	0.644	0.722	1.084	0.698	0.641

254	0.689	0.805	0.709	0.764	1.478	0.755	0.67
255	0.78	0.955	0.869	0.923	1.798	0.863	0.786
256	0.807	0.95	0.912	0.929	1.71	0.943	0.842
257	0.881	1.088	1.013	0.968	1.911	1.06	0.831
258	1.03	1.183	1.155	1.103	2.021	1.281	1.008
259	0.948	1.003	0.989	0.993	1.87	1.127	0.906
260	0.732	0.742	0.724	0.776	1.451	0.811	0.694
261	0.681	0.75	0.708	0.808	1.094	0.735	0.702
262	0.634	0.718	0.637	0.71	0.894	0.659	0.613
263	0.611	0.682	0.599	0.654	0.751	0.61	0.568
264	0.692	0.75	0.668	0.771	0.849	0.725	0.683
265	0.657	0.686	0.626	0.774	0.837	0.661	0.687
266	0.606	0.59	0.573	0.711	0.77	0.604	0.623
267	0.6	0.558	0.585	0.763	0.805	0.614	0.625
268	0.657	0.625	0.65	0.895	0.918	0.669	0.708
269	0.691	0.648	0.666	0.826	0.88	0.661	0.732
270	0.667	0.541	0.615	0.846	0.838	0.563	0.601
271	0.721	0.62	0.707	1.079	1.124	0.642	0.699
272	0.83	0.725	0.8	1.029	1.218	0.745	0.822
273	0.863	0.652	0.689	1.02	0.971	0.652	0.69
274	0.884	0.7	0.705	1.356	1.179	0.72	0.694
275	0.975	0.84	0.816	1.375	1.334	0.825	0.769
276	0.987	0.792	0.761	1.251	1.044	0.678	0.664
277	0.986	0.827	0.677	1.097	0.926	0.621	0.715
278	1.31	1.094	1.011	1.286	1.076	0.718	0.863
279	1.254	1.054	1.012	1.21	1.097	0.761	0.852
280	0.909	0.847	0.716	0.841	0.899	0.627	0.737
281	1.113	0.935	0.824	0.877	0.931	0.704	0.959
282	1.218	1.127	1.047	1.081	1.104	0.714	1.156
283	1.875	1.218	1.301	1.202	1.207	0.767	1.388
284	2.551	1.735	2.158	1.684	1.596	0.924	2.215
285	3.922	1.536	2.391	2.232	1.588	0.994	2.552
286	4.401	1.584	2.557	2.06	1.811	0.89	2.66
287	3.677	1.447	2.308	2.184	2.241	1.164	3.051
288	3.452	1.567	2.33	2.012	2.373	1.313	2.594
289	2.546	1.792	2.205	1.798	2.363	1.335	2.26
290	2.034	1.892	1.716	1.405	2.923	1.189	2.47
291	1.85	1.91	1.381	1.18	3.214	1.218	2.981
292	2.09	1.394	1.224	1.118	3.222	1.253	2.772
293	2.398	1.618	1.358	1.119	3.969	1.92	3.055
294	2.35	1.682	1.377	1.219	4.477	2.646	3.058
295	1.729	1.795	1.556	1.442	4.619	2.455	2.982
296	1.836	1.68	1.393	1.237	3.44	2.011	2.424

297	2.112	1.736	1.073	0.994	2.598	2.298	1.785
298	1.804	1.715	1.268	1.105	3.249	2.081	1.787
299	1.456	1.438	1.422	1.264	3.312	1.749	1.796
300	1.765	1.309	1.326	1.209	2.469	1.077	1.435
301	2.064	1.471	0.949	1.114	2.205	1.42	1.723
302	1.934	1.991	1.077	1.409	2.507	1.966	1.819
303	1.689	1.873	1.227	1.638	2.794	1.958	1.938
304	1.569	2.008	1.114	1.361	2.429	1.425	1.546
305	1.123	1.397	0.931	1.044	1.661	0.961	1.159
306	1.09	1.527	1.089	1.165	1.346	0.966	1.178
307	0.975	1.689	1.046	0.932	1.192	0.949	1.025
308	1.172	1.377	1.131	0.987	1.386	1.034	1.14
309	0.998	0.974	1.479	0.854	1.404	0.988	0.956
310	1.048	0.958	1.597	0.861	1.802	0.97	1.013
311	1.199	0.987	1.508	0.892	1.332	0.861	1.217
312	1.25	0.961	1.156	0.807	1.461	0.774	1.189
313	1.616	1.037	0.999	0.909	1.363	0.844	1.107
314	1.843	2.031	0.957	1.271	2.243	1.557	1.278
315	1.771	1.867	1.179	1.662	3.548	1.328	1.395
316	3.002	3.159	1.808	2.715	4.658	2.491	2.093

Table S9. rGyr value of tested Copmounds

rGyr

frame#	BNS1	BNS2	BNS3	BNS4	BNS11	BNS16	JBJ-125
0	5.106	4.487	3.889	4.67	5.571	5.079	6.069
1	5.036	4.841	4.059	4.758	5.518	4.855	5.976
2	5.203	4.773	4.025	5.106	5.447	4.979	5.88
3	5.217	4.821	4.057	5.011	5.478	4.835	5.996
4	5.218	4.73	4.403	5.179	5.376	4.946	6.059
5	5.22	4.708	4.241	4.879	5.435	4.923	6.112
6	5.336	4.666	4.268	4.974	5.477	4.941	6.034
7	5.224	4.721	4.321	4.916	5.428	4.833	5.989
8	5.009	4.692	4.407	4.751	5.6	4.938	6.037
9	5.127	4.7	4.515	5.111	5.434	4.908	6.056
10	5.153	4.721	4.494	5.109	5.425	4.999	6.105
11	5.215	4.737	4.471	5.031	5.458	5.032	6.01
12	5.296	4.605	4.575	5.156	5.504	4.879	6.009
13	5.155	4.753	4.59	4.972	5.616	5.038	6.023
14	5.076	4.675	4.501	4.775	5.71	5.084	6.042
15	5.324	4.654	4.231	4.977	5.635	4.962	6.036
16	5.276	4.772	4.308	5.082	5.743	5.064	6.031
17	5.169	4.658	4.359	5.213	5.725	5.029	6.118
18	5.214	4.67	4.428	5.197	5.759	5.152	6.104
19	5.339	4.765	4.306	5.192	5.569	5.054	6.09
20	5.315	4.681	4.429	5.085	5.715	5.095	6.1
21	5.253	4.764	4.524	5.162	5.883	5.064	6.08
22	5.289	4.617	4.544	5.082	5.662	5.005	5.999
23	5.433	4.683	4.519	4.8	5.807	5.031	6.084
24	5.268	4.626	4.452	5.073	5.656	5.052	6.122
25	5.274	4.557	4.54	5.182	5.616	4.945	6.103
26	5.153	4.731	4.592	4.959	5.548	4.94	6.059
27	5.178	4.74	4.678	4.856	5.425	4.96	6.068
28	5.021	4.654	4.579	4.764	5.527	4.99	6.034
29	5.255	4.589	4.656	4.829	5.551	4.965	6.053
30	5.092	4.643	4.555	5.072	5.377	4.97	6.091
31	5.108	4.62	4.359	4.955	5.469	5.037	5.917
32	5.293	4.738	4.55	4.977	5.275	5.206	6.07
33	5.273	4.824	4.544	5.149	5.065	5.057	6.08
34	5.144	4.704	4.478	5.155	5.155	5.169	6.064
35	5.129	4.651	4.493	5.102	5.143	5.085	6.122
36	5.201	4.61	4.596	5.058	5.138	5.142	6.035
37	5.026	4.692	4.515	5.059	5.359	5.159	6.031
38	5.204	4.631	4.243	4.928	4.707	5.114	6.066

39	5.179	4.797	4.293	4.962	4.913	5.153	6.114
40	5.24	4.73	4.349	4.933	5.345	5.137	6.042
41	5.338	4.765	4.538	5.012	5.192	5.116	6.038
42	5.211	4.637	4.259	4.832	5.174	5.054	6.009
43	5.283	4.606	4.54	4.973	5.127	5.115	5.964
44	5.32	4.597	4.401	4.864	4.991	5.023	6.043
45	5.201	4.809	4.477	4.945	5.145	5.167	6.035
46	5.23	4.754	4.514	5.052	4.718	5.157	6.032
47	5.162	4.705	4.392	4.886	5.065	5.124	6.016
48	5.374	4.7	4.503	4.971	4.973	5.105	6.021
49	5.232	4.627	4.396	4.717	4.833	5.119	6.087
50	5.224	4.659	4.626	4.774	5.133	5.121	6.059
51	5.264	4.619	4.632	4.815	5.067	5.166	5.935
52	5.237	4.746	4.681	4.752	4.941	5.11	6.037
53	5.245	4.715	4.6	5.065	5.097	5.119	5.996
54	5.325	4.83	4.669	5.116	5.139	5.147	5.972
55	5.381	4.764	4.661	5.166	5.177	5.119	5.936
56	5.285	4.592	4.651	5.137	4.972	5.163	5.993
57	5.284	4.641	4.522	5.125	4.791	5.151	5.99
58	5.132	4.653	4.537	5.151	4.713	5.163	6.01
59	5.287	4.648	4.687	5.008	4.861	5.077	6.083
60	5.251	4.693	4.604	4.968	4.751	5.143	5.948
61	5.216	4.657	4.564	4.995	5.031	5.126	5.993
62	5.291	4.725	4.686	4.895	4.816	5.103	6.004
63	5.265	4.648	4.785	4.823	4.892	5.212	6.087
64	5.266	4.698	4.624	4.847	4.954	5.021	5.994
65	5.322	4.733	4.759	4.745	4.885	5.052	6.016
66	5.282	4.675	4.66	4.919	4.951	5.109	6.146
67	5.348	4.674	4.519	4.838	4.799	5.06	6.164
68	5.216	4.646	4.377	4.78	4.804	5.123	6.081
69	5.101	4.779	4.552	4.844	4.976	4.954	6.046
70	5.246	4.703	4.539	4.786	4.805	5.041	6.171
71	5.313	4.671	4.528	4.964	4.969	5.117	6.114
72	5.441	4.701	4.557	5.139	5.167	5.119	6.152
73	5.148	4.638	4.685	5.161	5.152	5.163	6.197
74	5.34	4.752	4.595	5.081	4.844	5.135	6.205
75	5.224	4.607	4.603	5.079	4.793	5.152	6.174
76	5.261	4.679	4.701	4.997	4.804	5.054	6.157
77	5.266	4.686	4.705	5.03	4.965	5.096	6.08
78	5.215	4.797	4.674	5.25	4.717	5.066	6.158
79	5.294	4.711	4.67	5.139	5.059	5.1	6.192
80	5.33	4.767	4.573	5.068	5.029	5.145	6.081
81	5.083	4.763	4.607	5.126	5.077	5.033	6.018

82	5.2	4.749	4.673	4.87	5.058	5.09	6.052
83	5.254	4.68	4.59	5.028	5.306	5.112	6.121
84	5.267	4.776	4.7	5.223	5.114	5.077	6.02
85	5.176	4.753	4.674	5.031	5.063	5.152	5.997
86	5.253	4.71	4.538	5.01	4.879	5.011	6.148
87	5.214	4.632	4.552	5.043	5.092	4.982	6.067
88	5.227	4.655	4.652	4.701	5.036	5.013	6.067
89	5.231	4.794	4.622	4.842	5.163	5.084	6.181
90	5.249	4.821	4.657	4.963	5.1	5.139	6.078
91	5.273	4.771	4.623	4.902	4.852	5.154	6.146
92	5.412	4.757	4.595	5.009	5.032	5.14	6.189
93	5.28	4.808	4.708	4.927	4.899	5.134	6.094
94	5.25	4.631	4.589	5.169	5.096	5.186	6.074
95	5.231	4.728	4.73	4.925	4.985	5.065	6.056
96	5.388	4.707	4.578	5.044	4.988	5.069	6.034
97	5.344	4.775	4.648	5.144	5.005	5.09	6.066
98	5.222	4.68	4.593	5.256	4.773	5.116	6.143
99	5.394	4.598	4.45	4.877	5.036	5.09	6.138
100	5.245	4.761	4.574	5.103	5.087	5.147	6.037
101	5.316	4.642	4.555	4.959	5.082	5.172	6.084
102	5.173	4.626	4.548	5.054	5.099	5.091	6.137
103	5.33	4.667	4.505	5.2	4.71	5.108	6.129
104	5.268	4.692	4.661	4.975	4.897	5.146	6.038
105	5.498	4.798	4.798	4.934	4.917	5.107	6.067
106	5.16	4.709	4.731	5.036	4.898	5.216	6.024
107	5.338	4.674	4.585	4.86	5.176	5.149	6.074
108	5.208	4.671	4.584	4.968	4.928	5.109	6.005
109	5.355	4.778	4.748	5.031	4.846	5.08	6.031
110	5.374	4.635	4.589	4.859	4.738	5.157	6.041
111	5.318	4.731	4.538	5.03	4.995	5.077	5.956
112	5.314	4.746	4.619	5.085	4.852	5.074	6.122
113	5.319	4.741	4.604	5.045	4.848	5.128	6.056
114	5.362	4.589	4.606	4.948	4.984	5.24	6.064
115	5.246	4.665	4.548	5.031	5.087	5.141	6.109
116	5.313	4.582	4.596	4.991	5.018	5.166	6.09
117	5.33	4.736	4.66	4.926	4.967	5.222	6.011
118	5.24	4.63	4.623	5.177	4.775	5.171	6.086
119	5.307	4.702	4.594	5.26	4.95	5.194	5.964
120	5.265	4.675	4.627	4.85	4.903	5.021	6.06
121	5.273	4.676	4.574	4.932	4.791	5.167	6.079
122	5.337	4.507	4.534	5.103	4.89	5.111	6.126
123	5.382	4.837	4.72	5.074	5.109	5.138	6.062
124	5.315	4.656	4.572	5.147	5.087	5.175	5.975

125	5.265	4.724	4.554	5.048	4.899	5.063	6.052
126	5.369	4.791	4.606	5.141	4.932	5.218	6.045
127	5.28	4.755	4.609	5.08	5.118	5.096	5.942
128	5.1	4.8	4.602	5.187	5.149	5.162	6.107
129	5.399	4.786	4.603	5.203	4.789	5.037	6.05
130	5.231	4.764	4.66	5.093	4.893	5.16	5.898
131	5.306	4.776	4.564	5.092	4.5	5.12	6.021
132	5.226	4.737	4.596	5.21	4.894	5.05	5.883
133	5.281	4.767	4.584	5.268	4.755	5.13	5.958
134	5.411	4.72	4.616	5.127	4.681	5.086	5.988
135	5.302	4.648	4.559	5.242	4.664	5.143	6.013
136	5.259	4.545	4.631	5.007	4.872	5.187	6.01
137	5.362	4.715	4.672	5.084	4.996	5.147	6.078
138	5.33	4.773	4.666	5.239	5.105	5.188	6.087
139	5.361	4.722	4.594	5.092	4.933	5.257	6.053
140	5.303	4.572	4.522	4.927	4.972	5.069	5.947
141	5.33	4.67	4.548	5.041	5.043	5.185	5.932
142	5.26	4.752	4.593	5.022	5.229	5.167	5.924
143	5.551	4.642	4.591	5.22	4.765	5.125	6.092
144	5.404	4.74	4.651	5.035	4.857	5.196	6.005
145	5.295	4.73	4.753	5.04	5.22	5.074	6.003
146	5.327	4.788	4.518	4.937	5.011	5.139	5.996
147	5.337	4.82	4.607	5.023	4.818	5.125	6.009
148	5.178	4.694	4.554	5.215	4.77	5.125	5.985
149	5.366	4.728	4.628	5.058	4.947	5.087	5.954
150	5.314	4.703	4.633	5.262	5.029	4.994	6.021
151	5.153	4.817	4.537	5.064	4.809	5.096	5.922
152	5.25	4.711	4.595	5.184	4.853	5.134	5.96
153	5.36	4.81	4.685	5.189	4.799	5.176	5.968
154	5.156	4.869	4.666	5.017	4.817	5.249	5.984
155	5.397	4.782	4.558	4.942	4.865	5.139	6.011
156	5.233	4.712	4.733	5.208	4.818	4.947	5.849
157	5.178	4.677	4.601	4.983	4.937	4.996	5.928
158	5.344	4.722	4.701	4.962	4.872	5.078	5.947
159	5.13	4.701	4.66	4.937	4.661	5.207	5.927
160	5.337	4.777	4.516	5.088	5.019	5.203	5.892
161	5.204	4.763	4.557	5.25	5.248	5.128	5.921
162	5.294	4.715	4.644	4.999	5.043	5.107	6.015
163	5.388	4.667	4.544	5.091	5.019	5.215	5.91
164	5.251	4.684	4.562	5.271	4.903	5.165	5.976
165	5.249	4.577	4.525	5.14	4.957	5.129	5.971
166	5.294	4.767	4.634	5.236	4.999	5.148	5.971
167	5.278	4.787	4.523	5.19	5.056	5.156	6.014

168	5.313	4.706	4.545	5.183	5.023	5.07	6.017
169	5.314	4.752	4.621	5.038	4.944	5.1	5.947
170	5.311	4.731	4.577	5.309	5.024	5.083	5.917
171	5.29	4.785	4.629	5.118	4.914	5.122	6.038
172	5.37	4.727	4.558	5.076	4.903	5.116	6.08
173	5.26	4.844	4.582	5.058	5.053	5.161	5.941
174	5.197	4.738	4.68	4.973	4.987	5.108	5.885
175	5.168	4.785	4.721	5.132	5.003	5.112	5.972
176	5.2	4.717	4.583	5.107	4.721	5.061	6.119
177	5.249	4.762	4.534	5.116	4.685	5.143	6.047
178	5.228	4.818	4.601	5.076	4.896	5.142	6.015
179	5.222	4.679	4.555	5.113	4.888	5.205	6.017
180	5.162	4.761	4.552	5.184	4.712	5.102	5.993
181	5.217	4.712	4.655	5.22	4.869	5.068	6.015
182	5.226	4.72	4.542	5.205	5.097	5.17	6.008
183	5.28	4.804	4.654	5.243	4.881	5.156	5.963
184	5.302	4.732	4.473	5.1	4.83	5.157	6.023
185	5.31	4.719	4.605	5.203	4.879	5.163	5.971
186	5.424	4.729	4.635	5.094	4.796	5.12	6.028
187	5.306	4.785	4.518	5.052	5.097	5.126	5.945
188	5.286	4.774	4.539	5.021	4.949	5.148	5.938
189	5.293	4.611	4.561	5.168	5.008	5.109	6.062
190	5.311	4.705	4.709	5.098	5.005	5.087	5.967
191	5.219	4.738	4.566	5.186	4.889	5.13	6.023
192	5.22	4.704	4.515	5.112	4.863	5.125	6.038
193	5.228	4.754	4.688	5.265	4.978	5.125	6.034
194	5.396	4.775	4.605	4.841	4.942	5.13	6.087
195	5.259	4.681	4.468	4.904	4.729	5.067	6.047
196	5.307	4.69	4.598	4.966	5.259	5.027	6.077
197	5.238	4.667	4.609	5.03	4.661	5.05	5.942
198	5.333	4.718	4.617	5.078	4.698	4.98	5.967
199	5.294	4.694	4.533	5.021	4.922	5.142	6.084
200	5.179	4.752	4.556	5.08	4.889	5.095	6.022
201	5.281	4.669	4.572	4.809	4.879	5.08	6.029
202	5.102	4.741	4.678	5.171	5.149	5.12	6.078
203	5.247	4.698	4.596	5.109	4.757	5.166	5.964
204	5.285	4.747	4.594	5.084	4.83	5.105	5.906
205	5.22	4.702	4.635	5.017	5.166	5.071	5.972
206	5.367	4.774	4.685	5.11	4.925	5.101	5.9
207	5.318	4.898	4.594	5.076	4.766	5.028	6.027
208	5.252	4.822	4.645	4.95	4.871	5.088	5.915
209	5.321	4.56	4.553	5.202	4.8	5.1	5.981
210	5.184	4.822	4.616	5.173	4.932	5.149	6.067

211	5.23	4.733	4.688	5.143	4.875	5.051	5.999
212	5.235	4.616	4.635	5.054	4.735	4.988	5.981
213	5.236	4.673	4.584	5.163	5.01	5.116	6.113
214	5.223	4.577	4.544	5.117	4.912	5.053	5.958
215	5.285	4.673	4.623	5.075	4.66	5.111	6.052
216	5.273	4.754	4.604	5.091	4.769	5.115	6.035
217	5.271	4.723	4.493	5.119	5.193	5.07	6.099
218	5.281	4.969	4.562	5.11	5.131	5.096	6.006
219	5.328	4.761	4.49	5.22	5.05	4.994	6.036
220	5.203	4.688	4.653	5.153	4.866	5.228	6.055
221	5.352	4.749	4.603	5.12	5.026	5	6.033
222	5.368	4.69	4.617	5.106	4.92	5.078	6.043
223	5.302	4.567	4.576	5.12	5.102	5.102	6.075
224	5.259	4.636	4.703	5.259	4.98	5.07	6.045
225	5.171	4.61	4.558	5.194	5.112	5.048	6.149
226	5.196	4.619	4.781	5.2	4.827	5.006	6.064
227	5.261	4.628	4.554	5.107	5.052	4.974	6.061
228	5.356	4.705	4.59	5.133	5.087	5.021	6.174
229	5.207	4.654	4.426	5.147	4.945	5.063	6.063
230	5.221	4.793	4.589	5.238	4.985	5.041	6.083
231	5.372	4.786	4.645	5.126	4.958	5.059	6.069
232	5.232	4.773	4.497	5.045	5.236	4.981	5.997
233	5.273	4.804	4.691	5.168	4.761	5.068	6.075
234	5.261	4.86	4.604	5.323	4.706	5.017	6.059
235	5.272	4.761	4.6	5.156	4.615	5.076	6.022
236	5.267	4.781	4.606	5.018	4.923	5.089	6.132
237	5.321	4.785	4.506	5.157	4.881	5.104	6.09
238	5.343	4.647	4.679	5.086	4.888	4.962	5.949
239	5.176	4.751	4.547	5.05	4.702	5.066	6.089
240	5.273	4.738	4.663	5.136	4.896	4.969	5.961
241	5.148	4.777	4.633	5.108	4.857	5.04	6.093
242	5.289	4.7	4.636	5.189	5.312	4.86	6.019
243	5.246	4.849	4.665	5.211	5.006	5.062	6.048
244	5.219	4.821	4.5	5.168	5.025	4.911	6.068
245	5.049	4.729	4.698	5.332	4.773	4.934	5.988
246	5.359	4.756	4.501	5.213	5.028	4.93	6.089
247	5.147	4.78	4.69	5.074	5.057	5.147	6.066
248	5.212	4.83	4.595	4.932	5.172	4.975	6.137
249	5.287	4.855	4.614	5.201	5.111	5.018	6.037
250	5.267	4.701	4.704	5.235	5.057	4.992	6.071
251	5.315	4.826	4.633	5.029	5.261	4.986	6.138
252	5.207	4.741	4.624	5.133	5.187	5.142	6.058
253	5.293	4.896	4.661	5.138	5.006	5.012	6.08

254	5.316	4.728	4.585	5.138	5.005	5.042	6.113
255	5.252	4.744	4.609	5.066	5.149	5.162	6.056
256	5.26	4.707	4.659	5.101	5.014	4.983	6.126
257	5.397	4.928	4.583	5.048	5.095	5.105	6.038
258	5.29	4.778	4.521	5.197	5.071	5.083	6.067
259	5.234	4.814	4.619	5.234	4.962	5.08	6.069
260	5.363	4.75	4.726	5.194	5.075	5.174	6.083
261	5.31	4.728	4.644	5.226	5.163	5.207	6.084
262	5.248	4.797	4.567	5.142	4.897	5.188	6.121
263	5.308	4.746	4.703	5.268	5.113	5.108	6.082
264	5.329	4.87	4.518	5.215	5.341	5.123	5.962
265	5.24	4.702	4.608	5.199	5.151	5.182	6.077
266	5.349	4.73	4.627	5.128	5.069	5.151	6.113
267	5.352	4.804	4.743	5.242	5.243	5.119	6.143
268	5.259	4.773	4.592	5.079	4.953	5.162	6.08
269	5.387	4.847	4.646	5.276	5.055	5.062	6.043
270	5.369	4.775	4.775	5.056	5.14	5.172	6.101
271	5.393	4.776	4.572	5.077	4.958	5.157	6.089
272	5.297	4.813	4.595	5.092	5.503	5.168	6.042
273	5.357	4.748	4.631	5.138	5.259	5.113	6.033
274	5.299	4.741	4.605	5.027	5.12	5.181	6.112
275	5.309	4.747	4.608	4.993	4.946	5.236	6.109
276	5.368	4.846	4.717	5.067	5.129	5.225	6.033
277	5.183	4.798	4.651	4.763	4.928	5.232	6.12
278	5.392	4.863	4.711	5.134	4.98	5.172	6.045
279	5.261	4.849	4.601	5.11	5.004	5.1	5.979
280	5.269	4.841	4.6	5.149	4.987	5.229	6.166
281	5.313	4.847	4.647	5.025	5.074	5.177	6.025
282	5.251	4.859	4.686	5.078	5.036	5.173	6.194
283	5.291	4.867	4.679	5.094	5.074	5.023	6.105
284	5.223	4.798	4.535	5.168	5.19	5.129	6.057
285	5.403	4.684	4.728	5.115	5.075	5.121	6.031
286	5.231	4.868	4.756	5.208	5.102	5.155	6.105
287	5.316	4.722	4.55	5.064	4.189	5.142	6.084
288	5.252	4.749	4.67	5.188	4.253	5.135	6.062
289	5.272	4.864	4.756	5.146	4.21	5.112	6.076
290	5.395	4.706	4.648	5.03	4.342	5.169	6.141
291	5.28	4.784	4.567	5.309	4.468	5.262	6.148
292	5.256	4.745	4.573	5.158	4.339	5.053	6.035
293	5.311	4.797	4.681	5.313	4.223	5.185	6.067
294	5.394	4.785	4.584	5.255	4.341	5.159	6.031
295	5.297	4.858	4.635	5.123	4.315	5.129	6.044
296	5.269	4.612	4.601	5.161	4.34	5.136	6.176

297	5.286	4.595	4.711	5.129	4.213	5.181	6.05
298	5.382	4.708	4.704	5.177	4.243	5.146	6.181
299	5.294	4.815	4.601	5.203	4.266	5.149	6.119
300	5.375	4.779	4.577	5.161	4.254	5.174	6.161
301	5.21	4.886	4.577	5.163	4.304	5.107	6.09
302	5.286	4.86	4.587	5.192	4.252	5.177	6.108
303	5.427	4.81	4.635	5.152	4.131	5.113	6.146
304	5.26	4.768	4.585	5.07	4.312	5.125	6.075
305	5.314	4.583	4.667	5.247	4.261	5.115	6.075
306	5.308	4.638	4.647	5.063	4.18	5.164	6.112
307	5.346	4.597	4.545	5.002	4.159	5.029	6.113
308	5.362	4.617	4.566	5.132	4.442	5.158	6.048
309	5.269	4.701	4.609	5.179	4.235	5.134	6.148
310	5.255	4.722	4.663	5.255	4.283	5.093	6.053
311	5.316	4.614	4.717	5.164	4.254	5.134	6.111
312	5.293	4.711	4.636	5.143	4.312	5.087	6.096
313	5.336	4.693	4.752	5.089	4.291	5.147	6.116
314	5.34	4.664	4.636	5.211	4.196	5.122	6.118
315	5.269	4.567	4.508	5.109	4.227	5.139	6.139
316	5.405	4.641	4.627	5.071	4.291	5.127	6.159
317	5.27	4.63	4.652	5.155	4.398	5.17	6.146
318	5.426	4.591	4.625	5.15	4.208	5.15	6.107
319	5.265	4.699	4.593	5.096	4.299	5.148	6.104
320	5.261	4.54	4.786	5.147	4.415	5.013	6.089
321	5.328	4.604	4.66	5.249	4.288	5.038	6.144
322	5.226	4.532	4.757	4.992	4.354	5.079	6.102
323	5.383	4.664	4.641	5.208	4.201	4.935	6.161
324	5.194	4.554	4.609	5.085	4.305	5.23	6.105
325	5.356	4.649	4.775	5.021	4.227	4.982	6.124
326	5.261	4.686	4.618	4.891	4.307	5.101	6.069
327	5.272	4.504	4.711	5.198	4.289	5.183	6.072
328	5.383	4.694	4.522	5.228	4.477	5.153	6.139
329	5.315	4.682	4.618	4.987	4.346	5.071	6.151
330	5.343	4.748	4.537	5.112	4.398	5.131	6.139
331	5.288	4.48	4.678	5.078	4.361	5.243	6.046
332	5.238	4.61	4.638	5.289	4.188	5.131	6.13
333	5.26	4.678	4.593	5.141	4.317	5.071	6.094
334	5.244	4.725	4.596	5.14	4.23	5.149	6.063
335	5.206	4.793	4.625	5.103	4.284	5.183	6.222
336	5.337	4.644	4.678	5.191	4.147	5.1	6.142
337	5.223	4.636	4.608	5.071	4.361	5.103	6.097
338	5.373	4.724	4.675	5.188	4.3	5.198	6.128
339	5.378	4.713	4.559	5.094	4.342	5.063	6.112

340	5.363	4.743	4.602	5.148	4.381	4.969	6.066
341	5.412	4.692	4.634	5.06	4.373	5.179	6.084
342	5.311	4.617	4.58	5.249	4.328	5.086	6.134
343	5.311	4.713	4.725	5.173	4.232	4.99	6.019
344	5.271	4.704	4.641	5.067	4.354	5.161	6.13
345	5.344	4.708	4.736	5.126	4.32	5.022	6.057
346	5.309	4.825	4.658	5.152	4.315	5.135	6.007
347	5.206	4.794	4.677	5.273	4.312	5.074	6.179
348	5.343	4.819	4.666	5.197	4.383	5.055	6.04
349	5.23	4.66	4.692	4.97	4.211	5.113	6.116
350	5.346	4.717	4.709	4.881	4.399	5.117	6.074
351	5.277	4.686	4.773	5.001	4.184	5.132	6.088
352	5.221	4.794	4.881	4.802	4.166	5.066	6.108
353	5.317	4.751	4.666	4.989	4.137	5.124	6.097
354	5.235	4.736	4.672	5.049	4.291	5.206	6.067
355	5.365	4.569	4.7	5.014	4.277	5.113	6.01
356	5.395	4.572	4.582	5.12	4.357	5.081	6.099
357	5.264	4.517	4.658	5.174	4.216	5.105	6.056
358	5.222	4.551	4.538	5.289	4.354	5.137	6.113
359	5.324	4.64	4.606	5.168	4.354	5.123	6.225
360	5.348	4.636	4.529	5.21	4.329	5.155	6.093
361	5.328	4.657	4.666	5.121	4.314	5.076	5.987
362	5.295	4.491	4.683	5.212	4.328	5.037	6.037
363	5.333	4.6	4.568	5.155	4.228	5.116	6.169
364	5.235	4.74	4.614	4.995	4.273	5.097	6.136
365	5.275	4.685	4.656	5.165	4.361	5.122	6.118
366	5.213	4.657	4.733	5.11	4.245	5.123	6.112
367	5.285	4.438	4.649	5.12	4.213	5.121	6.118
368	5.301	4.46	4.59	5.228	4.324	5.184	6.173
369	5.22	4.559	4.623	5.16	4.258	5.134	6.23
370	5.193	4.459	4.717	5.177	4.309	5.073	6.066
371	5.276	4.621	4.54	4.969	4.267	5.126	6.158
372	5.194	4.778	4.789	5.088	4.264	5.106	6.068
373	5.251	4.618	4.711	5.14	4.29	5.116	6.129
374	5.303	4.613	4.582	5.132	4.21	5.223	6.176
375	5.294	4.629	4.684	5.012	4.318	5.178	6.121
376	5.186	4.545	4.617	5.124	4.355	5.15	6.164
377	5.363	4.613	4.65	5.21	4.25	5.087	6.118
378	5.31	4.454	4.517	5.094	4.244	4.981	6.079
379	5.201	4.643	4.503	4.967	4.334	5.09	6.151
380	5.428	4.629	4.65	5.103	4.334	5.178	6.174
381	5.188	4.669	4.692	5.159	4.291	5.057	6.073
382	5.268	4.782	4.645	4.948	4.34	5.071	6.002

383	5.347	4.71	4.651	5.133	4.237	5.177	6.024
384	5.365	4.585	4.637	5.041	4.378	5.055	6.069
385	5.299	4.673	4.63	5.118	4.303	5.059	6.081
386	5.249	4.689	4.712	5.156	4.267	5.048	6.1
387	5.171	4.745	4.638	5.172	4.271	5.188	6.014
388	5.11	4.565	4.569	5.181	4.299	5.031	6.193
389	5.37	4.68	4.776	5.202	4.294	5.107	6.146
390	5.303	4.808	4.721	5.028	4.228	4.997	6.037
391	5.322	4.564	4.521	5.202	4.337	5.084	6.131
392	5.285	4.693	4.538	5.211	4.275	5.085	6.133
393	5.291	4.551	4.603	5.067	4.202	5.142	6.192
394	5.34	4.481	4.659	5.093	4.252	4.995	6.116
395	5.381	4.664	4.642	5.105	4.248	5.113	6.079
396	5.323	4.631	4.556	5.035	4.345	5.134	6.098
397	5.38	4.738	4.627	4.999	4.201	5.105	6.052
398	5.414	4.671	4.735	4.927	4.298	5.199	6.11
399	5.272	4.548	4.664	5.017	4.219	5.076	6.105
400	5.344	4.593	4.646	4.963	4.285	5.116	6.177
401	5.365	4.621	4.554	5.218	4.282	5.009	6.063
402	5.314	4.674	4.692	5.007	4.344	5.071	6.087
403	5.254	4.537	4.646	5.184	4.293	5.1	6.101
404	5.341	4.719	4.596	5.106	4.363	5.087	5.964
405	5.25	4.647	4.647	5.034	4.334	5.122	6.102
406	5.271	4.663	4.58	4.967	4.332	5.119	5.999
407	5.307	4.58	4.64	5.087	4.277	5.117	6.007
408	5.203	4.592	4.672	5.18	4.302	5.108	6.005
409	5.327	4.718	4.669	5.064	4.203	5.127	6.103
410	5.313	4.446	4.632	5.235	4.33	5.107	6.007
411	5.283	4.568	4.599	5.049	4.271	5.136	6.012
412	5.344	4.531	4.657	5.064	4.226	5.036	6.103
413	5.302	4.663	4.668	5.058	4.278	5.103	6.028
414	5.267	4.633	4.713	5.001	4.326	5.08	6.049
415	5.311	4.594	4.633	5.184	4.265	5.122	6.129
416	5.357	4.705	4.557	5.061	4.301	5.04	6.075
417	5.262	4.585	4.577	4.93	4.345	5.07	6.105
418	5.226	4.639	4.648	5.024	4.333	5.124	6.073
419	5.248	4.604	4.598	5.238	4.39	5.075	6.113
420	5.309	4.646	4.624	5.237	4.359	5.03	6.074
421	5.266	4.732	4.567	4.944	4.329	5.03	6.092
422	5.213	4.789	4.642	5.143	4.222	5.006	6.099
423	5.341	4.644	4.574	4.965	4.276	5.015	6.098
424	5.312	4.577	4.608	5.059	4.305	5.04	6.185
425	5.355	4.5	4.604	5.161	4.31	4.916	6.072

426	5.258	4.561	4.654	5.102	4.278	4.805	6.045
427	5.162	4.674	4.733	5.007	4.32	4.992	6.094
428	5.293	4.669	4.676	5.048	4.192	5.026	6.111
429	5.356	4.705	4.616	5.081	4.293	4.849	5.962
430	5.3	4.521	4.638	5.151	4.241	5.118	5.99
431	5.275	4.63	4.612	4.955	4.301	5.145	6.001
432	5.289	4.645	4.732	4.854	4.383	5.066	6.056
433	5.348	4.687	4.589	5.021	4.254	5.1	6.066
434	5.265	4.664	4.642	4.982	4.317	5.077	6.14
435	5.361	4.45	4.611	5.064	4.15	5.023	6.06
436	5.166	4.653	4.553	5.106	4.295	4.967	6.004
437	5.255	4.605	4.661	5.075	4.285	4.91	6
438	5.226	4.656	4.793	5.042	4.303	4.996	6.11
439	5.311	4.729	4.679	5.103	4.207	5.158	6.168
440	5.243	4.549	4.679	5.062	4.198	5.136	6.154
441	5.251	4.54	4.767	5.167	4.204	4.978	6.062
442	5.297	4.614	4.638	5.104	4.238	5.032	6.049
443	5.319	4.681	4.594	5.139	4.171	4.972	6.117
444	5.387	4.645	4.623	4.959	4.238	5.165	6.045
445	5.333	4.622	4.516	5.035	4.278	4.965	6.015
446	5.342	4.569	4.579	5.072	4.267	4.953	6.1
447	5.189	4.623	4.713	5.125	4.245	4.985	6.149
448	5.379	4.473	4.608	5.016	4.308	4.965	6.08
449	5.307	4.32	4.713	4.977	4.288	5.039	6.106
450	5.282	4.51	4.59	4.999	4.341	5.062	6.11
451	5.174	4.693	4.629	5.209	4.214	5.065	6.078
452	5.381	4.59	4.671	5.25	4.287	5.069	6.054
453	5.263	4.662	4.693	5.114	4.247	5.019	6.146
454	5.311	4.569	4.612	5.13	4.146	5.202	5.992
455	5.322	4.648	4.759	5.22	4.256	5.002	6.123
456	5.225	4.584	4.621	5.08	4.26	4.971	6.111
457	5.204	4.676	4.652	5.09	4.301	5.081	6.183
458	5.271	4.411	4.565	5.086	4.319	5.188	5.866
459	5.324	4.676	4.675	5.123	4.305	5.115	6.059
460	5.238	4.543	4.659	5.072	4.222	5.015	5.952
461	5.256	4.697	4.675	5.113	4.2	4.905	6.101
462	5.277	4.703	4.753	5.042	4.23	4.88	6.145
463	5.199	4.58	4.692	5.084	4.216	4.975	6.202
464	5.189	4.615	4.729	5.218	4.19	4.982	6.103
465	5.252	4.691	4.635	5.205	4.293	4.805	6.152
466	5.126	4.594	4.652	5.185	4.227	4.894	6.149
467	5.274	4.569	4.645	5.213	4.246	5.012	6.062
468	5.267	4.626	4.601	5.106	4.243	5.006	6.175

469	5.174	4.631	4.729	4.939	4.255	4.926	6.124
470	5.31	4.518	4.591	5.04	4.299	4.959	6.118
471	5.244	4.544	4.689	5.072	4.266	4.863	6.068
472	5.348	4.67	4.754	4.924	4.195	4.93	6.045
473	5.266	4.513	4.658	5.127	4.246	4.922	6.173
474	5.255	4.421	4.582	5.043	4.279	4.959	6.157
475	5.223	4.551	4.522	5.066	4.169	5.043	6.116
476	5.336	4.643	4.701	5.016	4.384	4.98	6.163
477	5.232	4.614	4.65	5.033	4.336	4.939	6.097
478	5.258	4.687	4.592	5.166	4.247	5.05	6.049
479	5.289	4.588	4.715	5.211	4.221	4.905	6.034
480	5.139	4.7	4.643	5.231	4.143	4.983	6.084
481	5.326	4.567	4.643	5.246	4.277	5.101	6.106
482	5.269	4.671	4.596	5.047	4.274	5.04	6.081
483	5.361	4.637	4.617	5.178	4.359	5.019	6.058
484	5.31	4.675	4.722	5.203	4.387	5.07	6.034
485	5.389	4.677	4.616	5.185	4.293	5.125	6.035
486	5.383	4.655	4.658	5.073	4.278	5.064	6.063
487	5.27	4.625	4.632	5.047	4.279	4.989	6.146
488	5.349	4.667	4.587	5.113	4.257	5.075	6.099
489	5.3	4.621	4.717	5.229	4.356	5.028	6.037
490	5.474	4.732	4.616	5.251	4.292	4.971	6.104
491	5.338	4.669	4.564	5.075	4.238	4.954	6.072
492	5.339	4.527	4.622	4.959	4.252	5.028	6.046
493	5.26	4.497	4.799	5.146	4.152	4.99	6.069
494	5.349	4.579	4.56	5.174	4.249	4.989	6.034
495	5.348	4.708	4.648	5.194	4.312	5.048	5.98
496	5.163	4.624	4.623	5.071	4.362	4.972	6.024
497	5.458	4.554	4.665	4.947	4.319	5.038	6.102
498	5.35	4.689	4.712	5.155	4.26	5.01	6.083
499	5.284	4.656	4.636	5.108	4.21	5.056	5.959
500	5.321	4.557	4.602	5.214	4.391	5.079	6.199
501	5.22	4.625	4.671	5.163	4.256	4.964	6.072

Table S10. MOLSA value of the tested Compounds

MolSA

frame#	BNS1	BNS2	BNS3	BNS4	BNS11	BNS16	JBJ-125
0	462.733	425.903	362.324	420.86	410.961	402.511	475.66
1	461.834	422.328	376.984	422.092	413.577	408.001	477.889
2	466.636	431.444	371.167	417.459	413.661	410.434	473.377
3	462.92	427.251	379.53	418.781	416.81	406.546	476.929
4	464.072	425.799	392.593	416.596	414.049	407.383	477.839
5	466.79	423.902	388.5	420.458	412.818	407.697	480.135
6	468.341	425.5	385.891	416.954	415.51	413.123	481.202
7	466.803	429.686	388.918	417.898	413.839	407.31	480.971
8	462.583	428.432	389.707	416.285	416.983	405.069	480.967
9	463.754	428.576	389.118	418.591	411.498	406.7	481.222
10	466.745	424.536	394.202	417.013	413.422	405.825	480.343
11	467.361	422.583	394.586	421.82	414.874	407.609	479.938
12	467.341	419.352	391.177	422.167	414.456	404.246	482.187
13	466.199	425.702	395.554	419.274	414.306	407.119	480.489
14	461.011	426.428	393.709	420.458	416.28	408.779	483.875
15	467.781	422.978	388.53	420.008	416.444	404.64	480.854
16	466.983	426.642	392.093	421.343	418.672	406.017	482.38
17	466.11	421.274	392.363	420.276	417.463	406.601	486.616
18	463.122	426.543	393.125	423.252	415.791	408.474	483.741
19	463.226	428.195	391.173	420.33	419.076	404.634	480.765
20	468.311	429.341	393.374	418.361	416.946	406.719	478.306
21	468.048	427.724	395.478	418.52	416.612	406.913	481.469
22	467.356	425.725	396.325	422.111	418.401	407.259	480.029
23	467.813	428.72	394.742	414.776	416.728	407.228	479.254
24	469.306	419.382	392.018	422.286	416.193	408.184	484.022
25	467.202	422.604	393.46	425.886	415.403	405.656	477.481
26	466.333	423.755	393.465	420.257	417.627	407.098	479.692
27	463.901	432.319	396.593	420.7	413.866	407.144	479.905
28	462.234	422.22	393.318	418.677	416.376	407.76	480.71
29	464.776	421.448	393.53	422.19	410.36	406.742	481.618
30	463.366	428.919	392.538	418.694	413.395	405.815	483.8
31	464.966	424.882	395.056	424.978	410.276	406.283	475.79
32	462.888	429.979	393.457	426.032	410.105	409.544	481.82
33	465.202	428.562	395.367	419.419	401.44	408.521	479.23
34	460.342	427.504	390.424	421.176	408.6	410.09	480.971
35	462.8	424.438	394.439	420.592	404.215	407.089	480.671
36	467.688	429.468	393.503	424.371	407.11	406.093	480.279
37	463.903	420.993	395.569	422.087	409.633	410.496	481.531
38	462.602	423.388	390.68	420.562	396.999	409.182	483.743

39	463.61	426.193	394.024	418.154	402.603	406.229	478.391
40	468.726	429.943	391.398	420.544	409.206	403.427	480.838
41	469.204	427.828	391.112	421.898	409.316	407.088	477.669
42	471.355	428.9	385.46	415.169	405.76	404.887	479.132
43	469.148	422.9	393.197	422.079	407.357	406.116	473.611
44	466.966	418.658	390.12	421.05	407.403	405.002	478.501
45	466.542	424.489	392.506	417.85	410.947	408.721	477.402
46	468.86	426.053	394.846	422.335	398.933	407.617	479.335
47	469.588	428	388.957	421.465	401.003	406.949	479.359
48	468.468	428.264	390.127	421.867	402.561	406.21	477.648
49	468.984	429.604	391.663	421.574	406.368	403.657	478.88
50	467.248	424.167	397.654	418.291	404.408	406.92	479.413
51	468.748	423.32	392.605	419.016	403.792	409.397	473.873
52	465.279	429.175	398.465	418.191	402.013	403.535	480.821
53	469.359	429.01	390.963	420.383	407.371	408.161	480.595
54	469.282	428.581	390.095	425.841	409.202	405.058	479.023
55	468.861	426.373	387.844	421.986	405.707	404.811	477.251
56	466.17	421.102	385.52	424.909	400.999	406.673	477.074
57	466.09	427.385	388.782	420.982	397.168	404.858	482.123
58	464.503	426.07	394.793	421.036	399.358	407.343	476.779
59	468.562	425.492	390.597	417.648	398.394	403.453	478.6
60	467.508	430.505	390.213	424.697	398.533	405.318	476.561
61	465.345	424.597	389.423	421.682	403.436	409.139	476.834
62	468.997	428.432	393.52	423.375	396.295	408.027	479.319
63	467.251	426.317	392.954	418.436	402.028	407.162	476.441
64	462.229	425.53	394.633	414.459	401.731	402.879	477.583
65	467.081	427.28	392.662	416.684	399.691	407.839	483.128
66	467.005	426.671	393.209	417.193	399.321	408.418	479.111
67	469.877	426.759	391.829	418.706	399.626	407.623	484.845
68	462.527	423.875	390.584	416.91	398.237	406.515	477.58
69	466.863	428.365	393.437	415.939	402.096	405.949	480.371
70	468.667	427.937	396.069	416.52	399.727	404.488	481.107
71	469.462	427.468	393.053	419.89	400.844	405.407	480.828
72	465.149	427.999	390.65	421.928	406.117	407.581	482.194
73	464.766	428.434	393.654	421.571	411.725	406.201	478.763
74	466.903	428.206	389.709	422.755	402.534	406.206	482.402
75	464.17	424.103	390.172	420.271	403.662	407.58	483.929
76	465.815	424.707	396.145	420.682	404.358	406.799	481.155
77	467.084	422.449	397.388	421.133	404.07	403.299	479.093
78	465.989	430.904	399.552	422.959	399.172	405.833	479.521
79	466.23	428.491	394.23	421.369	410.849	408.401	482.003
80	465.845	429.43	393.146	419.822	409.045	405.29	479.438
81	462.958	431.721	392.008	422.229	406.588	403.73	480.916

82	463.895	429.204	393.554	416.579	402.805	406.178	482.358
83	464.096	424.211	387.413	419.583	411.195	404.88	482.307
84	465.446	433.559	393.783	418.255	408.168	405.551	478.478
85	466.282	426.69	391.082	420.14	407.814	406.686	478.589
86	466.418	424.9	389.819	423.252	401.374	406.481	479.773
87	464.534	426.784	390.3	424.505	406.656	408.032	476.954
88	468.106	429.256	393.947	411.343	409.982	403.85	485.195
89	467.061	428.98	392.346	420.378	411.619	407.334	484.472
90	467.844	427.378	400.298	418.68	412.256	408.439	483.145
91	467.17	426.644	394.17	413.524	402.24	407.355	484.691
92	473.052	424.83	395.481	415.528	402.581	408.15	483.489
93	467.769	428.028	398.011	415.675	406.328	408.114	480.425
94	464.831	427.177	394.557	420.522	405.854	409.347	483.245
95	467.472	427.94	394.451	415.916	407.151	404.111	480.36
96	467.284	426.571	399.01	418.88	407.283	407.799	482.777
97	466.923	428.247	398.549	419.375	404.451	406.686	483.947
98	467.864	428.406	395.737	420.792	399.483	406.615	480.404
99	469.944	427.289	392.148	420.531	412.742	406.718	479.642
100	466.402	426.981	392.221	416.894	403.022	408.86	480.117
101	469.426	423.75	392.741	414.904	405.498	407.852	481.386
102	463.656	426.426	388.858	419.053	406.376	407.734	480.318
103	467.522	430.565	396.328	422.638	396.798	403.872	480.5
104	464.722	426.291	395.923	423.108	402.441	405.698	484.267
105	467.188	432.624	393.403	410.946	409.011	408.445	483.686
106	467.152	424.234	397.848	414.813	407.051	407.884	482.769
107	464.267	423.424	393.558	411.777	407.868	407.272	479.994
108	465.021	420.721	396.445	415.212	411.948	405.576	483.357
109	467.2	425.281	395.758	416.896	401.212	403.023	480.985
110	466.572	426.775	396.689	410.768	397.485	404.439	479.735
111	467.06	428.874	392.058	412.029	403.488	409.291	480.233
112	464.476	426.242	395.603	423.705	399.138	405.813	482.751
113	465.651	427.126	395.093	421.348	399.821	407.389	481.621
114	466.199	426.642	393.598	416.425	402.761	409.229	475.829
115	464.758	425.791	398.541	408.895	402.028	406.864	484.789
116	466.299	426.371	396.322	414.299	404.059	407.798	481.894
117	469.512	424.187	398.478	417.072	402.815	407.346	478.003
118	465.119	431.246	395.434	417.021	400.132	408.15	481.816
119	467.937	431.957	393.848	418.97	408.154	410.763	478.659
120	467.25	427.619	395.07	414.484	406.467	407.685	481.892
121	466.472	427.431	393.887	415.606	398.008	410.898	483.728
122	466.463	411.701	392.925	416.543	404.639	408.751	483.51
123	467.758	427.568	399.044	417.838	403.357	407.778	480.093
124	465.295	424.564	389.968	419.591	407.271	407.323	478.624

125	466.446	434.265	393.629	419.439	403.803	404.337	479.529
126	465.743	429.052	389.994	415.246	403.019	408.93	482.201
127	468.684	425.335	395.418	418.51	406.389	406.947	478.355
128	465.261	429.679	396.81	424.944	407.146	407.129	482.055
129	469.853	428.026	392.529	423.473	400.672	404.962	480.128
130	466.979	422.171	395.538	419.558	402.643	406.698	478.827
131	465.744	428.403	394.804	416.762	392.097	405.485	481.989
132	464.994	430.585	394.995	418.141	401.595	407.106	477.14
133	464.109	428.952	395.522	420.428	395.124	407.891	479.682
134	467.136	427.021	395.857	413.904	398.009	405.293	477.49
135	465.305	426.291	395.785	422.336	402.518	409.668	477.83
136	465.959	420.857	396.564	414.048	402.336	406.474	480.055
137	470.893	428.131	398.593	418.409	406.602	406.437	479.697
138	469.474	428.304	398.389	420.381	407.985	405.667	480.183
139	471.52	428.739	394.434	415.521	401.801	405.514	480.539
140	466.688	426.524	394.278	424.233	399.981	407.44	482.078
141	467.773	424.848	391.914	417.454	402.339	410.678	476.722
142	466.318	429.542	395.58	417.255	408.6	408.076	477.045
143	468.498	427.408	396.508	420.854	401.937	404.198	482.166
144	466.911	427.931	397.249	415.747	402.844	411.029	480.742
145	467.711	424.866	399.395	415.87	412.21	406.973	481.769
146	468.383	425.599	390.146	413.333	406.697	408.227	481.903
147	471.01	428.08	393.425	416.154	403.248	407.077	480.489
148	467.64	429.451	393.642	420.836	396.132	404.87	479.074
149	468.758	428.45	397.362	413.065	398.833	403.2	480.51
150	466.594	427.118	397.619	418.475	405.031	404.303	480.312
151	467.148	427.587	392.544	412.114	397.274	405.652	475.989
152	469.172	422.381	395.297	416.281	402.94	405.428	480.754
153	470.682	428.28	399.114	415.95	397.352	406.747	480.597
154	462.856	427.475	395.767	414.695	400.305	407.916	482.376
155	468.061	428.139	399.132	409.39	398.698	406.26	479.84
156	466.585	424.134	399.655	413.149	400.165	405.651	476.839
157	464.922	420.913	394.273	413.359	401.166	404.748	480.949
158	468.65	426.221	395.907	409.82	401.811	407.217	478.499
159	462.966	426.896	396.411	411.549	394.451	409.345	478.503
160	469.504	428.529	394.264	413.872	400.066	406.985	477.992
161	465.281	427.473	394.192	418.821	407.507	409.905	481.799
162	466.443	421.887	395.018	414.338	400.432	406.136	478.702
163	464.698	424.666	390.067	416.614	403.656	403.784	478.692
164	468.58	428.985	393.064	414.911	400.814	405.237	478.182
165	464.948	425.467	393.781	413.39	401.655	407.233	478.615
166	465.641	424.294	391.504	416.473	400.802	407.014	476.948
167	469.186	422.99	395.147	413.532	404.821	405.57	477.51

168	471.563	421.323	394.742	416.262	401.117	403.313	479.724
169	466.94	429.452	394.044	414.314	398.133	405.878	477.448
170	470.446	424.596	390.309	420.676	404.316	405.192	479.027
171	467.865	429.434	391.437	416.767	401.566	409.37	478.221
172	470.974	424.149	393.538	422.135	399.153	406.647	479.778
173	467.197	428.034	393.889	417.299	404.073	405.681	480.575
174	466.978	423.426	394.601	417.363	400.792	406.222	475.572
175	465.721	426.324	395.248	417.293	403.781	404.851	478.791
176	459.089	423.495	390.242	419.681	396.536	410.1	477.085
177	467.711	422.831	391.409	416.532	393.159	406.378	480.961
178	467.75	427.269	392.561	414.236	402.066	407.001	482.219
179	469.326	425.326	395.52	417.053	404.067	408.665	478.384
180	464.457	426.918	391.555	418.171	396.959	406.646	478.93
181	464.712	424.417	396.196	420.13	399.56	407.078	477.9
182	464.828	429.712	396.071	415.265	403.891	407.606	481.115
183	467.644	425.885	395.635	417.575	408.247	406.199	475.379
184	468.315	425.539	393.07	417.439	401.997	405.356	479.515
185	467.351	428.289	398.862	426.676	402.089	405.766	475.447
186	470.482	429.513	395.599	419.162	398.171	407.609	479.867
187	468.847	428.403	389.561	415.801	406.9	407.018	479.156
188	465.713	426.032	391.495	416.391	405.815	407.752	479.012
189	471.593	423.673	392.38	420.377	407.651	405.058	481.13
190	465.432	422.676	395.44	424.85	402.873	405.945	477.539
191	467.84	427.317	392.501	420.341	400.205	408.606	479.185
192	465.125	424.243	392.345	418.128	400.798	406.159	480.515
193	466.413	425.88	394.533	422.621	403.373	405.342	477.058
194	467.418	424.898	393.193	413.927	402.218	408.145	478.44
195	465.035	422.66	391.706	423.804	399.937	410.802	475.137
196	468.576	427.975	393.911	419.853	405.637	406.727	479.149
197	467.06	427.483	395.38	416.891	395.085	407.46	476.678
198	469.527	427.418	395.949	423.613	398.486	403.5	483.474
199	465.103	426.9	393.756	421.117	402.925	409.12	482.671
200	465.805	429.397	395.489	418.379	402.333	408.074	481.364
201	470.869	431.487	391.94	421.094	400.123	410.237	481.485
202	464.813	425.05	398.32	426.003	406.919	408.199	480.745
203	466.81	423.665	394.926	417.152	398.184	408.485	480.866
204	467.535	429.106	396.126	418.343	402.728	408.562	479.192
205	469.105	427.255	398.41	419.719	413.363	406.178	479.829
206	470.408	427.422	395.333	422.69	402.651	406.477	479.589
207	467.506	428.189	394.367	423.8	401.751	407.079	478.482
208	468.655	431.811	398.79	422.165	400.382	408.123	477.276
209	466.749	423.569	392.201	422.103	400.681	404.296	474.428
210	466.865	431.747	396.159	420.181	404.949	406.336	477.604

211	465.619	428.462	394.009	420.58	399.149	408.574	483.934
212	466.612	424.856	395.886	413.294	401.981	409.978	477.224
213	467.08	427.38	395.779	418.962	402.685	407.16	479.976
214	465.34	420.927	390.393	423.141	403.156	407.853	478.495
215	468.974	423.289	393.327	416.251	402.647	407.235	482.259
216	466.781	426.988	396.709	421.2	399.91	405.532	479.713
217	466.827	424.091	394.364	420.219	409.704	404.474	481.797
218	466.949	431.699	398.659	419.918	408.161	404.959	480.4
219	469.616	430.815	389.504	420.237	405.615	405.029	477.573
220	464.251	422.196	398.284	418.538	400.275	407.442	481.514
221	468.631	429.77	395.407	420.693	405.2	407.041	479.839
222	468.844	420.436	392.748	417.728	408.209	409.083	477.264
223	466.431	420.711	394.817	417.376	407.952	407.684	478.123
224	466.006	421.286	399.481	416.588	397.981	409.437	482.202
225	465.922	423.663	391.175	419.86	399.632	409.266	479.702
226	468.022	422.641	398.963	420.999	394.716	408.533	480.739
227	465.217	422.599	394.229	417.787	399.676	405.903	479.115
228	467.843	425.658	390.835	420.501	400.202	406.309	483.93
229	465.078	425.855	391.465	421.434	401.187	405.007	480.44
230	465.149	428.587	397.499	427.385	402.053	407.7	481.153
231	467.749	431.135	395.799	416.979	403.739	407.696	479.794
232	465.713	429.529	386.853	419.331	412.753	406.435	479.957
233	466.949	430.466	398.167	415.504	409.757	407.201	479.733
234	463.656	426.804	394.71	424.378	403.084	407.739	479.19
235	462.625	426.378	395.89	421.356	396.114	406.982	479.053
236	468.256	423.578	391.899	418.46	402.353	406.419	479.848
237	469.403	425.463	394.316	418.827	399.036	406.912	480.785
238	466.92	420.562	396.82	423.704	400.304	408.268	481.48
239	467.962	424.456	392.175	418.168	392.984	405.578	482.219
240	467.608	430.039	392.654	423.691	396.916	403.287	479.571
241	465.751	431.302	397.987	418.152	396.127	404.324	481.683
242	468.231	417.59	395.793	417.741	408.334	406.365	480.641
243	468.784	424.853	397.1	420.645	401.946	405.16	480.638
244	467.119	425.3	392.767	418.483	396.615	404.145	482.374
245	462.187	424.672	398.472	416.485	397.08	407.923	479.078
246	466.913	428.684	394.763	416.737	403.098	404.9	477.419
247	464.986	428.22	396.343	412.636	404.284	407.302	479.557
248	466.92	427.73	393.217	412.903	401.994	411.438	482.648
249	466.143	430.747	397.916	421.691	406.157	408.037	477.998
250	467.533	428.839	397.342	421.878	398.722	404.362	479.854
251	466.576	428.028	395.652	416.922	403.207	408.251	480.825
252	464.816	429.592	393.248	415.666	403.041	409.735	481.347
253	467.837	432.008	396.603	415.757	400.303	404.888	479.978

254	469.449	428.225	395.892	415.2	401.106	407.552	482.34
255	466.887	431.413	397.147	418.898	398.671	409.369	482.432
256	464.554	422.328	395.561	417.161	400.434	404.226	484.031
257	466.583	431.39	392.006	417.798	400.5	407.569	483.344
258	465.771	429.093	394.405	418.468	399.935	405.863	482.244
259	463.165	428.386	397.645	414.977	402.048	405.734	480.995
260	468.757	426.816	399.972	419.699	403.488	406.076	482.886
261	467.784	430.492	399.625	419.354	402.345	407.686	483.663
262	468.802	428.646	393.795	418.833	398.479	407.599	484.36
263	468.883	425.562	394.611	417.868	400.46	404.625	481.33
264	468.914	432.612	392.648	418.653	408.147	410.152	484.284
265	463.147	431.359	393.298	418.702	403.744	406.685	482.931
266	467.397	429.236	394.436	420.023	401.039	406.81	480.279
267	466.647	429.692	396.279	420.104	404.429	404.9	479.453
268	464.847	428.36	397.301	415.496	401.69	407.835	482.502
269	468.081	427.388	398.209	422.758	401.444	406.639	482.437
270	471.128	427.087	395.171	422.823	404.753	407.895	482.833
271	467.53	426.249	396.936	417.311	400.597	407.071	480.689
272	468.92	429.175	396.266	422.74	406.575	410.09	480.767
273	467.944	418.852	396.847	420.647	408.18	407.129	482.866
274	468.99	423.745	400.01	416.765	403.243	407.036	481.246
275	465.298	426.153	396.956	428.465	402.321	407.825	476.963
276	468.291	427.538	395.84	422.405	405.494	407.534	476.681
277	463.466	425.649	394.674	423.798	401.523	406.553	479.856
278	466.34	431.356	400.637	422.448	401.975	405.409	479.714
279	466.956	429.497	395.398	421.565	406.426	406.961	479.618
280	465.459	423.799	398.199	421.388	407.556	405.767	481.467
281	464.017	432.378	396.28	415.504	403.352	405.69	479.514
282	462.321	430.771	396.601	421.023	407.443	404.449	482.079
283	465.682	425.124	397.212	420.063	408.86	398.913	481.048
284	469.937	428.038	393.844	416.958	409.26	401.454	480.562
285	467.696	422.102	403.869	420.297	410.937	403.041	478.869
286	465.01	425.503	399.674	422.685	408.072	401.523	483.56
287	467.555	432.193	398.975	420.459	388.782	400.798	478.849
288	465.268	428.155	399.483	423.611	389.761	400.133	477.521
289	466.644	430.284	402.275	423.726	388.939	401.243	481.683
290	469.025	427.759	397.737	421.617	395.68	402.649	482.585
291	467.978	430.456	398.591	420.709	400.922	406.16	481.868
292	466.622	420.792	394.06	419.675	397.731	398.342	481.333
293	469.457	431.201	397.222	418.733	390.221	406.251	480.362
294	467.385	425.737	397.668	424.653	395.756	405.166	480.465
295	468.232	431.424	399.028	417.391	388.464	405.195	480.565
296	466.836	428.08	395.012	421.402	393.591	407.4	483.237

297	469.39	420.165	396.24	415.78	390.786	406.451	482.783
298	468.984	425.845	396.612	422.683	388.801	407.248	481.785
299	465.84	427.621	394.625	419.811	388.59	406.009	483.418
300	467.055	430.405	393.817	417.172	390.183	405.972	482.35
301	467.421	429.898	396.338	420.845	393.181	405.449	480.667
302	465.992	425.391	394.563	418.152	394.391	406.156	484.05
303	470.644	430.088	397.491	425.879	389.286	408.622	482.435
304	466.747	431.406	396.595	420.831	389.559	407.069	478.752
305	466.636	426.813	399.151	421.441	393.168	405.281	481.216
306	467.748	428.327	396.426	420.679	387.418	404.51	482.013
307	464.008	429.178	393.747	416.328	387.036	407.64	477.172
308	466.3	422.126	396.52	419.607	398.408	407.573	478.669
309	466.183	425.041	396.843	416.347	389.914	405.932	480.28
310	465.197	426.832	397.867	422.45	388.171	408.815	479.493
311	467.49	425.424	396.806	417.73	390.924	410.146	478.163
312	468.05	425.058	397.652	426.237	392.209	407.639	485.047
313	466.623	427.577	395.69	419.744	390.489	404.724	484.971
314	467.387	432.237	400.4	424.195	387.814	404.251	480.325
315	467.09	427.413	393.22	423.783	390.05	402.976	481.327
316	466.548	425.62	392.587	416.077	391.998	409.519	481.871
317	469.594	427.106	396.359	424.985	393.695	405.899	481.301
318	468.409	422.299	393.946	420.5	380.599	406.071	480.092
319	464.616	430.365	395.335	419.059	394.214	407.194	480.4
320	467.967	426.438	399.559	423.573	397.351	406.988	479.604
321	469.38	429.08	397.94	422.491	394.473	403.227	480.14
322	468.669	424.427	396.322	417.894	393.182	408.712	482.264
323	468.133	428	398.493	423.819	389.178	404.66	483.117
324	465.911	425.785	395.287	422.021	392.741	409.567	480.228
325	470.336	426.864	398.933	424.048	388.644	407.539	478.971
326	464.902	427.658	394.665	420.362	388.489	406.572	482.251
327	467.474	425.084	398.507	419.693	393.374	410.209	483.887
328	465.572	425.078	393.883	417.175	398.796	408.124	485.169
329	465.443	424.805	395.205	422.034	389.279	406.36	480.857
330	466.872	431.138	395.405	418.734	395.743	405.252	483.228
331	466.592	421.47	395.125	423.304	393.255	408.355	481.689
332	467.202	425.638	396.605	416.199	388.708	408.409	484.754
333	467.78	428.81	394.52	418.162	393.267	406.519	482.975
334	464.991	431.302	398.02	418.998	392.469	403.286	481.099
335	466.767	429.844	398.435	418.487	394.871	406.551	483.701
336	464.23	424.058	400.731	424.257	386.547	408.287	482.538
337	465.953	429.276	400.222	417.666	396.373	405.015	479.598
338	466.678	426.53	396.92	423.566	394.047	406.981	481.401
339	468.742	429.662	396.186	419.489	396.404	403.705	484.025

340	468.596	431.983	396.573	417.157	395.295	405.528	484.111
341	466.521	433.551	397.291	417.664	394.713	408.158	479.109
342	468.155	433.003	395.976	424.759	390.837	408.213	481.218
343	467.214	426.019	398.388	412.156	391.642	402.915	478.576
344	467.226	424.834	392.506	416.571	394.575	407.558	481.18
345	469.033	421.267	394.113	418.741	392.878	405.669	481.22
346	467.607	432.085	393.76	417.516	392.812	406.123	480.779
347	464.584	427.95	391.429	421.856	394.451	404.645	487.16
348	467.528	433.108	392.433	417.012	395.02	407.665	476.589
349	468.001	432.535	396.102	419.803	391.297	406.187	482.65
350	468.051	428.689	397.031	420.826	398.037	407.533	479.303
351	467.394	424.21	395.644	416.387	383.61	405.958	481.364
352	466.777	431.598	400.079	418.525	385.433	405.719	480.914
353	467.586	428.714	391.855	421.475	380.438	405.076	480.912
354	466.921	433.217	395.012	413.164	395.336	405.338	479.996
355	468.472	423.088	396.438	415.925	392.904	404.015	482.4
356	467.238	418.958	399.837	414.689	399.232	405.031	480.203
357	465.449	423.808	396.772	419.842	387.733	406.033	478.962
358	468.209	423.653	395.305	419.928	395.459	405.15	482.289
359	468.038	428.838	394.794	416.062	392.659	409.165	482.736
360	466.834	428.55	392.049	421.592	394.264	405.159	482.243
361	466.859	428.795	393.473	420.775	393.733	407.616	478.856
362	466.373	425.504	400.961	420.518	391.098	401.399	482.299
363	464.741	429.087	391.109	422.161	388.554	405.914	477.941
364	467.981	431.109	395.533	420.477	391.323	409.678	480.743
365	463.868	428.473	399.942	422.89	396.23	405.266	482.337
366	469.324	427.039	396.524	418.043	391.843	403.277	482.234
367	467.99	421.982	396.363	421.73	388.592	401.959	483.685
368	467.027	423.57	393.782	424.398	397.244	407.769	480.653
369	465.358	429.812	397.389	420.179	391.99	407.246	485.254
370	466.699	421.668	400.014	418.085	391.814	404.245	484.034
371	467.076	425.6	393.836	416.983	391.871	409.022	481.367
372	461.733	427.794	395.509	423.337	388.298	406.386	481.665
373	464.764	426.557	396.839	418.217	389.994	404.498	480.119
374	466.807	433.248	394.643	420.529	386.315	408.285	480.594
375	468.084	427.319	395.946	420.014	390.961	406.383	480.913
376	464.523	429.523	393.467	420.115	394.356	406.277	479.9
377	467.505	430.583	395.34	420.72	392.458	406.691	479.181
378	467.504	424.179	393.674	419.428	385.611	404.473	481.542
379	466.339	426.227	391.875	419.87	390.633	404.965	481.029
380	466.511	424.402	394.134	420.96	392.815	408.985	479.635
381	467.779	426.533	394.545	417.587	390.006	404.986	478.263
382	463.6	429.377	397.103	423.641	394.928	408.352	481.256

383	469.587	426.073	400.869	421.435	390.71	407.637	480.272
384	470.541	430.31	393.755	419.271	401.566	407.428	479.498
385	465.199	426.792	398.694	422.449	389.676	405.614	478.513
386	467.248	429.626	391.801	417.275	387.232	407.005	481.843
387	467.466	428.239	396.675	421.264	387.674	407.472	482.203
388	463.354	428.31	395.026	423.951	389.952	407.05	482.497
389	466.403	428.097	395.334	417.744	392.906	406.143	484.548
390	465.334	429.958	395.407	418.639	382.383	404.456	479.982
391	468.379	427.248	394.589	423.716	394.677	403.724	484.459
392	468.376	427.753	395.295	421.877	390.621	402.159	482.344
393	467.42	424.405	394.932	417.059	387.173	407.505	480.928
394	468.901	423.796	398.178	419.961	389.29	407.026	480.783
395	466.27	427.538	395.29	423.259	392.584	406.632	483.618
396	467.518	434.402	392.987	415.923	394.03	406.584	484.738
397	467.658	425.431	395.75	419.202	391.458	408.292	479.479
398	468.628	428.012	396.075	423.186	389.433	407.977	480.123
399	466.077	426.883	391.79	418.365	390.697	407.009	483.071
400	467.278	430.412	397.367	422.343	389.086	405.761	482.842
401	468.785	426.583	397.313	422.147	391.393	403.641	482.064
402	468.792	430.001	396.956	420.709	394.804	407.886	483.437
403	468.778	426.816	398.687	425.033	390.813	403.578	483.262
404	468.673	426.657	393.886	421.863	394.899	405.233	480.402
405	465.829	426.466	399.047	417.611	394.325	408.297	481.315
406	465.554	427.769	395.484	422.163	392.421	404.16	477.42
407	467.28	429.235	399.499	415.611	393.147	405.728	480.549
408	463.207	426.174	396.786	420.822	393.534	407.259	478.647
409	465.603	430.4	397.482	421.415	390.465	405.812	482.477
410	469.67	420.789	398.131	417.267	390.994	408.141	483.785
411	467.391	427.437	393.794	417.905	394.573	406.337	483.178
412	465.627	422.206	397.243	418.16	392.647	406.421	481.289
413	465.264	429.329	399.043	417.725	393.86	406.283	483.712
414	468.806	433.268	402.172	421.233	393.374	405.799	480.647
415	465.539	427.09	397.147	422.047	390.238	406.732	480.177
416	466.878	431.291	393.611	417.096	395.128	407.011	480.267
417	469.443	428.188	397.877	412.722	394.987	407.841	481.539
418	466.736	428.155	398.083	415.529	394.261	405.766	479.254
419	465.079	428.434	400.073	411.315	395.576	404.047	481.077
420	468.087	432.411	396.082	417.464	394.307	407.898	485.644
421	464.642	433.721	397.275	411.277	391.792	406.456	482.999
422	465.315	428.659	396.932	420.598	389.723	409.343	485.193
423	467.121	424.406	394.376	416.764	392.757	404.715	480.859
424	468.562	418.544	401.169	422.661	391.942	405.163	481.525
425	468.772	422.36	392.24	416.774	390.745	404.353	479.51

426	464.461	419.95	394.591	425.503	391.096	401.329	482.48
427	465.505	424.785	398.173	420.413	396.882	403.014	483.374
428	465.919	429.271	397.168	418.905	386.972	408.749	482.942
429	469.353	428.386	395.108	421.188	394.537	404.808	478.416
430	465.759	423.658	396.224	416.011	390.208	407.241	482.137
431	467.488	423.808	393.23	423.64	391.289	405.152	480.528
432	467.621	426.343	396.238	418.338	395.302	405.66	481.358
433	468.537	428.097	390.606	423.185	394.37	404.882	486.072
434	469.065	427.396	390.901	422.137	395.458	406.628	482.759
435	467.981	427.545	394.362	418.712	387.344	405.391	481.302
436	468.518	430.98	394.305	419.713	395.594	402.429	482.163
437	464.262	424.205	398.617	420.65	394.162	407.147	481.322
438	465.399	430.055	396.281	421.464	397.473	405.278	480.214
439	467.61	432.591	396.085	424.339	390.754	406.396	481.97
440	466.093	428.835	397.261	419.293	387.509	408.724	483.081
441	466.901	422.701	394.134	423.385	389.484	406.419	481.739
442	468.47	423.877	395.853	419.606	390.068	404.152	483.356
443	471.884	428.356	400.1	424.732	389.72	404.314	486.208
444	470.699	424.585	393.414	420.823	390.748	406.514	482.306
445	468.582	427.391	393.75	427.779	392.077	405.338	483.9
446	469.929	427.125	392.873	421.293	390.185	404.468	478.09
447	467.064	429.272	397.33	415.621	389.299	401.854	480.384
448	470.523	421.571	395.456	419.152	394.052	405.625	482.101
449	470.104	415.921	399.88	412.287	395.866	405.06	479.894
450	465.401	425.802	396.932	410.065	394.484	407.251	482.591
451	465.364	422.751	397.727	420.323	392.042	405.996	480.626
452	467.108	429.556	398.145	421.595	392.025	405.525	478.568
453	469.592	430.818	396.687	420.861	390.539	405.086	480.577
454	467.259	422.829	397.775	417.802	386.725	406.972	483.19
455	469.715	430.249	396.872	422.076	394.52	407.864	483.926
456	466.324	425.845	398.3	417.073	395.22	405.331	482.891
457	470.026	433.611	401.56	420.463	392.838	407.351	483.221
458	466.172	423.518	391.383	417.5	396.356	407.314	479.492
459	465.694	424.693	397.024	415.881	397.265	409.352	478.55
460	467.705	423.805	394.788	418.491	388.591	403.573	478.894
461	466.046	426.557	399.745	415.917	389.995	403.296	484.652
462	469.026	427.522	399.596	417.847	392.531	402.895	482.853
463	469.129	424.554	395.732	421.091	392.097	403.238	482.022
464	465.616	430.714	396.385	421.584	388.108	408.69	480.96
465	465.062	428.792	395.105	414.996	392.926	407.57	483.049
466	463.563	421.739	395.652	417.394	391.132	408.975	480.115
467	469.821	426.463	392.653	420.867	393.792	407.157	480.696
468	466.273	423.355	394.594	419.794	387.442	405.547	482.509

469	468.686	429.118	400.74	417.62	388.708	405.15	482.02
470	471.128	421.52	400.584	415.816	393.231	408.263	481.46
471	465.141	428.967	395.046	414.764	391.574	405.281	479.96
472	468.187	425.979	395.537	418.55	386.377	406.594	479.304
473	466.933	421.794	396.874	419.584	391.878	406.716	482.506
474	465.676	422.26	389.145	422.707	393.448	406.936	483.36
475	465.445	422.386	392.611	422.869	387.14	406.934	480.76
476	466.07	428.649	394.615	422.473	395.885	407.061	479.356
477	465.186	429.919	390.8	416.584	395.936	404.743	483.001
478	467.722	428.539	399.732	416.773	392.346	406.484	479.641
479	469.284	426.047	398.735	423.235	387.788	406.077	481.617
480	464.326	432.57	401.667	417.781	388.341	406.585	476.811
481	464.881	426.452	395.851	421.074	392.328	408.731	481.866
482	464.837	421.04	394.185	415.271	391.17	407.585	483.11
483	469.114	427.11	398.679	417.311	395.806	409.262	479.542
484	468.139	430.295	400.667	421.918	397.526	411.62	481.932
485	467.91	424.402	396.213	419.765	393.366	407.203	480.874
486	467.874	427.901	395.241	419.844	392.25	407.563	482.617
487	468.313	433.931	399.762	414.888	395.432	407.146	481.648
488	470.028	430.86	395.808	417.862	393.414	409.136	483.306
489	467.078	429.905	398.479	418.503	397.309	407.768	481.464
490	470.191	428.966	394.156	416.026	390.305	408.133	488.276
491	465.211	427.331	395.65	425.399	390.418	404.457	481.002
492	467.657	422.841	397.913	411.507	392.156	405.609	482.293
493	466.391	420.444	399.976	421.936	388.439	406.464	479.893
494	469.778	424.017	392.666	414.523	390.312	407.761	481.371
495	469.562	431.293	397.221	419.406	394.303	404.996	480.11
496	468.025	429.805	393.763	416.067	396.32	404.002	480.543
497	468.167	425.796	390.678	414.844	392.838	405.823	479.717
498	467.068	430.879	397.057	417.56	390.98	409.046	481.631
499	467.596	426.333	394.646	417.667	390.558	405.821	476.441
500	467.532	425.894	394.708	416.535	396.912	405.688	482.642
501	469.349	431.357	395.794	416.877	390.539	404.152	482.548

BNS1

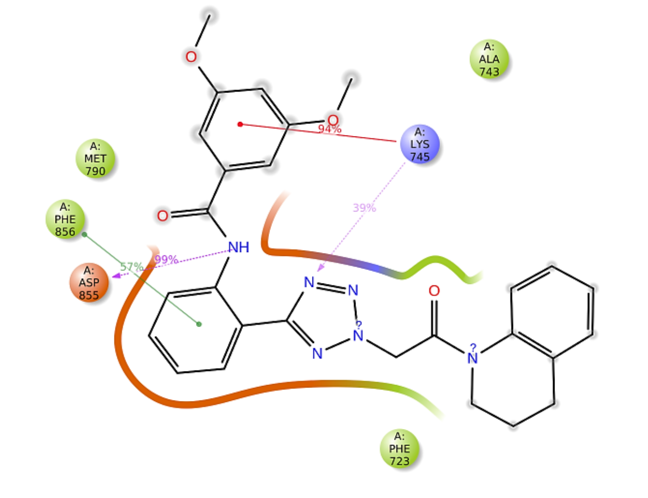

BNS2

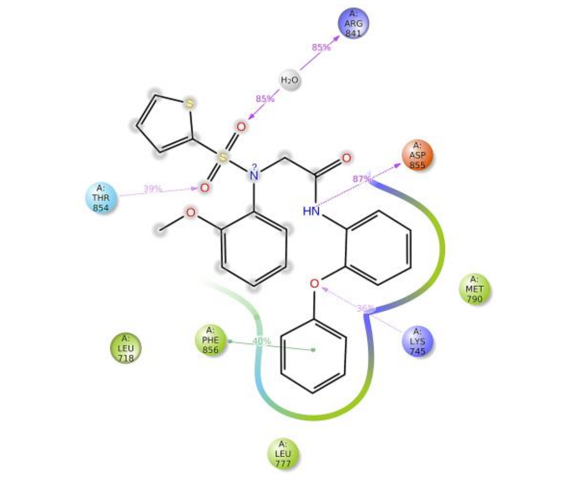

BNS3

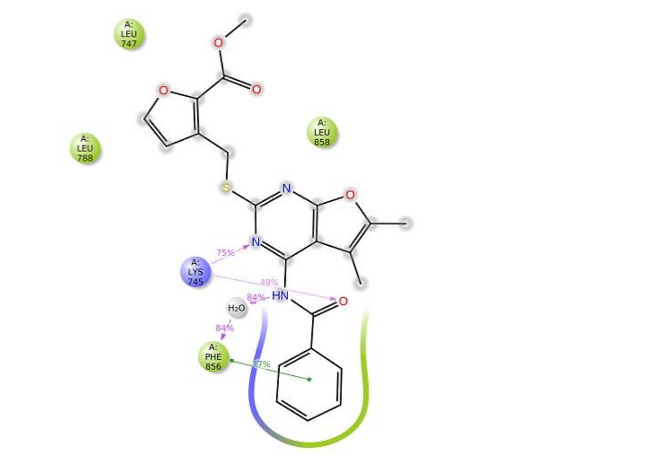

BNS4

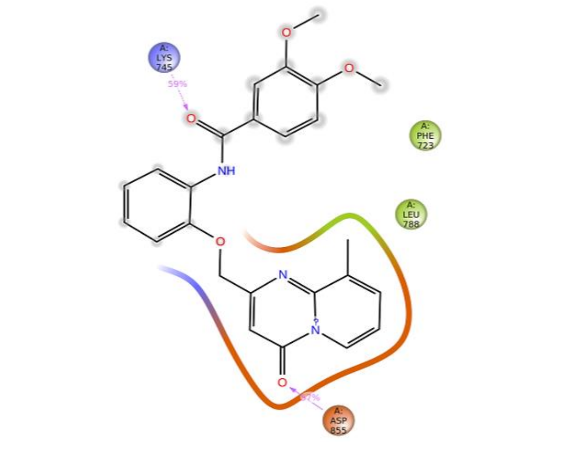

BNS11

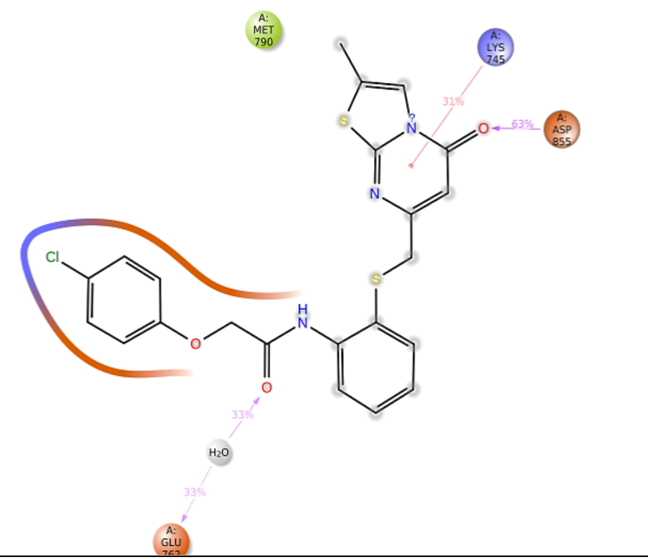

BNS16

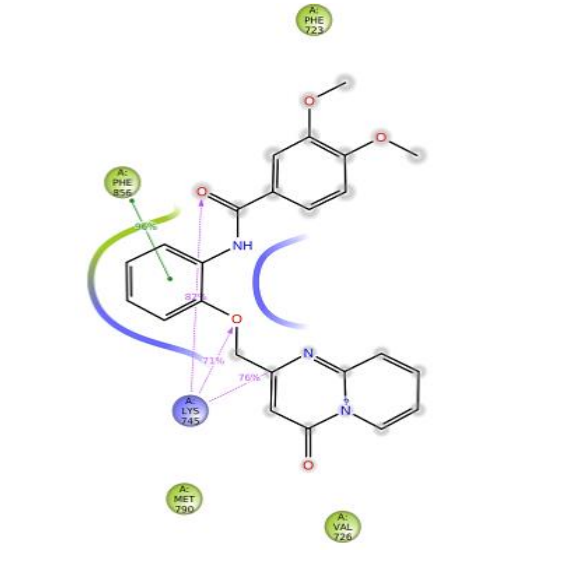

JBJ-125

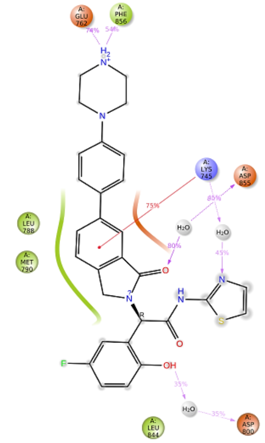

Figure S1. Post MD simulation Interaction Profile.

BNS2

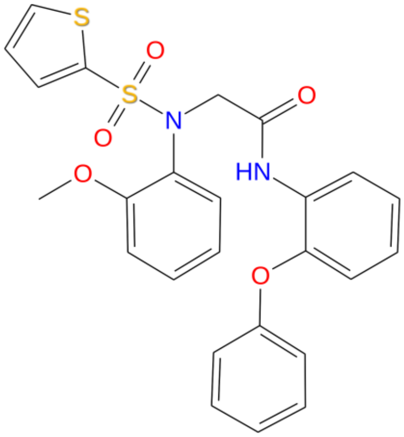

BNS16

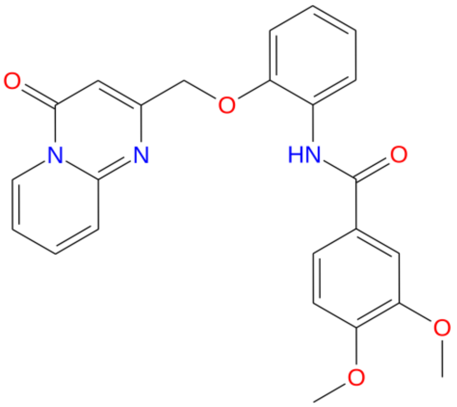

BNS1 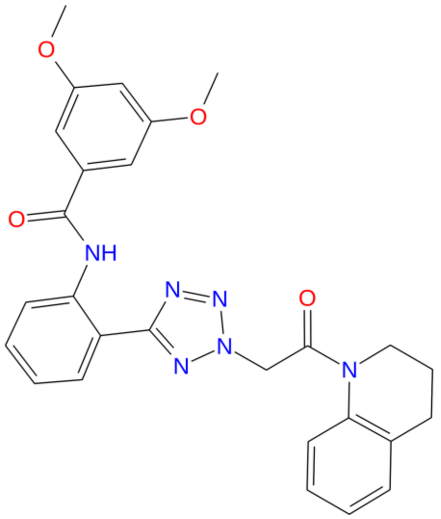	BNS3 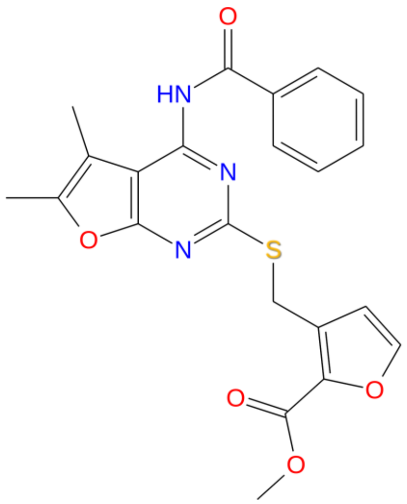
BNS11 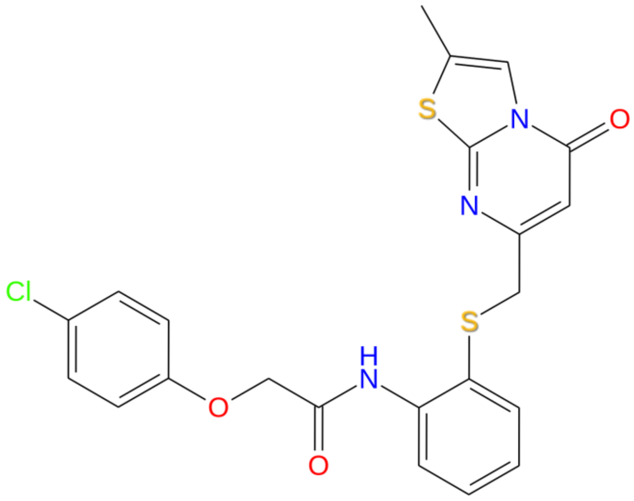	

Figure S2. Molecular Representation of final compounds
